# Rama: a machine learning approach for ribosomal protein prediction in plants

**DOI:** 10.1038/s41598-017-16322-4

**Published:** 2017-11-24

**Authors:** Thales Francisco Mota Carvalho, José Cleydson F. Silva, Iara Pinheiro Calil, Elizabeth Pacheco Batista Fontes, Fabio Ribeiro Cerqueira

**Affiliations:** 1 Computer Science Department, Universidade Federal de Viçosa, 36570-900 Minas Gerais, Brazil; 2 National Institute of Science and Technology in Plant-Pest Interactions/BIOAGRO, Universidade Federal de Viçosa, 36570-900 Minas Gerais, Brazil; 3 Department of Production Engineering, Universidade Federal Fluminense, Petrópolis, 25650-050 Rio de Janeiro, Brazil

## Abstract

Ribosomal proteins (RPs) play a fundamental role within all type of cells, as they are major components of ribosomes, which are essential for translation of mRNAs. Furthermore, these proteins are involved in various physiological and pathological processes. The intrinsic biological relevance of RPs motivated advanced studies for the identification of unrevealed RPs. In this work, we propose a new computational method, termed Rama, for the prediction of RPs, based on machine learning techniques, with a particular interest in plants. To perform an effective classification, Rama uses a set of fundamental attributes of the amino acid side chains and applies a two-step procedure to classify proteins with unknown function as RPs. The evaluation of the resultant predictive models showed that Rama could achieve mean sensitivity, precision, and specificity of 0.91, 0.91, and 0.82, respectively. Furthermore, a list of proteins that have no annotation in Phytozome v.10, and are annotated as RPs in Phytozome v.12, were correctly classified by our models. Additional computational experiments have also shown that Rama presents high accuracy to differentiate ribosomal proteins from RNA-binding proteins. Finally, two novel proteins of *Arabidopsis thaliana* were validated in biological experiments. Rama is freely available at http://inctipp.bioagro.ufv.br:8080/Rama.

## Introduction

Ribosomal proteins (RPs) of different sizes are associated with ribosomal RNAs (rRNAs) to make up ribosomes, which are part of an important cellular machinery responsible for protein synthesis^1^. The ribosome structure comprises two subunits, the small subunit and the large subunit, the latter being approximately twice as large as the former. The association of ribosomes with messenger RNAs (mRNAs) along with charged tRNAs allows the synthesis of proteins, which are vital for cellular activities. Moreover, RPs are involved in several physiological and pathological processes^2–4^. For example, RPs have been shown to trigger a suppression pathway for p53 tumors as a response to ribosomal stress^2,3^. Other important roles of RPs inside the cell have also been reported, such as effectors of antiviral response in plants^5^. Furthermore, the ribosome assembly has been shown to play important roles in embryonic genome activation (EGA) at the 8-cell stage^6^. Identifying new ribosomal proteins may contribute to the understanding of how ribosomes work and the discovery of new biological functions. This study is particularly focused on plant ribosomal proteins.

In genomics, metagenomics, and proteomics, the functional annotation of genes and proteins relies on a large number of computational tools to identify specific functions or domains, such as active sites, functional domains, gene families, physical structures, or subcellular localization. The software InterProScan is a major computational tool used in functional genomics, and is interconnected with the main databases to carry out functional analysis of proteins^7^. InterProScan searches for protein signatures using methods classified into two main modalities. The first one includes algorithms, such as TMHMM, SignalP, and Phobius, which work individually to check for any characteristic in the protein, e.g., membrane segments and signal peptide regions. The methods in the second modality use algorithms, such as BLAST and hidden Markov models (HMM), to carry out searches for sequence alignments and perform protein associations accessing a broad range of databases. The user is provided with the result of a post-processing step that matches output information produced by those procedures. InterProScan features global functional annotation, and its analyses enable identifying, whenever possible, gene ontology by associating terms from the Gene Ontology Consortium^8^.

Although InterProScan is a robust tool to classify proteins in terms of function, its technique is limited to the used databases. This impairs the prediction of proteins that have no phylogenetic conservation or unknown functional domains. As a result, creating new computational methods to predict novel proteins is required to complement the comparative analysis. Machine learning (ML)-based methods have been applied for characterizing proteins, e.g., post-translational modification prediction^9,10^, ligand-protein interaction prediction^11^, and protein complex prediction^12^ as well as prediction of functional families of proteins, such as disease resistance proteins in plants^13^.

In the present research, we propose a new *in silico* technique for predicting ribosomal protein in plants, termed here Rama, based on ML methods. Our approach uses characteristics common to any protein to build classification models. In this study, six organisms were selected to make up the training sets (TSs): two species of monocotyledons, *Zea mays* (*Z*. *mays*) and *Oryza sativa* (*O*. *sativa*); three species of dicotyledons, *Arabidopsis thaliana* (*A*. *thaliana*), *Solanum lycopersicum* (*S*. *lycopersicum*), and *Glycine max* (*G*. *max*); and one species of phytoplankton, *Ostreococcus lucimarinus* (*O*. *lucimarinus*). As both RPs and histone proteins (HPs) display binding affinity to nucleic acids and similar amino acid properties, two training sets were created for each species, one containing RPs and non-ribosomal proteins (NRPs), and the other containing RPs and HPs.

The amino acid sequences of the above-mentioned species were obtained from the repository Phytozome v.10^14^. Once the training sets were determined, six ML algorithms were applied to build the predictive models: Multilayer Perceptron (MLP), Random Forest (RF), Naive Bayes (NB), LogitBoost, J48 decision tree (a Java implementation of C4.5), and Support Vector Machines (SVM). For the learning process of the latter, we used the Sequential Minimal Optimization (SMO) algorithm^15,16^. The best resultant models were selected to comprise the Rama pipeline.

The prediction method hereby proposed performs two classification steps. Initially, sequences of proteins of unknown function are given as input to ML models trained with ribosomal and non-ribosomal proteins (RPs/NRPs). In the second step, the positively classified sequences undergo a new classification step using models trained with ribosomal and histone proteins (RPs/HPs). The sequences classified positively in both steps are considered ribosomal proteins. We performed evaluation tests on the resultant ML models, including an additional computational experiment to demonstrate the accuracy of Rama in distinguishing ribosomal proteins from RNA-binding proteins (RBPs). Our results indicate that our machine learning approach is effective to predict new ribosomal proteins and might be an important complement to the classical homology-based methods. Another important observation is that the running time of Rama is approximately 600 times faster compared with InterProScan.

## Results and Discussion

### Computational experiments

To conduct an *in silico* evaluation of Rama, six computational experiments were performed: (i) evaluation of the importance of each attribute using Information Gain (IG) and density plots; (ii) inter-species tests, jackknife tests, and 10-fold cross validations to assess the ability of the resultant ML models to distinguish between RPs/NRPs; (iii) inter-species tests, jackknife tests, and 10-fold cross validations to assess the ability of the resultant ML models to distinguish between RPs and HPs; (iv) tests using the complete method proposed in Rama to measure the performance of the system as a whole, i.e., the performance of the combined ML models; (v) checking the capacity of Rama to differentiate ribosomal proteins from RBPs; and (vi) checking whether Rama is able to identify a set of proteins annotated as RPs in Phytozome v.12 using the models created from proteins of Phytozome v.10. It is important to notice that such RPs from version 12, used as a test set, have no function associated in version 10.

#### Attribute analysis

The first experiment allows examining the importance of the attributes for a better understanding of their influence in RP classification. These analyses were performed through the IG method and density plots for visualizing the distributions of attribute values over the classes.

Tables 1 and 2 present the values obtained from the IG method for the RPs/NRPs and RPs/HPs datasets, respectively. In addition to the IG values, these tables describe the position of each attribute in the ranking formed from the IG scores, thereby identifying the attributes with the greatest influence on the classification process. When the ranking is inspected, although differences may be observed among the analyzed species, the similarities related to top-ranked attributes are noteworthy. In most cases, the two best attributes in one species coincide with the two best attributes in another species. For example, the table that displays the IG ranking for the RPs/NRPs datasets shows that the attributes ‘Positively charged’ and ‘Length’ obtained the best values in all species. In the table of IG values for the RPs/HPs, those attributes were not unanimously the best ones, but they still stand out in general. The importance of the attribute ‘Positively charged’, in particular, is consistent with the binding affinity of ribosomal proteins to RNAs. The attribute ‘Length’, in turn, also contributes strongly to characterize the protein types.

**Table 1:** IG values for the datasets composed of RPs and NRPs.

Attribute	*A*. *thaliana*	*G*. *max*	*O*. *sativa*	*O*. *lucimarinus*	*S*. *lycopersicum*	*Z*. *mays*
Aromatic	0.0949 (4°)	0.0509 (4°)	0.0307 (8°)	0.0249 (8°)	0.0622 (5°)	0.00987 (9°)
Hydrophobicity	0.0554 (7°)	0.0418 (7°)	0.0623 (5°)	0.0649 (5°)	0.0670 (4°)	0.05021 (5°)
Molecular mass	0.0313 (8°)	0.0299 (8°)	0.0655 (4°)	0.0439 (6°)	0.0127 (8°)	0.06119 (4°)
Negatively charged	0.0741 (5°)	0.0462 (6°)	0.0579 (6°)	0.1239 (3°)	0.0457 (6°)	0.02959 (7°)
Nonpolar aliphatic	0.0000 (9°)	0.0000 (9°)	0.0210 (9°)	0.0260 (7°)	0.0000 (9°)	0.02596 (8°)
Polar uncharged	0.1196 (3°)	0.1356 (3°)	0.0468 (7°)	0.0000 (9°)	0.0980 (3°)	0.03457 (6°)
Positively charged	0.3450 (1°)	0.3123 (1°)	0.3284 (1°)	0.3107 (1°)	0.3076 (1°)	0.26796 (1°)
Length	0.2832 (2°)	0.2365 (2°)	0.2501 (2°)	0.2245 (2°)	0.1909 (2°)	0.14375 (2°)
Volume	0.0600 (6°)	0.0500 (5°)	0.0956 (3°)	0.0676 (4°)	0.0224 (7°)	0.08408 (3°)

The values were obtained by running the IG method in each training set. The rank created with IG values, shown in parentheses, highlights the importance of each attribute for each species.

**Table 2:** IG values for datasets composed of RPs and HPs.

Attribute	*A*. *thaliana*	*G*. *max*	*O*. *sativa*	*O*. *lucimarinus*	*S*. *lycopersicum*	*Z*. *mays*
Aromatic	0.0000 (8°)	0.0733 (5°)	0.0000 (6°)	0.0000 (4°)	0.0357 (4°)	0.0431 (7°)
Hydrophobicity	0.0495 (6°)	0.0106 (9°)	0.0193 (5°)	0.0000 (4°)	0.0000 (6°)	0.0252 (9°)
Molecular mass	0.0758 (5°)	0.0607 (7°)	0.0576 (4°)	0.0000 (4°)	0.0582 (3°)	0.0961 (3°)
Negatively charged	0.1046 (4°)	0.0887 (4°)	0.0000 (6°)	0.0619 (3°)	0.0000 (6°)	0.0864 (4°)
Nonpolar aliphatic	0.0000 (8°)	0.0398 (8°)	0.0000 (6°)	0.0000 (4°)	0.0000 (6°)	0.0315 (8°)
Polar uncharged	0.0324 (7°)	0.0888 (3°)	0.0000 (6°)	0.0000 (4°)	0.0251 (5°)	0.0507 (6°)
Positively charged	0.1218 (2°)	0.1261 (2°)	0.0945 (2°)	0.0930 (2°)	0.0000 (6°)	0.1560 (1°)
Length	0.1094 (3°)	0.2398 (1°)	0.1279 (1°)	0.2072 (1°)	0.1559 (1°)	0.0526 (5°)
Volume	0.1742 (1°)	0.0654 (6°)	0.0743 (3°)	0.0000 (4°)	0.0687 (2°)	0.1268 (2°)

The values were obtained by running the IG method in each training set. The rank created with IG values, shown in parentheses, highlights the importance of each attribute for each species.

Although there are attributes with zeroed IG value, removing them causes a slight negative variation in the performance of the models (not shown). This occurs because IG assesses the importance of an attribute independently, i.e., not in conjunction with other attributes. Therefore, the performance decay after removing one such attribute shows that the combined effect of the attributes is important for the classification task. Furthermore, no attribute presented IG equal to zero for all examined species. Also, the nine attributes used for the classification tasks comprise already a small set, meaning no risk of curse of dimensionality or slow model building. As a result, we decided to keep all nine attributes for the predictive procedures.

**Figure 1:**
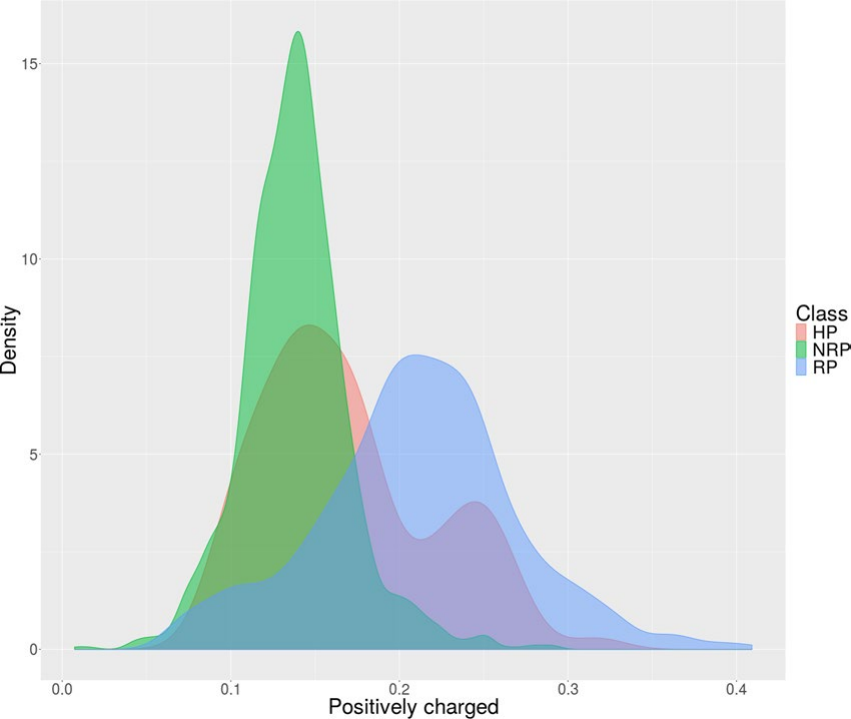
Density plots for the attribute ‘Positively charged’ in *A*. *thaliana* datasets.

In addition to the analysis with IG, density plots were produced to provide a visualization of the distribution of each attribute over the three classes (RP/NRP/HP). Figure 1 shows such plots for attribute ‘Positively charged’ in *A*. *thaliana* data. The values of that attribute present distinct distributions when the three classes are compared, which is an evidence that the attribute contributes to distinguish instances of different classes. Supplementary Figures S1, S2, S3, S4, S5, and S6 depict the complete set of density plots for all attributes in all datasets of the six species covered here. These figures, in general, display characteristics similar to the plots of Fig. 1, i.e., for each attribute, different distributions are perceived comparing distinct classes. This result, thus, reinforces the decision of keeping all nine attributes.

#### Assessing the ability of the ML models to distinguish RPs from NRPs

In the second experiment, we assessed the predictive power of the models created with the RPs/NRPs datasets. In this case, three types of validation tests, inter-species tests, jackknife tests, and 10-fold cross validations, were performed. This experiment allowed to evaluate the generality of the models that classify RPs/NRPs (first stage), using the well-known measures: accuracy, sensitivity, precision, F-measure, specificity, and Matthews correlation coefficient (MCC). In an inter-species test, once a test species is defined, it is left out of the training process and every model built with sequences of each one of the other five species is assessed using that test data. In the jackknife test and 10-fold cross validation, the model created with sequences of a certain species is tested with sequences of the same dataset (same species), but varying the partitions of training and test, according to the cross validation technique^17,18^. The results of all tests can be seen in Supplementary Table S1, which shows the performance of each individual model built for every species. A compilation of these results is presented in Table 3, which contains the mean performance measured in the inter-species tests, jackknife tests, and 10-fold cross validations.

**Table 3:** Summarized results of the classification models built with the RPs/NRPs datasets.

Test species	Accuracy	Sensitivity	Precision	F-measure	Specificity	MCC
*A*. *thaliana*	0.93	0.93	0.92	0.92	0.84	0.80
	0.94	0.94	0.94	0.94	0.87	0.84
	0.94	0.94	0.94	0.94	0.88	0.84
*G*. *max*	0.90	0.90	0.90	0.90	0.79	0.74
	0.94	0.94	0.94	0.94	0.89	0.84
	0.94	0.94	0.94	0.94	0.88	0.84
*O*. *lucimarinus*	0.90	0.90	0.90	0.90	0.82	0.74
	0.92	0.92	0.91	0.91	0.83	0.78
	0.91	0.91	0.91	0.91	0.81	0.76
*O*. *sativa*	0.90	0.90	0.90	0.90	0.83	0.75
	0.92	0.92	0.92	0.92	0.83	0.79
	0.92	0.92	0.92	0.92	0.84	0.79
*S*. *lycopersicum*	0.90	0.90	0.89	0.89	0.79	0.72
	0.90	0.90	0.90	0.90	0.79	0.73
	0.90	0.90	0.90	0.90	0.80	0.74
*Z*. *mays*	0.86	0.86	0.86	0.86	0.76	0.64
	0.90	0.90	0.90	0.90	0.79	0.73
	0.90	0.90	0.90	0.90	0.79	0.74

For each tested species, the first line refers to the mean values obtained in the inter-species tests, i.e., the tests where the ML models are trained with proteins of the other five species (see complete results in Supplementary Table S1), while the second and third lines present the results of a 10-fold cross validation and a jackknife test, respectively.

For the inter-species tests, when *A*. *thaliana* sequences were used as a test set, the models built for the other species achieved mean accuracy, sensitivity, precision, F-measure, specificity, and MCC of 0.93, 0.93, 0.92, 0.92, 0.84 and 0.80 respectively (Table 3; Supplementary Table S1). The inter-species results for the other species also reached satisfactory values. Accuracy, sensitivity, F-measure, and precision presented values ≥ 0.86, while specificity varied from 0.76 to 0.83, and MCC varied from 0.64 to 0.74 (Table 3; Supplementary Table S1). The jackknife tests and 10-fold cross validations presented values ≥ 0.90 for accuracy, sensitivity, F-measure as well as precision, and values varying from 0.79 to 0.89 for specificity. MCC varied from 0.73 to 0.84 (Table 3; Supplementary Table S1).

#### Assessing the ability of the ML models to distinguish RPs from HPs

The third experiment is analogous to the second one. In this case, however, we assessed the generality of the models constructed from the datasets composed of RPs/HPs. Again, we show the outcome of inter-species tests, jackknife tests, and 10-fold cross validations. Supplementary Table S2 displays the entire result and Table 4 presents a summarization.

**Table 4:** Summarized results of the classification models built with the RPs/HPs datasets.

Test species	Accuracy	Sensitivity	Precision	F-measure	Specificity	MCC
*A*. *thaliana*	0.87	0.87	0.88	0.85	0.66	0.65
	0.94	0.94	0.94	0.94	0.88	0.86
	0.94	0.94	0.94	0.94	0.87	0.85
*G*. *max*	0.89	0.89	0.89	0.89	0.69	0.65
	0.96	0.96	0.96	0.96	0.87	0.87
	0.96	0.96	0.96	0.96	0.87	0.87
*O*. *lucimarinus*	0.86	0.86	0.85	0.85	0.60	0.54
	0.89	0.89	0.89	0.89	0.73	0.66
	0.90	0.90	0.90	0.90	0.75	0.69
*O*. *sativa*	0.91	0.91	0.91	0.91	0.73	0.69
	0.94	0.94	0.93	0.93	0.76	0.77
	0.94	0.94	0.94	0.93	0.77	0.78
*S*. *lycopersicum*	0.88	0.88	0.88	0.88	0.65	0.62
	0.91	0.91	0.91	0.90	0.68	0.70
	0.91	0.91	0.91	0.90	0.68	0.71
*Z*. *mays*	0.85	0.85	0.84	0.83	0.59	0.52
	0.91	0.91	0.91	0.91	0.77	0.75
	0.92	0.92	0.91	0.91	0.78	0.75

For each tested species, the first line refers to the mean values obtained in the inter-species tests, i.e., the tests where the ML models are trained with proteins of the other five species (see complete results in Supplementary Table S2), while the second and third lines present the results of a 10-fold cross validation and a jackknife test, respectively.

Table 4 shows that the inter-species tests for *A*. *thaliana* led to a mean value of 0.87 for accuracy and sensitivity, 0.88 for precision, 0.85 for F-measure, 0.66 for specificity, and 0.65 for MCC. The inter-species tests for the other species resulted in a minimum accuracy, sensitivity, precision, and F-measure of 0.85, 0.85, 0.84, and 0.83, respectively, while specificity varied from 0.59 to 0.73, and MCC ranged from 0.52 to 0.69 (Table 4; Supplementary Table S2).

Table 4 also shows the jackknife test and cross validation results. The models obtained, on average, 0.92 for accuracy, sensitivity, precision as well as F-measure, 0.78 for specificity, and 0.77 for MCC (Table 4; Supplementary Table S2). Similarly to the models built from RPs/NRPs datasets, the jackknife and cross validation led to better results, in general, when compared with the inter-species tests. This is expected because the proteins for training and test in a cross validation evaluation are from the same species.

Although the values for specificity were not as satisfactory as the other measures, Rama achieved high values for sensitivity and precision using the resultant models of RPs/NRPs and RPs/HPs (72 models presented in Supplementary Tables S1 and S2, and summarized in Tables 3 and 4), making up an important feature of the method. This performance implicates that (i) few RPs would be missed (high sensitivity), and (ii) among the ones predicted as positives, few of them would be false positives (high precision), i.e., financial resources and time would be saved in *in vitro* validations. Additionally, we show next that when the entire Rama pipeline is applied, all performance measures are kept high, indicating that the ML models used in a combined way is another important characteristic of our method.

#### Assessing the pipeline as a whole

**Figure 2:**
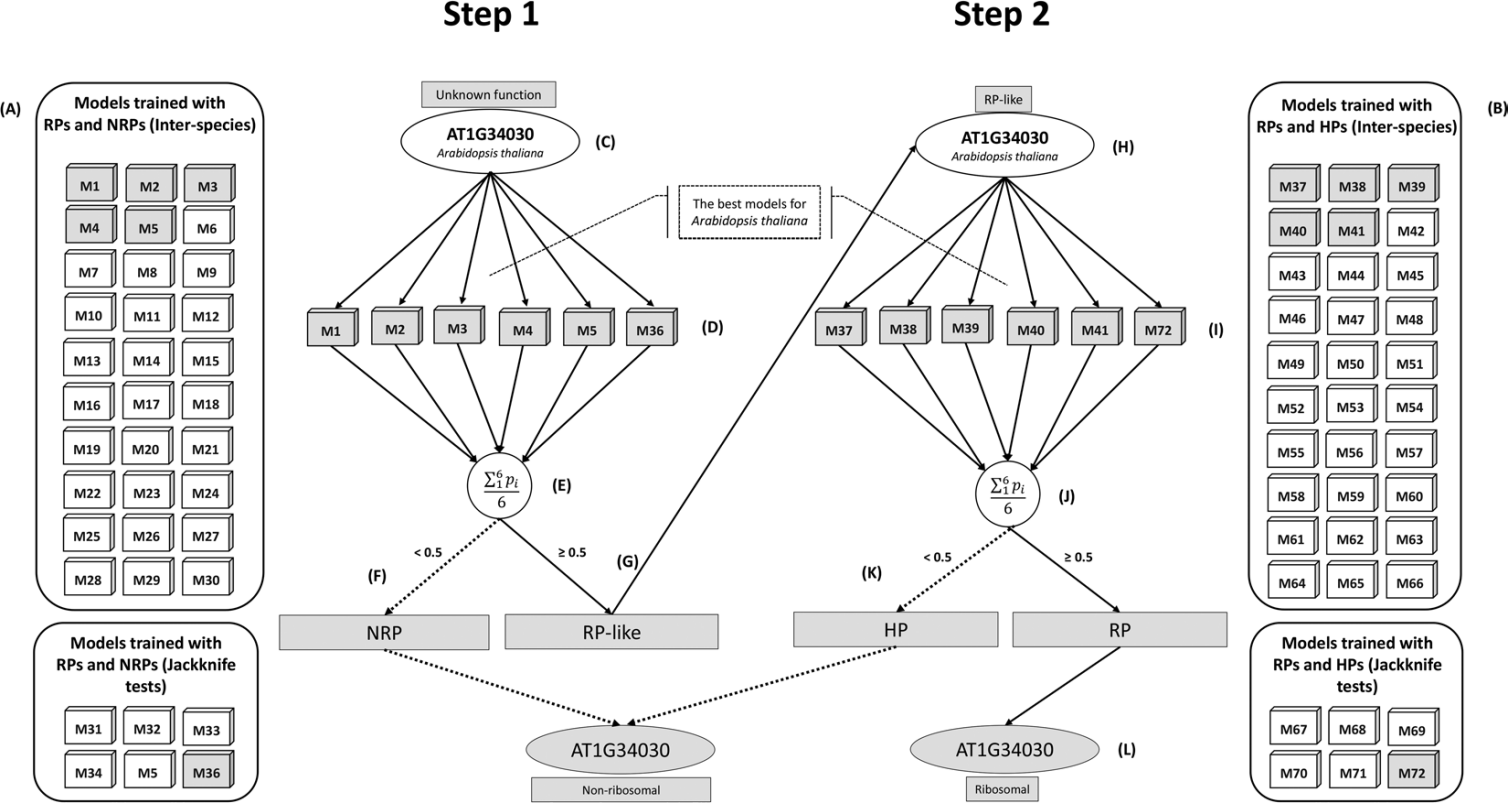
Illustration of the Rama method using a sequence of *A*. *thaliana* as an example. The proposed approach is described in 12 parts. In A and B, the built ML models are depicted. Parts C, D, E, F, and G comprise step 1 of Rama. In C, it is shown the input (amino acid sequence and species, e.g., *A*. *thaliana*) to the models for RPs/NRPs classification, according to the selected species. In D, the input protein is subjected to these models. In E, the probabilities generated by the models are computed according to Equation 9, producing either class 0 or class 1. In case of class 0 (mean probability < 0.5), i.e., not a ribosome protein (F), the program stops and informs the result. In case of class 1 (mean probability ≥ 0.5), i.e., a ribosome-like protein (G), the same amino acid sequence is used as input to the step 2 of the method, which is illustrated in parts H, I, J, K, and L. In H, the amino acid sequence is now given as input to the classification models for RPs/HPs (I). In J, the probabilities of these models are aggregated to generate the final classification. It can be either 0, classified as a histone protein (K), or 1, classified as a ribosomal protein (L). The default discriminant probability 0.5 can be altered.

As a fourth *in silico* experiment, we tested the ML models in a combined approach, as depicted in Fig. 2, i.e., running the complete pipeline implemented in Rama. It is important to remember that a protein sequence is considered RP if it passes through the two stages with enough probability. Thus, Rama applies not only an ensemble approach in each stage, but also generates a final output that is a consequence of the outcomes of both stages. It is expected that this combination of a series of ML models keeps high the overall predictive power of the method.

Notice that when our computational tool is used to classify a sequence of a certain species, the model constructed using the proteins of this species can (and should) be used. However, to provide evidence of the robustness of Rama, our experiment using the whole pipeline did not include the model created with the sequences of the species being tested. Table 5 shows the performance of Rama. The values in the first line regarding *A*. *thaliana*, for instance, are a result of the application of models created from *S*. *lycopersicum*, *Z*. *mays*, *O*. *lucimarinus*, *G*. *max*, and *O*. *sativa* for both stages (i.e., models M1, M2, M3, M4, M5, M37, M38, M39, M40, and M41 shown in Supplementary Tables S1 and S2). In this case, the performance measures could in fact be maintained high. The values for specificity are particularly noteworthy if compared with values of individual models for classifying RPs/HPs (Table 4). Rama could achieve mean values for accuracy, sensitivity, precision, F-measure, specificity, and MCC of 0.91, 0.91, 0.91, 0.91, 0.82 and 0.77, respectively. Very importantly, the final pipeline is composed of ML models built mostly with RF and MLP (as can be seen in Supplementary Tables S1 and S2), as these algorithms outperformed SMO (with or without a kernel function) and the other three ML algorithms in all tests (see primary experiments reported in Supplementary Table S3). The only exception was model M48 (see Supplementary Table S2) built using the Naive Bayes approach. Only in this case, NB could overcome RF, MLP, and the other classification methods we tested.

**Table 5:** Results obtained by applying the whole pipeline of Rama.

Test species	Accuracy	Sensitivity	Precision	F-measure	Specificity	MCC
*A*. *thaliana*	0.93	0.93	0.93	0.93	0.85	0.82
*Z*. *mays*	0.87	0.87	0.87	0.87	0.76	0.66
*O*. *sativa*	0.93	0.93	0.93	0.93	0.85	0.81
*O*. *lucimarinus*	0.92	0.92	0.92	0.93	0.85	0.80
*G*. *max*	0.92	0.92	0.92	0.92	0.83	0.80
*S*. *lycopersicum*	0.91	0.91	0.90	0.90	0.80	0.75
Mean	0.91	0.91	0.91	0.91	0.82	0.77

For each species, the values are a result of the application of the whole pipeline using the ML models built for both stages with sequences of the other five species.

#### Assessing the ability of Rama to distinguish RPs from RBPs

In a fifth computational experiment, we evaluated the capacity of Rama to differentiate RPs from other proteins that associate with RNA, such as RNA-Binding proteins. In this experiment, we selected RBPs from the six species covered in this work, aiming to evaluate the capacity of Rama in classifying such proteins as NRPs. Rama correctly classified 94.93% of the 355 RBPs, presented in Supplementary Table S4, as NRPs. The result of this experiment shows that the models in our system are able to distinguish RPs from other types of proteins with similar binding activity, not only from HPs.

#### Additional assessment of Rama using a test set extracted from Phytozome v.12

In a sixth computational experiment, we used four proteins of *A*. *thaliana* with unknown function in Phytozome v.10, described as RPs in Phytozome v.12, as a test set for the Rama pipeline. The models built for our pipeline used proteins of Phytozome v.10 as training examples, i.e., the tested proteins of Phytozome v.12 did not make part of the learning process. The tested proteins were AT3G02080.1, AT5G15520.1, AT5G61170.1, and ATCG00760.1. Rama correctly classified all of them as RPs. Thus, the proposed method was capable of predicting a change that occurred when Phytozome was updated to version 12.

### Two novel RPs predicted by Rama displayed RNA binding activity consistent with the typical ribosomal function of RPs

**Figure 3:**
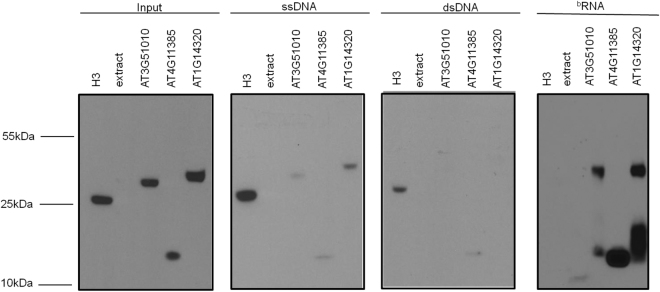
Nucleic acid binding activity of novel predicted RPs. HA fused-Histone H3, HA fused-ribosomal protein RPL10 (AT1G14320), and the novel predicted RPs AT3G51010 and AT4G11385 (also fused to HA) were *in vitro* transcribed/translated (input), incubated with sepharose bound-ssDNA, sepharose bound-dsDNA or *A*. *thaliana* streptavidin-beads bound-biotinylated RNA (bRNA), pulled-down, separated by SDS-PAGE, and immunoblotted with anti-HA serum. Input shows an immunoblot of the *in vitro* transcribed/translated HA fusions. The sizes and positions of protein molecular mass markers are shown on the left.

We searched for novel RPs by giving unknown protein sequences from *A*. *thaliana* to Rama as input, using the 12 models (six for the first stage plus six for the second stage). Supplementary Table S5 lists the top 50 ranked protein sequences displayed in the Rama output. Next, we selected two proteins, the one with highest probability and another one selected randomly among the list of 50, identified by AT3G51010 and AT4G11385, respectively, for further *in vitro* analyses. As fundamental components that make up and stabilize the structure of ribosomes, many ribosomal proteins contain characterized RNA binding domain, which is often a surface-exposed domain interacting with multiple RNA elements within rRNA^19,20^. We designed an *in vitro* nucleic acid binding assay to further confirm that the new predicted RPs would share common biochemical properties of ribosomal proteins (Fig. 3). We also included in the assay a histone representative, H3, as a negative control and a well-characterized ribosomal protein, RPL10 (AT1G14320), as a positive control. To assay for DNA binding activity, the recombinant HA-fused proteins were transcribed and translated *in vitro* (input) and incubated with ssDNA or dsDNA linked to sepharose beads. RNA binding activity was monitored by incubating the *in vitro* translated proteins with *A*. *thaliana* biotinylated RNA conjugated with streptavidin beads. As expected, the histone H3 bound to dsDNA and ssDNA, but not to RNA. In contrast, AT1G14320 (the L10 positive control) and the new predicted RPs, AT3G51010 and AT4G11385, bound to RNA with high affinity, bound to ssDNA with low affinity, and did not interact with dsDNA. These results confirmed that the new predicted RPs display a profile of nucleic acid binding activity which is consistent with the role of ribosomal proteins. Furthermore, they demonstrated that the ribosomal proteins bind to ssDNA although with a much lower affinity than histones. These *in vitro* results show the importance of the second stage of the Rama pipeline to distinguish ribosomal proteins from histones, since they share biochemical properties, such as ssDNA binding activity. It is important to highlight that InterProScan could not predict the proteins AT3G51010 and AT4G11385 as RPs.

## Conclusions

This study describes a new method to predict RPs called Rama. Our approach applies several ML models to predict RPs in two distinct steps. In the first step, RPs are distinguished from NRPs, while in the second step, RPs are distinguished from HPs. The ML models were constructed with sequences of five plant species and one phytoplankton species, using mostly Multilayer Perceptron and Random Forest.

The *in silico* experiments showed that Rama was able to differentiate RPs from other types of proteins for the six species with high success rate. Additionally to the positive *in silico* results, Rama was able to successfully predict two ribosomal proteins of *A*. *thaliana*, whose annotations were previously tagged as unknown function, that were experimentally confirmed *in vitro*. This experiment demonstrated that the two predicted proteins exhibited biochemical activity of ribosomal proteins as they strongly bound to RNA but not to double-stranded DNA (dsDNA). These results also confirmed the efficiency of our models to distinguish RPs from histones, which strongly bind to dsDNA. Interestingly, using the same sequences as input to InterProScan, this software could not find any evidence of their association with ribosomes. Additionally, Rama could perform its predictions approximately 600 times faster than InterProScan. This huge superiority in running time is expected, as Rama accesses no external databases or any other type of outer resources.

Although our experiments included six species, the inter-species tests provide evidence that our models might be used to other plant species as well. This argument was further substantiated by the successful performance of Rama using *O*. *lucimarinus* sequences, as this species is phylogenetically distant from the other considered species. In general, the consistency of the results obtained for one species using models from distinct species may be a consequence of using classification attributes that are conserved among proteins of any species, resulting in reliable ML models as predictors.

As a future effort, even though our set of attributes was proven appropriate, we intend to explore alternative classification attributes, such as reduced amino acid alphabets^21,22^. Another alternative would be considering the sequence order effect using attributes based on pseudo-amino acid composition, as proposed by Chou^23,24^. We also plan to study the viability of constructing an additional ML model for predicting the localization of the classified RPs.

The consistent results presented here using Rama, which applies a quite different approach as compared with sequence-homology procedures widely employed in the scientific community, further substantiate the notion that the method hereby described is promising and innovative, mainly as an important supplement to other known methods. Although largely-used computational tools, such as InterProScan, are available, it is interesting to explore alternative options based on different approaches to perform analyses far beyond the traditional procedures and to serve as an important complement for protein annotation.

## Materials and Methods

In the following sections, we describe the methodology with which several ML models used in conjunction were created with different algorithms and training datasets to make up the Rama method. Rama comprises two steps illustrated in Fig. 2, whose details are given below.

### Training sets

To create the training datasets, several protein sequences (and their annotations) from five plant species and one phytoplankton were acquired from the Phytozome v.10 database. The plant species were two monocotyledonous species, *O*. *sativa* and *Z*. *mays*, and three dicotyledonous species, *G*. *max*, *A*. *thaliana*, and *S*. *lycopersicum*, while the phytoplankton species was *O*. *lucimarinus*. The selected plant species have been extensively studied. The phytoplankton, in turn, which is phylogenetically distant from the selected plant species, was chosen to check whether an ML model constructed with its sequences could be successfully applied on distant plant species. Overall, thousands of proteins (amino acid sequences) were obtained: 35,386 of *A*. *thaliana*, 88,647 of *G*. *max*, 49,061 of *O*. *sativa*, 34,727 of *S*. *lycopersicum*, 88,760 of *Z*. *mays*, and 7,796 of *O*. *lucimarinus*.

After data acquisition, filters were applied to remove unknown proteins with no functional annotation, and proteins that have not been characterized. After the filter step, two TSs were generated for each species, one containing RPs (positive class) and NRPs (negative class), and the other containing RPs (positive class) and HPs (negative class). As expected, the resulting TSs of RPs/NRPs were extremely imbalanced, i.e., the number of NRPs was much greater than the number of RPs. Imbalanced TSs can result in models biased to the majority class, i.e., instances pertaining to the minority classes, which are often the ones of most interest, might be mostly or totally misclassified. For this reason, we used sampling with replacement to reduce the high number of negative examples. As a result, the final number of NRPs was three times greater than the number of RPs. According to our tests (not shown), this proportion was enough to avoid the bias problem, while not losing important information in the negative class. The TSs made of RPs and HPs did not present imbalance issues because the number of HPs is also reduced. The number of proteins used in the RPs/NRPs and RPs/HPs training sets is presented in Tables 6 and 7.

**Table 6:** Training set composed of RPs and NRPs. Number of RPs and NRPs that make up the training set for each species.

Training set	Number of RPs	Number of NRPs
*A*. *thaliana*	516	1548
*G*. *max*	1085	3255
*O*. *sativa*	539	1617
*O*. *lucimarinus*	176	528
*S*. *lycopersicum*	485	1455
*Z*. *mays*	1408	4224

**Table 7:** Training set composed of RPs and HPs. Number of RPs and HPs that make up the training set for each species.

Training set	Number of RPs	Number of HPs
*A*. *thaliana*	516	182
*G*. *max*	1085	269
*O*. *sativa*	539	112
*O*. *lucimarinus*	176	46
*S*. *lycopersicum*	485	119
*Z*. *mays*	1408	395

Notice that the positive examples of each species are comprised of sequences obtained from all its annotated loci. Furthermore, the negative examples were randomly selected in the sampling process. Extracting positive and negative sequences this way ensures the diversity of sequences in the TSs, which helps the construction of general models. To further demonstrate this diversity, the sequence identity was checked and analyzed with the program CD-HIT^24,25^. The results for all TSs are shown in Supplementary Figure S7 and Supplementary Figure S8 indicating that the number of sequences with low identity is dominant, in general.

Using this approach, 12 TSs, which include one dataset of RPs/NRPs and one dataset of RPs/HPs for each of the six species, were generated for building the models of our two-stages classification procedure. These TSs are available at http://inctipp.bioagro.ufv.br:8080/Rama/about.jsp. The files include the identification of the proteins used to compose the datasets for each species.

### Attribute extraction

The nine protein attributes used in our study are originated from the chemical properties of amino acid side chains, such as polarity, net charge, etc.^26^, conserved in all organisms. This approach allows correlating chemical properties of proteins with their functions. For example, ribosomal proteins are positively charged, which is a hallmark of proteins or functional domains that bind to nucleic acids.

Table 8 provides the complete description of all nine classification attributes used in our ML models. Five attributes are proportions of specific amino acids in the sequence. The attribute representing the proportion of aromatic amino acids, for example, is calculated by summing the number of amino acids F, Y, and W in the sequence, and dividing the result by the sequence length. Additionally to these five proportions, three attributes representing averages are also considered. Hidrophobicity, for example, is the average hydropathy index of amino acids in the sequence. Finally, in addition to these eight attributes, the sequence length is also part of the set of attributes.

**Table 8:** Description of the classification attributes and the type of amino acids used to calculate them.

Attribute	Attribute description	Amino acid types used to calculate the attribute value
Aromatic	Proportion of aromatic amino acids	F, Y, and W
Negatively charged	Proportion of negatively-charged amino acids	D and E
Nonpolar aliphatic	Proportion of nonpolar-aliphatic amino acids	G, A, P, V, L, I, and M
Polar uncharged	Proportion of polar-uncharged amino acids	S, T, C, N, and Q
Positively charged	Proportion of positively-charged amino acids	K, H, and R
Hydrophobicity	Average hydropathy index of amino acids in the sequence	All 20 amino acids
Molecular mass	Average mass of amino acids in the sequence	All 20 amino acids
Volume	Average volume of amino acids in the sequence	All 20 amino acids
Length	Total number of amino acids in the sequence	All 20 amino acids

Consider the sequence below for an example of attribute extraction:

MGKVHGSLARAGKVRGQTPKVAKQEKKKQPKGRAFQRIKYNRRFVNVVVGIGKKRSPNSNQA

In this case, the following attribute values are obtained: Aromatic = 0.048387, Negatively charged = 0.016129, Nonpolar aliphatic = 0.419355, Polar uncharged = 0.209677, Positively charged = 0.306452, Hidrophobicity = −1.062903, Molecular mass = 129.112903, Volume = 134.462903, Length = 62. The program in the Java programming language that performs these calculations is available at http://inctipp.bioagro.ufv.br:8080/Rama/about.jsp.

### Attribute evaluation

To measure the importance of each attribute used in RP prediction, we used Information Gain (IG)^27^. This method assesses an attribute (*attrib*) by measuring the gain in information regarding the class, and is defined by Equation 1.

1 $$I{G}_{attrib}=Entropy(class)-Entropy(class|attrib).$$

Entropy regarding the class is given by Equation 2, where *p*_*i*_ is the probability of class *i*. Notice that *Entropy*(*class*|*attrib*) is the weighted average entropy of all partitions (of the original data) derived from all possible values (or ranges) considered for the given attribute. The weight of a partition is a result of the number of instances in this partition divided by the number of instances in the original data. Therefore, the best attributes will be the ones leading to the more pronounced declines of entropy regarding the class.

2 $$Entropy(class)=-\sum {p}_{i}{\mathrm{log}}_{2}{p}_{i}$$

As a complementary attribute analysis, we produced density plots of the attribute values to visualize their distribution in each class. If a given attribute displays different distributions over the three classes (RP, NRP, and HP), it is an evidence that such an attribute is important for distinguishing instances of distinct classes. All plots were generated with the ggplot2 package in the software R v3.4.2 (www.cran.r-project.org), and can be seen in Supplementary Figures S1, S2, S3, S4, S5, and S6.

We did not perform additional attribute evaluations with other methods, such as PCA^28^, because our set of attributes is already small, i.e., this is not the case of high dimensionality issues. Furthermore, the IG analysis and the density plots clearly demonstrate that all nine classification attributes chosen in this work present some degree of relevance.

### Machine learning algorithms

To create models capable of predicting RPs for the chosen species in this study, we tested widely applied learning algorithms, including Multilayer Perceptron (MLP), Random Forest (RF), Sequential Minimal Optimization (SMO), LogitBoost, J48 Decision Tree, and Naive Bayes (NB). These algorithms are implemented in the software Weka v3.7.11^29^, whose API is used in this work.

MLP is likely the most commonly used architecture of the so-called artificial neural network approach^15^. MLP features three types of layers containing artificial neurons. The input layer has one neuron for each attribute. This layer receives a vector of attribute values to be computed by the network. After computation, the prediction produced by the MLP becomes available in the output layer. In a binary classification, this layer may have one or two neurons. Between the input and output layers, there are one or more intermediate layers containing one or more neurons. The links between the neurons do not form cycles, i.e., each neuron in the input layer and in the intermediate layers can be connected only to the neurons in the layer immediately ahead (feedforward connections). Those connections have associated weights that are determined in the learning process and define the final value yielded by the network for a vector of attribute values provided as input. To establish the connection weights, the most popular learning method used for MLPs is called backpropagation. In this case, the difference found between the network output and the value observed in the training set for a given training instance is used in the Gradient Descent algorithm to change the weights of connections in the opposite direction as the network computation, i.e., from the output layer to the input layer^15,30^.

The RF method is a type of ensemble approach, i.e., the final classification of an instance is performed from the majority vote of several models^31^. In the case of RF, the models are decision trees, each one built from a sample of the original training set. Each sample is generated taking a subset of the attributes. RFs have been widely used in recent bioinformatics research with satisfactory results^32,33^.

SMO is a procedure to solve the optimization problem resultant from the training process in the SVM approach^16^. The SVM method aims to find a maximum margin hyperplane so as to minimize overfitting. This hyperplane is defined from the solution of a quadratic optimization problem. When the data are not linearly separable, a kernel function is used to perform an implicit transformation of the space of attributes into a higher dimensional space in which the instances of different classes can be separated by a hyperplane^15^. In this work, we tested SMO in its linear version, i.e., without the use of kernel functions, and with the RBF Kernel function.

LogitBoost is a boosting-type ensemble algorithm. It can be derived by applying the cost function of logistic regression to the AdaBoost approach. In comparison with traditional AdaBoost, LogitBoost is less sensitive to noise and outliers^34^.

Decision trees, in general, are part of a broadly used and readily interpretable classification method, as the resultant rules that relate the attributes to the class can be extracted from the tree without major difficulties. The J48 algorithm used in this work is a Java implementation of the famous C4.5 algorithm. C4.5 is an evolution of a simpler approach called ID3. The proposal of C4.5 has brought the following improvements compared to simpler decision trees: inclusion of numerical attributes, treatment of missing values, and pruning after building the tree in order to decrease its complexity and minimize the possibility of overfitting^35,36^.

Naive Bayes provides a simple and quick approach based on the Bayes’ theorem. To estimate the probability of an instance attribute vector conditioned to the class (necessary to the class distribution calculation), it is assumed that the attributes are independent, which facilitates the calculations. If the independence assumption holds, or dependence is weak, NB results in accurate models^36,37^.

Those algorithms are used to create classification models from the training sets of RPs/NRPs and RPs/HPs. Therefore, when trained with RPs/NRPs, the learning models classify a protein as either ribosomal or non-ribosomal (step 1 of Rama), and when trained with RPs/HPs, they classify a protein as either ribosomal or histone (step 2 of Rama).

To choose the best ML approaches, different tests were performed using the datasets. For each algorithm, two types of computational experiment were carried out. The first one uses a single species, both for training and testing, and is comprised of two types of cross validation tests: a 10-fold cross validation and a jackknife (also called leave-one-out) test^17,18^. The second experiment mixes the six species, i.e., all permutations of the six datasets taken two datasets at a time (^6^ *P*_2_) are considered. For each permutation, a model is built with one species and then tested with the other species. Here, this type of test is referred to as inter-species test. All these training/test evaluation procedures were performed separately for RPs/NRPs and for RPs/HPs datasets.

As a result, we performed 84 tests (30 interspecies tests, six cross validations, and six jackknife tests for the six datasets of RPs/NRPs + 30 interspecies tests, six cross validations, and six jackknife tests for the six datasets of RPs/HPs) with each of the seven ML algorithms (SMO is counted twice: with and without kernel function) used in this work, totaling 588 model constructions. After all executions, the models that led to the best MCC in the inter-species and jackknife tests were chosen to compose the final pipeline.

Supplementary Table S3 shows all 588 tests made to select the best models. In the case of *A*. *thaliana*, for example, twelve models (six using RPs/NRPs, and six using RPs/HPs) were, at the end, selected to be included in the final pipeline according to each of the possible training/test scenarios: *A*. *thaliana*/*A*. *thaliana* (jackknife test); *G*. *max*/*A*. *thaliana*; *S*. *lycopersicum*/*A*. *thaliana*; *O*. *sativa*/*A*. *thaliana*; *Z*. *mays*/*A*. *thaliana*; *O*. *lucimarinus*/*A*. *thaliana*. The same was done for each of the other five species, leading to 72 models that were thus incorporated in Rama (Supplementary Tables S1 and S2).

### Prediction performance assessment

Three validation methods, i.e., independent dataset test (here termed inter-species), sub-sampling (or *k*-fold cross validation) test, and jackknife test, are often used to evaluate the success rate of a predictor. Evaluation using an independent dataset consists of one dataset to train and another to test. In *k*-fold cross validation the TS is partitioned into *k* equal sized sub-samples. A single subsample is retained as the validation data for testing the model, and the other *k*−1 sub-samples are used as training data. This process is repeated *k* times, each time using a different partition as test data. In the jackknife test, each sequence in the training dataset is in turn singled out as an independent test sample and the model parameters are calculated without including the test instance.

Among the three methods, however, the jackknife test is often the least arbitrary and most objective^18^, and hence has been widely used to examine the quality of various predictors^38–41^. Accordingly, the jackknife test was also used to examine the performance of the model proposed in the current study.

For each model evaluation, we considered the metrics used by Chou *et al*.^18^: sensitivity, specificity, accuracy, and Mathew’s correlation coefficient (MCC), in addition to F-measure and precision. These metrics are widely used in computational biology^42,43^. Equations 3–8 provide their definition, where TP, TN, FP, and FN represent the number of true positives, true negatives, false positives, and false negatives, respectively.

3 $$Precision=\frac{TP}{TP+FP}$$

4 $$Accuracy=\frac{TP+TN}{(TP+FN)+(FP+TN)}$$

5 $$Sensitivity\,or\,True\,Positive\,Rate\,(TPR)=\frac{TP}{TP+FN}$$

6 $$Specificity\,or\,True\,Negative\,Rate\,(TNR)=\frac{TN}{FP+TN}$$

7 $$F\mbox{--}measure=\frac{2TP}{2TP+FP+FN}$$

8 $$Matthews\,correlation\,coefficient\,(MCC)=\frac{TP\times TN-FP\times FN}{\sqrt{(TP+FP)\times (TP+FN)\times (TN+FP)\times (TN+FN)}}$$

### Ensemble approach

The inclusion of 72 ML models in Rama provides flexibility for choosing one of them or a set of models to work in an ensemble way. As illustrated in Fig. 2, step 1 classifies a protein using the models trained with the RPs/NRPs datasets. First in the system, the input sequences and the respective species have to be informed. Second, a set of species (training bases) has to be selected to indicate which ML models Rama has to load for performing the prediction task. For each input sequence, every loaded model calculates a probability of this sequence being an RP. Thereafter, the generated probabilities are averaged to produce the final ensemble probability *P*. The classification of the input sequence at this point will be determined according to the chosen discriminant probability (See Equation 9 and Fig. 2E). Considering 0.5 as the discriminant probability, for instance, if *P* < 0.5 (class 0), the amino acid sequence undergoes no further processing and is considered a non-ribosomal protein. However, if *P* ≥ 0.5 (class 1), the amino acid sequence is classified as a possible ribosomal protein and is given as input to step 2. The calculation that provides the sequence classification is given by Equation 9, where *n* is the number of applied models (default value: 6, i.e., five models built from inter-species tests and one model built from a jackknife test), *p*_*i*_ is the probability of being positive produced by model *i*, and *T* is the chosen discriminant probability (default value: 0.5).

9 $$Class=\{\begin{array}{c}1,\frac{{\sum }_{1}^{n}{p}_{i}}{n}\ge T\\ 0,\frac{{\sum }_{1}^{n}{p}_{i}}{n} < T\end{array}$$

Step 2 applies ML models to classify a sequence coming from step 1 as ribosomal or histone. This is necessary because RPs have some characteristics in common with HPs. For instance, both are positively charged proteins that interact with nucleic acids. RPs directly interact with RNAs that make up ribosomes, whereas HPs form histone-DNA complexes for the chromatin assembly. As described for the first step, in step 2 the applied ML models are those built for the selected species. However, in this case, Rama takes the models trained with datasets made up of RPs/HPs. In the same way, the generated probabilities are processed (averaged) to obtain the final classification (Fig. 2J). Thus, if the class is 0, the sequence is considered non-ribosomal. On the other hand, if the class is 1, it means evidence that the informed amino acid sequence corresponds to a ribosomal protein.

Notice that all *in silico* results shown in this work are from predictions using the default discriminant probability 0.5. However, if the goal is to obtain predictions with very high confidence, i.e., maximizing precision to save time and cost in laboratory validations, a higher probability threshold can be set.

### *In vitro* nucleic acid binding assay

The full-length candidate proteins AT3G51010 and AT4G11385 as well as the control proteins AT4G40030 (histone H3) and AT1G29970 (RPL18) were fused to HA tag and expressed *in vitro* using the TnT *in vitro* transcription/translation system (Promega). The DNA binding assay protocol was modified from Kaiserli *et al*.^44^. Equal amounts of protein were incubated with either single-stranded or double-stranded deoxyribonucleic acid lyophilized powder attached to cellulose beads from calf thymus DNA (1 mg/ml). After incubation at 4 °C for 30 min, the beads were washed five times in RHPA buffer (10 mM Tris-HCl, pH 7.4, 2.5 mM MgCl2, 0.5% (v/v) Triton X-100) and then boiled in SDS loading buffer. The proteins were separated by SDS-PAGE detected by western blot using an anti-HA antibody. The RNA binding assay was performed according to Vert and Chory^45^. Total RNA from *A*. *thaliana* were extracted and biotinylated using BrightStar Psolaren-Biotin kit, following the manufacture instructions. For RNA-protein pulldown, biotinylated RNAs were first incubated with streptavidin-bound beads (Dynabeads; invitrogen) in IP100 buffer (100 mM potassium glutamate, 50 mM Tris-HCl pH 7.5, 100 mM NaCl, 0.2% (v/v) Nonidet P40) for 2 h at 4 °C, then washed five times in IP100 buffer. Equal amounts of protein were then added to RNA-bound beads and incubated under rotation at 4 °C for 30 min. Subsequently, beads were washed five times with IP100 buffer, boiled in SDS loading buffer, and subjected to SDS-PAGE. The proteins were detected by western blot using an anti-HA antibody.

### Data availability

The protein sequences and respective annotations used to build the classification models for the current study are available in the Phytozome repository (releases 10 and 12): https://phytozome.jgi.doe.gov/pz/portal.html. These data were made available in FASTA and ARFF formats at http://inctipp.bioagro.ufv.br:8080/Rama/about.jsp.

## Electronic supplementary material

Supplementary Information
