# Supplementary material for: Rama: a machine learning approach for ribosomal protein prediction in plants
Source: Sci Rep. 2017 Nov 24;7:16273. doi: 10.1038/s41598-017-16322-4 (PMC5701237; doi:10.1038/s41598-017-16322-4)
Supplement: Supplementary file 1 — Supplementary Information [file 41598_2017_16322_MOESM1_ESM.pdf]

Rama: a machine learning approach for ribosomal protein prediction in plants

Supplementary Information

Thales Francisco Mota Carvalho[1†], José Cleydson F Silva[1,2, †], Iara Pinheiro Calil[2], Elizabeth Pacheco Batista Fontes[2&*], and Fabio Ribeiro Cerqueira[1,3&*]

[1] Computer Science Department, Universidade Federal de Viçosa, 36570-900, Minas Gerais, Brazil
[2] National Institute of Science and Technology in Plant-Pest Interactions/BIOAGRO, Universidade Federal de Viçosa, 36570-900, Minas Gerais, Brazil
[3] Department of Production Engineering, Universidade Federal Fluminense, Petrópolis, 25650-050, Rio de Janeiro, Brazil
[*] bbfontes@ufv.br and frcerqueira@id.uff.br
[†&] These authors contributed equally to this work

Contents

Supplementary Figure S1: Attribute density plots of *A. thaliana* datasets. 2
Supplementary Figure S2: Attribute density plots of *G. max* datasets. 3
Supplementary Figure S3: Attribute density plots of *O. lucimarinus* datasets. 4
Supplementary Figure S4: Attribute density plots of *O. sativa* datasets. 5
Supplementary Figure S5: Attribute density plots of *S. lycopersicum* datasets 6
Supplementary Figure S6: Attribute density plots of *Z. mays* datasets. 7
Supplementary Figure S7. Distribution of sequence identity in the RPs/NRPs datasets 8
Supplementary Figure S8. Distribution of sequence identity in the RPs/HPs datasets 9
Supplementary Table S1. Complete results of the classification models for the RPs/NRPs datasets. 10
Supplementary Table S2. Complete results of the classification models for the RPs/HPs datasets. 11
Supplementary Table S3. Algorithms tested to choose the classification models. 12
Supplementary Table S4. RNA-binding proteins tested in Rama. 26
Supplementary Table S5. Top 50 ranked protein sequences of *A. thaliana* with unknown function classified by Rama. 32

Supplementary Figure S1: Attribute density plots of *A. thaliana* datasets.

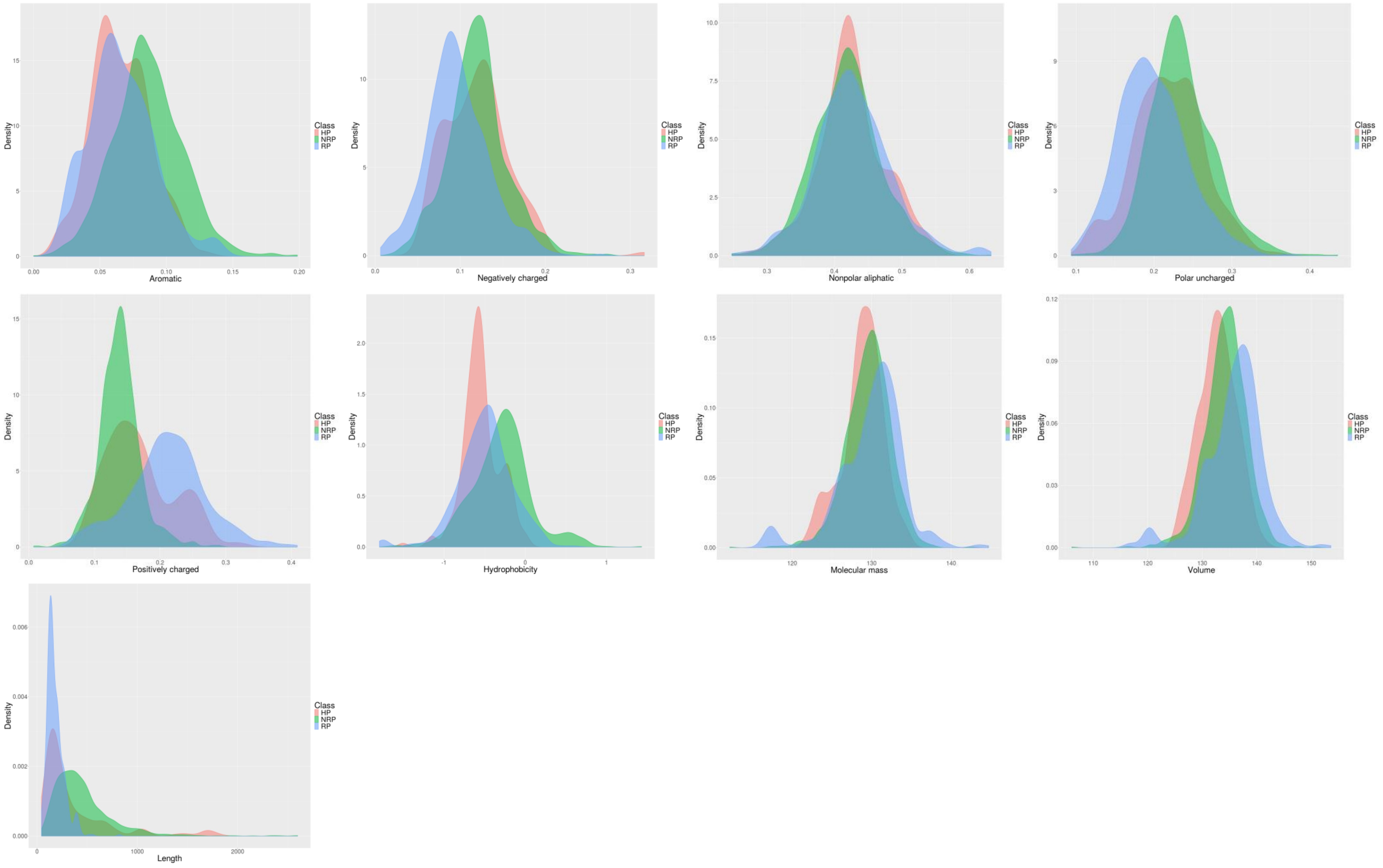

Supplementary Figure S2: Attribute density plots of *G. max* datasets.

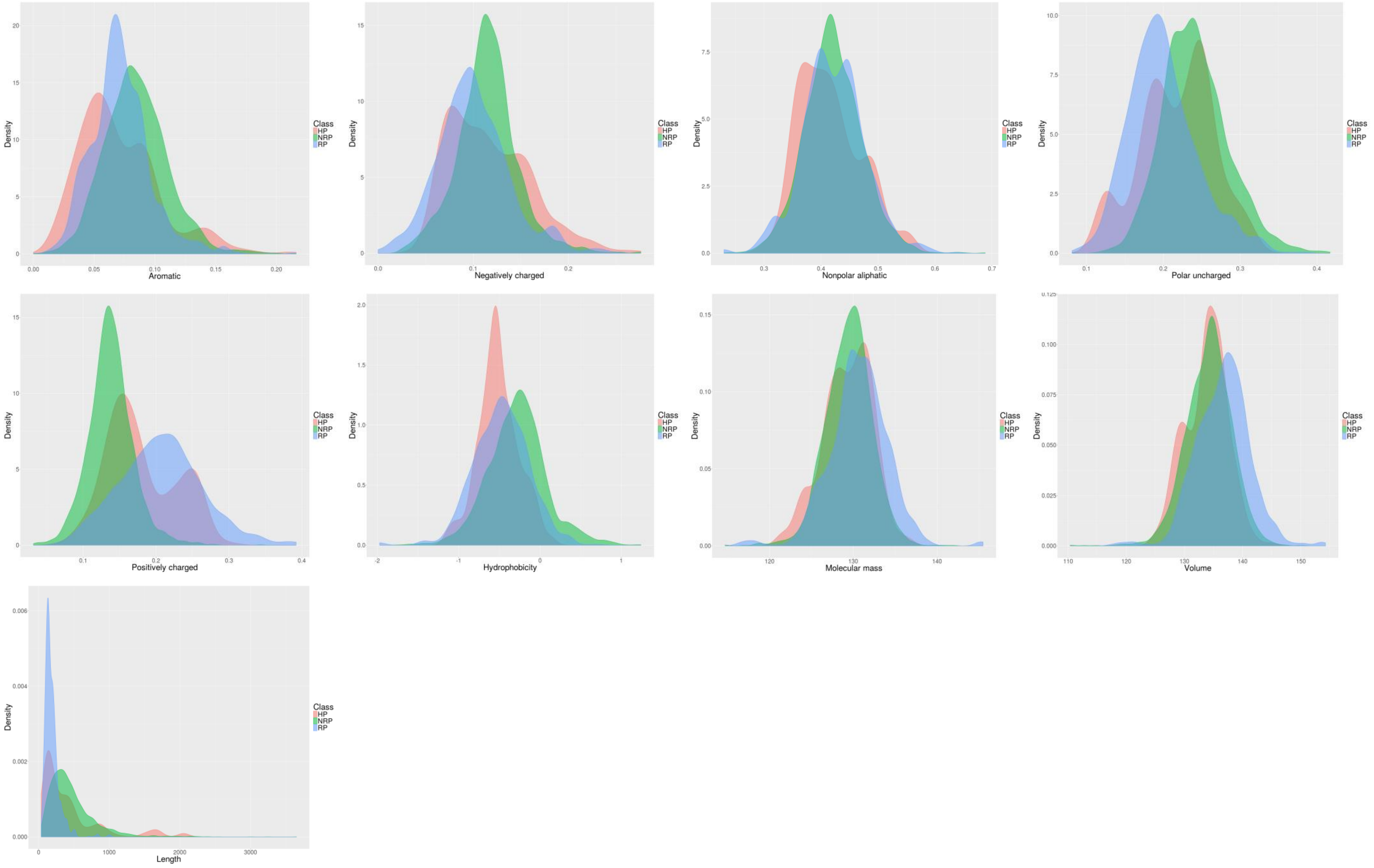

Supplementary Figure S3: Attribute density plots of *O. lucimarinus* datasets.

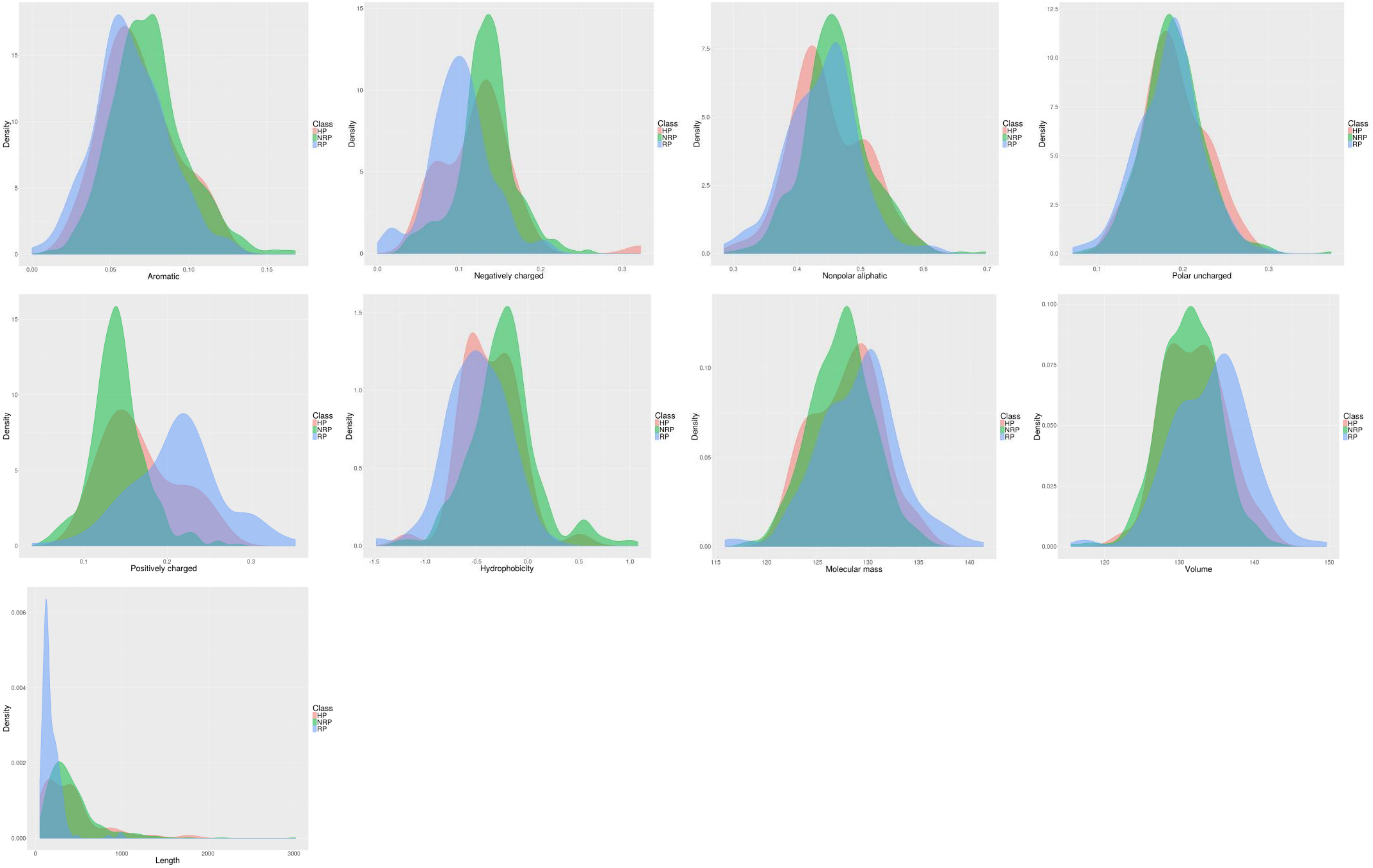

Supplementary Figure S4: Attribute density plots of *O. sativa* datasets.

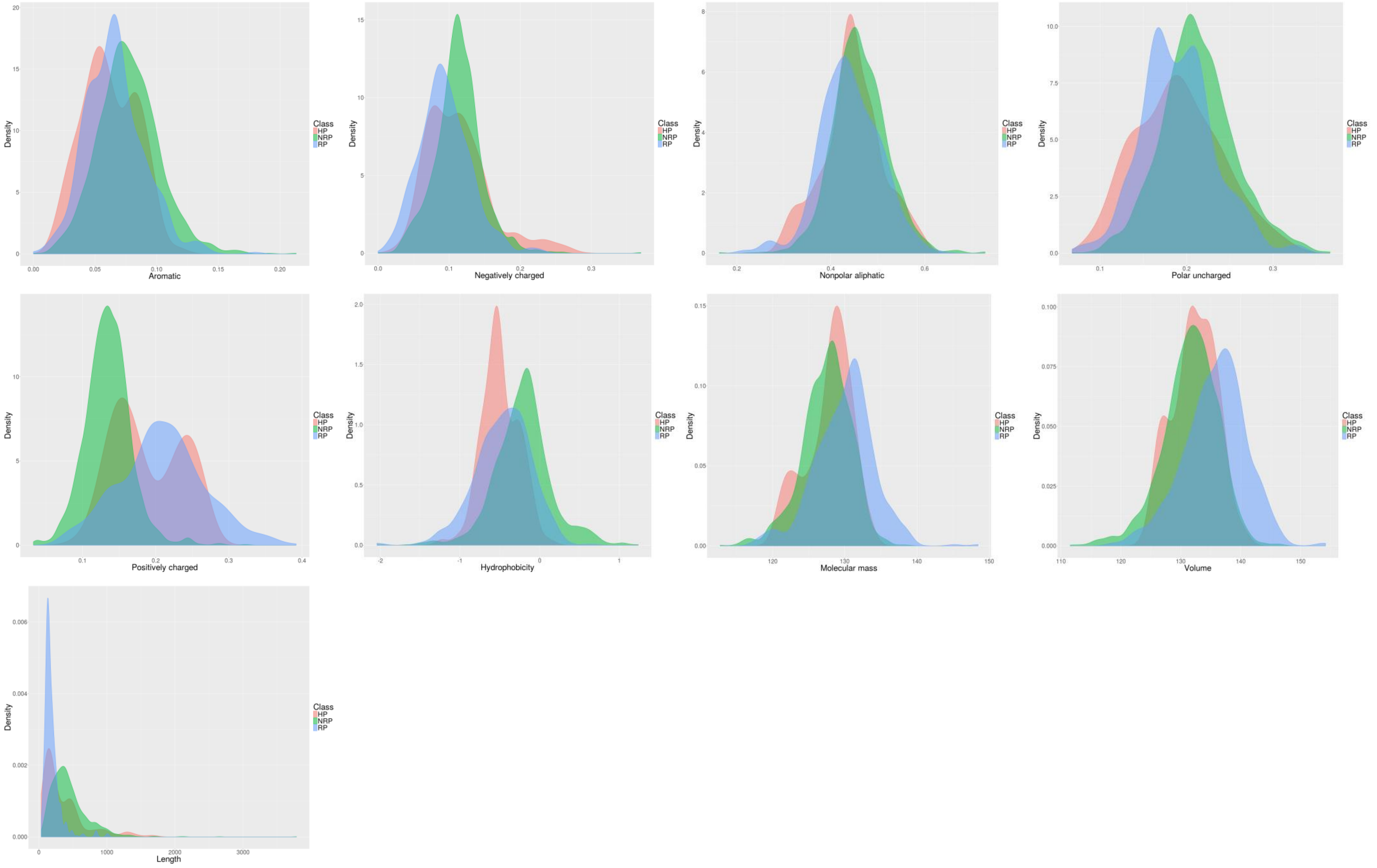

Supplementary Figure S5: Attribute density plots of *S. lycopersicum* datasets.

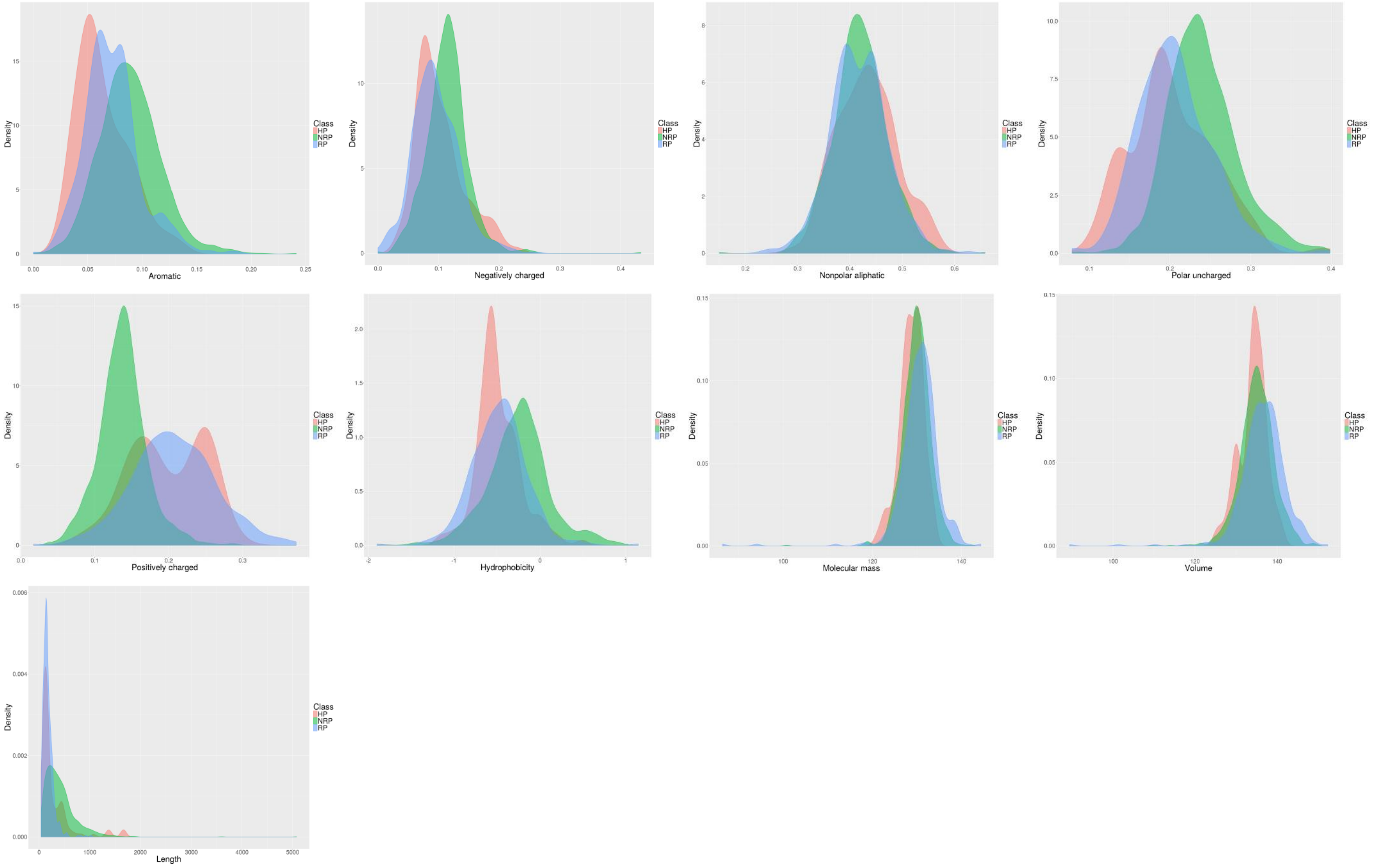

Supplementary Figure S6: Attribute density plots of *Z. mays* datasets.

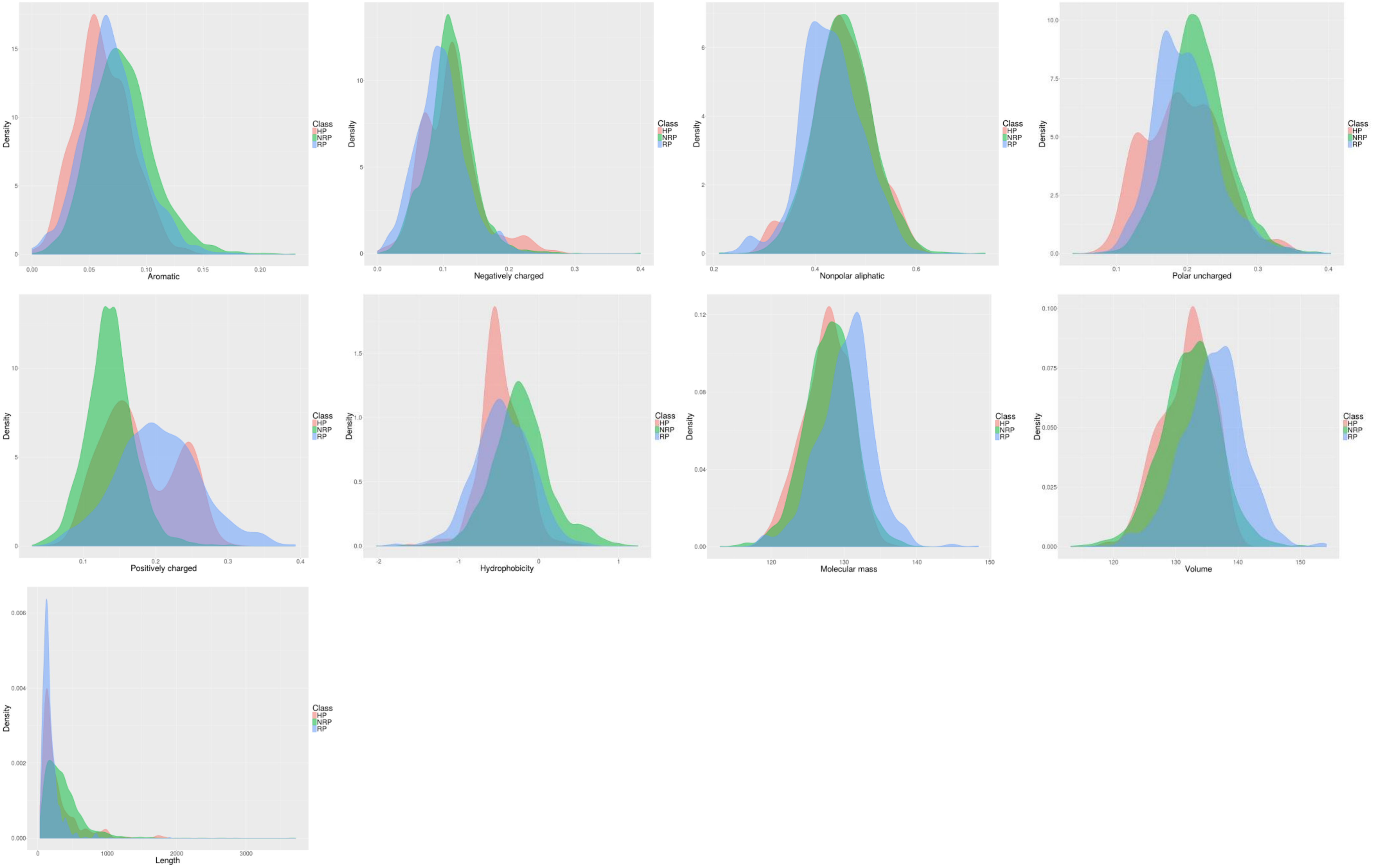

Supplementary Figure S7. Distribution of sequence identity in the RPs/NRPs datasets

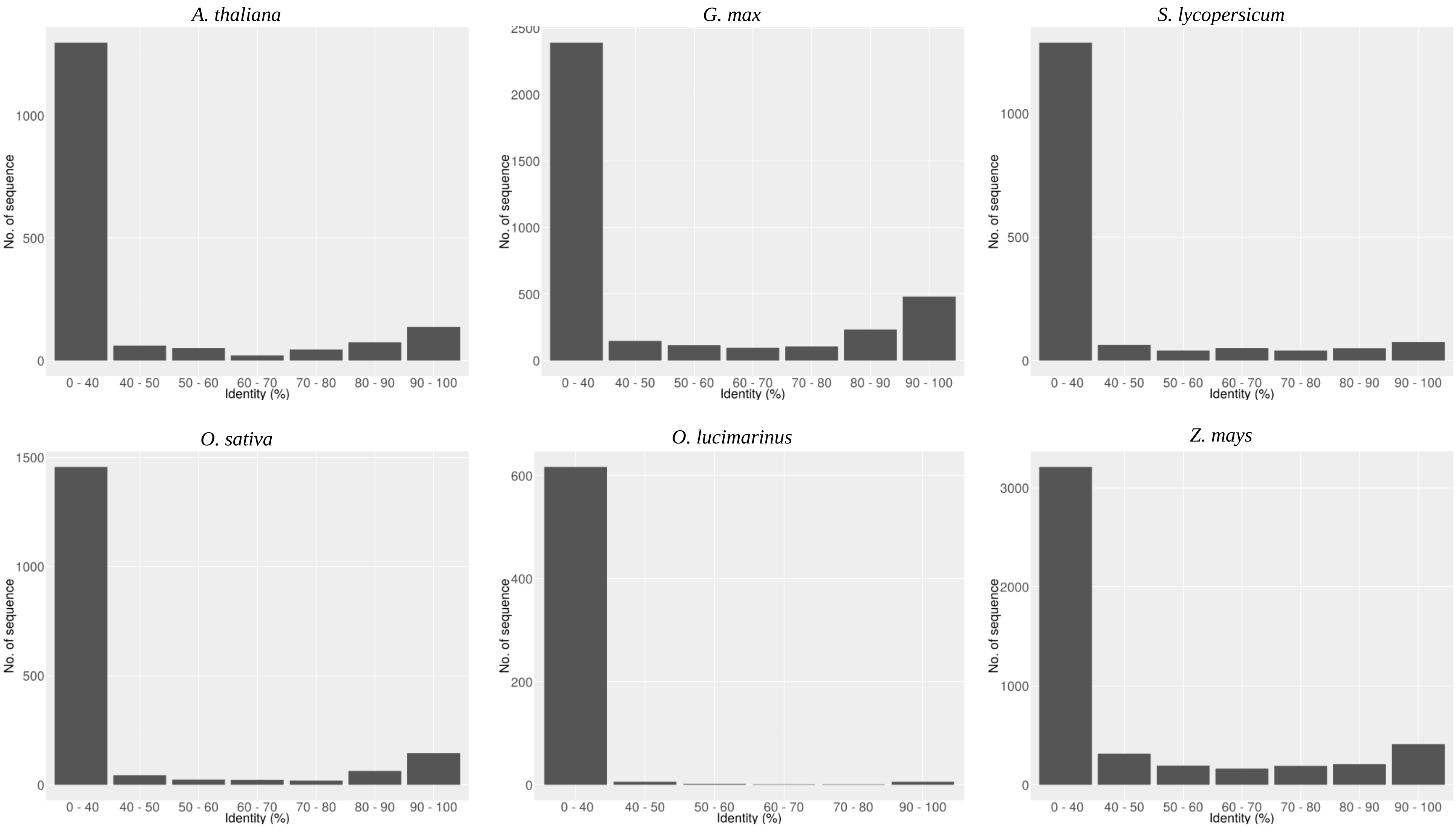

Supplementary Figure S8. Distribution of sequence identity in the RPs/HPs datasets

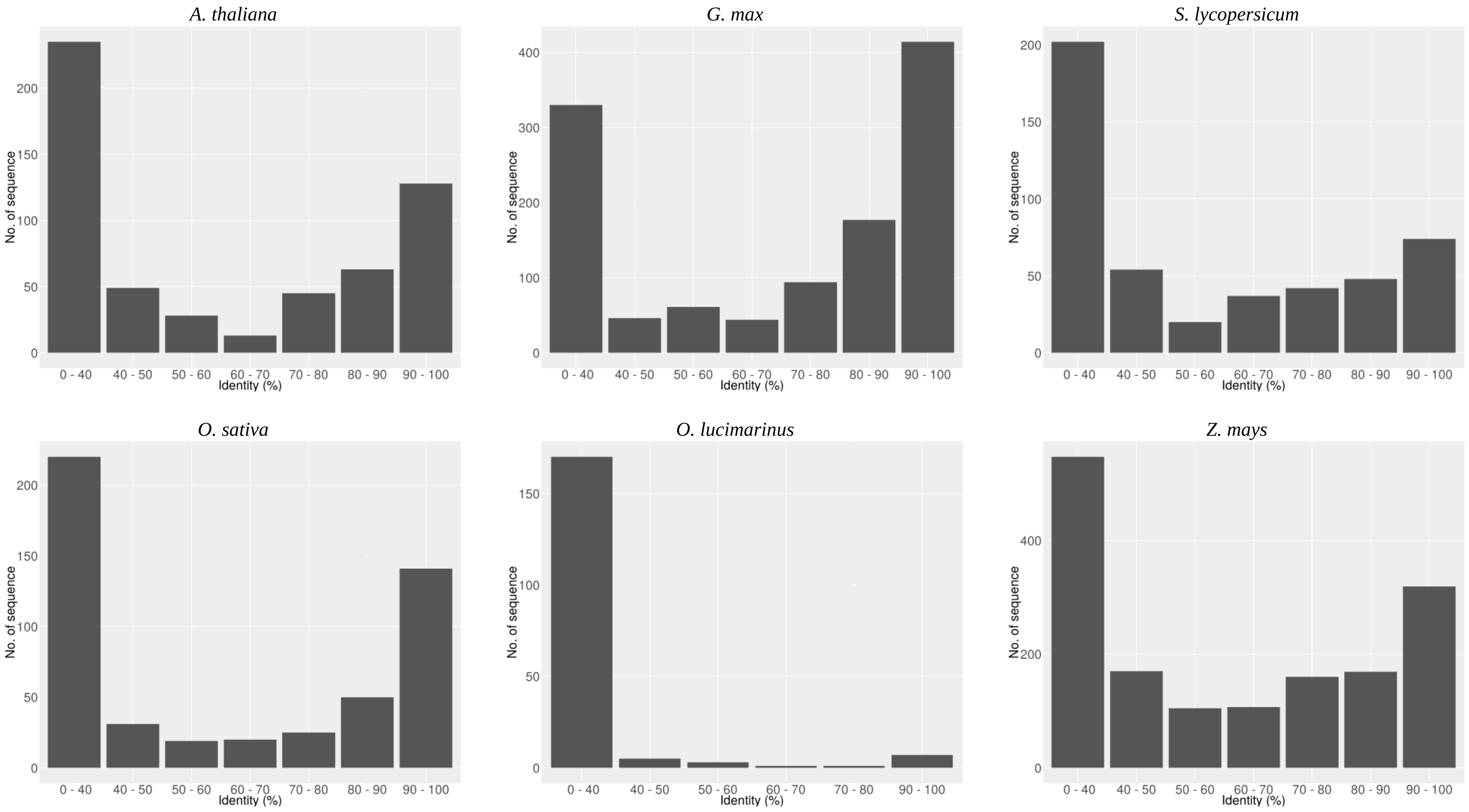

Supplementary Table S1. Complete results of the classification models for the RPs/NRPs datasets.

	Code	Training	Testing	Method	Accuracy	Sensitivity	Precision	F-measure	Specificity	MCC
Inter-species	M1	*Z. mays*	*A. thaliana*	RF	0.9273	0.9273	0.9272	0.9250	0.8233	0.8004
	M2	*S. lycopersicum*	*A. thaliana*	RF	0.9380	0.9380	0.9373	0.9369	0.8630	0.8309
	M3	*O. sativa*	*A. thaliana*	RF	0.9297	0.9297	0.9293	0.9279	0.8358	0.8074
	M4	*O. lucimarinus*	*A. thaliana*	MLP	0.9235	0.9234	0.9225	0.9228	0.8569	0.7930
	M5	*G. max*	*A. thaliana*	RF	0.9327	0.9327	0.9321	0.9310	0.8445	0.8156
	Mean				0.9302	0.9302	0.9297	0.9287	0.8447	0.8095
	M6	*Z. mays*	*G. max*	RF	0.9039	0.9039	0.9035	0.8995	0.7640	0.7327
	M7	*S. lycopersicum*	*G. max*	RF	0.9168	0.9168	0.9159	0.9142	0.8064	0.7705
	M8	*O. sativa*	*G. max*	RF	0.9090	0.9090	0.9082	0.9055	0.7829	0.7477
	M9	*O. lucimarinus*	*G. max*	MLP	0.8984	0.8984	0.8970	0.8975	0.8144	0.7249
	M10	*A. thaliana*	*G. max*	RF	0.9113	0.9113	0.9098	0.9088	0.8021	0.7552
	Mean				0.9079	0.9079	0.9069	0.9051	0.7940	0.7462
	M11	*Z. mays*	*O. lucimarinus*	MLP	0.9148	0.9148	0.9133	0.9126	0.8125	0.7654
	M12	*S. lycopersicum*	*O. lucimarinus*	MLP	0.9063	0.9062	0.9070	0.9066	0.8513	0.7520
	M13	*O. sativa*	*O. lucimarinus*	MLP	0.9105	0.9105	0.9111	0.9063	0.7732	0.7520
	M14	*G. max*	*O. lucimarinus*	RF	0.8793	0.8793	0.8840	0.8811	0.8310	0.6894
	M15	*A. thaliana*	*O. lucimarinus*	MLP	0.9034	0.9034	0.9042	0.9038	0.8466	0.7444
	Mean				0.9028	0.9028	0.9039	0.9021	0.8229	0.7406
	M16	*Z. mays*	*O. sativa*	RF	0.9253	0.9253	0.9247	0.9233	0.8267	0.7948
	M17	*S. lycopersicum*	*O. sativa*	RF	0.8947	0.8947	0.8958	0.8952	0.8338	0.7219
	M18	*O. lucimarinus*	*O. sativa*	MLP	0.9045	0.9045	0.9029	0.9034	0.8197	0.7404
	M19	*G. max*	*O. sativa*	RF	0.9058	0.9058	0.9080	0.9067	0.8622	0.7542
	M20	*A. thaliana*	*O. sativa*	MLP	0.9026	0.9026	0.9020	0.9023	0.8315	0.7387
	Mean				0.9066	0.9066	0.9067	0.9062	0.8348	0.7500
	M21	*Z. mays*	*S. lycopersicum*	RF	0.8974	0.8974	0.8953	0.8938	0.7679	0.7147
	M22	*O. sativa*	*S. lycopersicum*	RF	0.8912	0.8912	0.8886	0.8874	0.7576	0.6970
	M23	*O. lucimarinus*	*S. lycopersicum*	MLP	0.8933	0.8933	0.8931	0.8932	0.8201	0.7149
	M24	*G. max*	*S. lycopersicum*	RF	0.9129	0.9129	0.9114	0.9106	0.8074	0.7599
	M25	*A. thaliana*	*S. lycopersicum*	MLP	0.9057	0.9057	0.9037	0.9037	0.8050	0.7409
	Mean				0.9001	0.9001	0.8984	0.8977	0.7916	0.7255
	M26	*S. lycopersicum*	*Z. mays*	RF	0.8567	0.8567	0.8567	0.8567	0.7609	0.6178
	M27	*O. sativa*	*Z. mays*	RF	0.8817	0.8817	0.8783	0.8783	0.7513	0.6715
	M28	*O. lucimarinus*	*Z. mays*	MLP	0.8757	0.8757	0.8742	0.8748	0.7791	0.6642
	M29	*G. max*	*Z. mays*	RF	0.8619	0.8619	0.8605	0.8611	0.7589	0.6277
	M30	*A. thaliana*	*Z. mays*	RF	0.8565	0.8565	0.8581	0.8573	0.7713	0.6215
	Mean				0.8665	0.8665	0.8656	0.8656	0.7643	0.6405
Cross validation	M31	*Z. mays*		RF	0.9031	0.9031	0.9009	0.9007	0.7944	0.7327
	M32	*S. lycopersicum*		RF	0.9026	0.9026	0.9004	0.9001	0.7916	0.7311
	M33	*O. sativa*		RF	0.9249	0.9249	0.9239	0.9231	0.8327	0.7939
	M34	*O. lucimarinus*		MLP	0.9205	0.9205	0.9191	0.9190	0.8333	0.7823
	M35	*G. max*		RF	0.9438	0.9438	0.9432	0.9433	0.8909	0.8482
	M36	*A. thaliana*		RF	0.9428	0.9428	0.9422	0.9420	0.8776	0.8446
	Mean				0.9229	0.9230	0.9216	0.9214	0.8368	0.7888
Jackknife test	M31	*Z. mays*		RF	0.9069	0.9070	0.9051	0.9045	0.7981	0.7433
	M32	*S. lycopersicum*		RF	0.9077	0.9077	0.9059	0.9054	0.8015	0.7457
	M33	*O. sativa*		RF	0.9253	0.9253	0.9242	0.924	0.8428	0.7959
	M34	*O. lucimarinus*		RF	0.9148	0.9148	0.9133	0.9126	0.8125	0.7654
	M35	*G. max*		RF	0.9430	0.9431	0.9425	0.9426	0.8895	0.8462
	M36	*A. thaliana*		RF	0.9443	0.9443	0.9437	0.9435	0.8807	0.8486
	Mean				0.9237	0.9237	0.9225	0.9221	0.8375	0.7909

Inter-species and cross validation tests. The values are a weighted average of values for positive and negative examples. Notice that the values above are the measures obtained from the best algorithm after testing RF, MLP, SMO (with and without kernel function), LogitBoost, J48 and NB. The best ML algorithm is shown in the column ‘Method’.

Supplementary Table S2. Complete results of the classification models for the RPs/HPs datasets.

	Code	Training	Testing	Method	Accuracy	Sensitivity	Precision	F-measure	Specificity	MCC
Inter-species	M37	*Z. mays*	*A. thaliana*	RF	0.9226	0.9226	0.9259	0.9189	0.7984	0.7949
	M38	*S. lycopersicum*	*A. thaliana*	RF	0.8582	0.8582	0.8766	0.8391	0.6050	0.6156
	M39	*O. sativa*	*A. thaliana*	RF	0.8539	0.8539	0.8676	0.8351	0.6035	0.5991
	M40	*O. lucimarinus*	*A. thaliana*	RF	0.8381	0.8381	0.8429	0.8183	0.5837	0.5449
	M41	*G. max*	*A. thaliana*	RF	0.8940	0.8940	0.9000	0.8861	0.7208	0.7153
	Mean				0.8734	0.8734	0.8826	0.8595	0.6623	0.6540
	M42	*Z. mays*	*G. max*	RF	0.9114	0.9114	0.9093	0.9060	0.7096	0.7027
	M43	*S. lycopersicum*	*G. max*	RF	0.9158	0.9158	0.9200	0.9078	0.6772	0.7195
	M44	*O. sativa*	*G. max*	RF	0.8818	0.8818	0.8761	0.8720	0.6184	0.5921
	M45	*O. lucimarinus*	*G. max*	RF	0.8708	0.8708	0.8636	0.8652	0.6492	0.5666
	M46	*A. thaliana*	*G. max*	RF	0.9025	0.9025	0.9034	0.9029	0.7997	0.6964
	Mean				0.8965	0.8965	0.8945	0.8908	0.6908	0.6555
	M47	*Z. mays*	*O. lucimarinus*	RF	0.8694	0.8694	0.8627	0.8576	0.5965	0.5603
	M48	*S. lycopersicum*	*O. lucimarinus*	NB	0.8559	0.8559	0.8460	0.8418	0.5609	0.5088
	M49	*O. sativa*	*O. lucimarinus*	RF	0.8694	0.8694	0.8694	0.8516	0.5484	0.5556
	M50	*G. max*	*O. lucimarinus*	RF	0.8559	0.8559	0.8456	0.8438	0.5770	0.5131
	M51	*A. thaliana*	*O. lucimarinus*	RF	0.8604	0.8604	0.8640	0.8620	0.7387	0.5854
	Mean				0.8622	0.8622	0.8575	0.8514	0.6043	0.5446
	M52	*Z. mays*	*O. sativa*	RF	0.9432	0.9432	0.9417	0.9420	0.8184	0.7939
	M53	*S. lycopersicum*	*O. sativa*	RF	0.9155	0.9155	0.9121	0.9099	0.6783	0.6790
	M54	*O. lucimarinus*	*O. sativa*	RF	0.8833	0.8833	0.8746	0.8758	0.6009	0.5507
	M55	*G. max*	*O. sativa*	RF	0.9293	0.9293	0.9270	0.9259	0.7378	0.7364
	M56	*A. thaliana*	*O. sativa*	RF	0.9094	0.9094	0.9169	0.9121	0.8397	0.7052
	Mean				0.9161	0.9161	0.9145	0.9131	0.7350	0.6930
	M57	*Z. mays*	*S. lycopersicum*	RF	0.8891	0.8891	0.8884	0.8767	0.5986	0.6137
	M58	*O. sativa*	*S. lycopersicum*	RF	0.9023	0.9023	0.9003	0.8946	0.6653	0.6660
	M59	*O. lucimarinus*	*S. lycopersicum*	RF	0.8642	0.8642	0.8543	0.8532	0.5799	0.5239
	M60	*G. max*	*S. lycopersicum*	RF	0.9172	0.9172	0.9149	0.9132	0.7387	0.7229
	M61	*A. thaliana*	*S. lycopersicum*	RF	0.8758	0.8758	0.8709	0.8726	0.6842	0.5900
	Mean				0.8897	0.8897	0.8858	0.8821	0.6533	0.6233
	M62	*S. lycopersicum*	*Z. mays*	RF	0.8541	0.8541	0.8473	0.8387	0.5675	0.5247
	M63	*O. sativa*	*Z. mays*	RF	0.8569	0.8569	0.8529	0.8398	0.5591	0.5332
	M64	*O. lucimarinus*	*Z. mays*	RF	0.8148	0.8148	0.7952	0.7933	0.4817	0.3769
	M65	*G. max*	*Z. mays*	RF	0.8680	0.8680	0.8622	0.8575	0.6242	0.5786
	M66	*A. thaliana*	*Z. mays*	RF	0.8708	0.8708	0.8689	0.8697	0.7361	0.6166
	Mean				0.8529	0.8529	0.8453	0.8398	0.5937	0.5260
Cross validation	M67	*Z. mays*		RF	0.9185	0.9185	0.9169	0.9153	0.7749	0.7507
	M68	*S. lycopersicum*		RF	0.9139	0.9139	0.9156	0.9065	0.6808	0.7093
	M69	*O. sativa*		RF	0.9401	0.9401	0.9392	0.9370	0.7612	0.7779
	M70	*O. lucimarinus*		RF	0.8964	0.8964	0.8925	0.8931	0.7321	0.6694
	M71	*G. max*		RF	0.9616	0.9616	0.9614	0.9607	0.8759	0.8764
	M72	*A. thaliana*		RF	0.9484	0.9484	0.9485	0.9474	0.8822	0.8639
	Mean				0.9298	0.9298	0.9290	0.9267	0.7845	0.7746
Jackknife test	M67	*Z. mays*		RF	0.9212	0.9212	0.9198	0.9183	0.7830	0.7596
	M68	*S. lycopersicum*		RF	0.9156	0.9156	0.9171	0.9085	0.6876	0.7154
	M69	*O. sativa*		RF	0.9416	0.9416	0.9404	0.9390	0.7757	0.7843
	M70	*O. lucimarinus*		RF	0.9054	0.9054	0.9022	0.9024	0.7505	0.6986
	M71	*G. max*		RF	0.9616	0.9616	0.9616	0.9606	0.8703	0.8764
	M72	*A. thaliana*		RF	0.9456	0.9456	0.9457	0.9444	0.8741	0.8561
	Mean				0.9318	0.9318	0.9311	0.9289	0.7902	0.7817

Inter-species and cross validation tests. The values are a weighted average of values for positive and negative examples. Notice that the values above are the measures obtained from the best algorithm after testing RF, MLP, SMO (with and without kernel function), LogitBoost, J48, and NB. The best ML algorithm is shown in the column ‘Method’.

Supplementary Table S3. Algorithms tested to choose the classification models.

Data set	Code	Training	Testing	Accuracy	Sensitivity	Precision	F-Measure	Specificity	MCC	Weka algorithm
RPs / NRPs	M1	*Z. mays*	*A. thaliana*	0.8605	0.8605	0.8594	0.8599	0.7597	0.6251	Naive Bayes
RPs / NRPs	M1	*Z. mays*	*A. thaliana*	0.9162	0.9162	0.9147	0.9147	0.8286	0.7708	Multilayer Perceptron
RPs / NRPs	M1	*Z. mays*	*A. thaliana*	0.9041	0.9041	0.9042	0.8994	0.7600	0.7331	Sequential Minimal Optimization with PolyKernel
RPs / NRPs	M1	*Z. mays*	*A. thaliana*	0.8149	0.8149	0.8441	0.7727	0.4512	0.4524	Sequential Minimal Optimization with RBF kernel
RPs / NRPs	M1	*Z. mays*	*A. thaliana*	0.8924	0.8924	0.8910	0.8873	0.7419	0.6989	LogitBoost
RPs / NRPs	M1	*Z. mays*	*A. thaliana*	0.9070	0.9070	0.9062	0.9032	0.7765	0.7418	J48 Decision Tree
RPs / NRPs	M1	*Z. mays*	*A. thaliana*	0.9273	0.9273	0.9272	0.9250	0.8233	0.8004	Random Forest
RPs / NRPs	M2	*S. lycopersicum*	*A. thaliana*	0.8973	0.8973	0.8973	0.8973	0.8288	0.7261	Naive Bayes
RPs / NRPs	M2	*S. lycopersicum*	*A. thaliana*	0.9288	0.9288	0.9278	0.9275	0.8484	0.8055	Multilayer Perceptron
RPs / NRPs	M2	*S. lycopersicum*	*A. thaliana*	0.9089	0.9089	0.9082	0.9053	0.7810	0.7474	Sequential Minimal Optimization with PolyKernel
RPs / NRPs	M2	*S. lycopersicum*	*A. thaliana*	0.7505	0.7505	0.8128	0.6440	0.2515	0.0381	Sequential Minimal Optimization with RBF kernel
RPs / NRPs	M2	*S. lycopersicum*	*A. thaliana*	0.9065	0.9065	0.9060	0.9025	0.7724	0.7403	LogitBoost
RPs / NRPs	M2	*S. lycopersicum*	*A. thaliana*	0.9079	0.9079	0.9073	0.9076	0.8388	0.7527	J48 Decision Tree
RPs / NRPs	M2	*S. lycopersicum*	*A. thaliana*	0.9380	0.9380	0.9373	0.9369	0.8630	0.8309	Random Forest
RPs / NRPs	M3	*O. sativa*	*A. thaliana*	0.8658	0.8658	0.8650	0.8654	0.7705	0.6401	Naive Bayes
RPs / NRPs	M3	*O. sativa*	*A. thaliana*	0.9215	0.9215	0.9204	0.9196	0.8253	0.7844	Multilayer Perceptron
RPs / NRPs	M3	*O. sativa*	*A. thaliana*	0.9031	0.9031	0.9029	0.8984	0.7597	0.7303	Sequential Minimal Optimization with PolyKernel
RPs / NRPs	M3	*O. sativa*	*A. thaliana*	0.7626	0.7626	0.8197	0.6715	0.2878	0.1956	Sequential Minimal Optimization with RBF kernel
RPs / NRPs	M3	*O. sativa*	*A. thaliana*	0.8953	0.8953	0.8932	0.8915	0.7623	0.7086	LogitBoost
RPs / NRPs	M3	*O. sativa*	*A. thaliana*	0.9186	0.9186	0.9173	0.9166	0.8204	0.7763	J48 Decision Tree
RPs / NRPs	M3	*O. sativa*	*A. thaliana*	0.9297	0.9297	0.9293	0.9279	0.8358	0.8074	Random Forest
RPs / NRPs	M4	*O. lucimarinus*	*A. thaliana*	0.8522	0.8522	0.8576	0.8544	0.7854	0.6192	Naive Bayes
RPs / NRPs	M4	*O. lucimarinus*	*A. thaliana*	0.9234	0.9234	0.9225	0.9228	0.8569	0.7930	Multilayer Perceptron
RPs / NRPs	M4	*O. lucimarinus*	*A. thaliana*	0.9075	0.9075	0.9076	0.9031	0.7689	0.7431	Sequential Minimal Optimization with PolyKernel
RPs / NRPs	M4	*O. lucimarinus*	*A. thaliana*	0.7500	0.7500	0.5625	0.6429	0.2500	0.0000	Sequential Minimal Optimization with RBF kernel
RPs / NRPs	M4	*O. lucimarinus*	*A. thaliana*	0.8934	0.8934	0.8920	0.8884	0.7448	0.7019	LogitBoost
RPs / NRPs	M4	*O. lucimarinus*	*A. thaliana*	0.9002	0.9002	0.8979	0.8977	0.7884	0.7245	J48 Decision Tree
RPs / NRPs	M4	*O. lucimarinus*	*A. thaliana*	0.9191	0.9191	0.9182	0.9167	0.8128	0.7771	Random Forest
RPs / NRPs	M5	*G. max*	*A. thaliana*	0.8905	0.8905	0.8919	0.8911	0.8291	0.7116	Naive Bayes
RPs / NRPs	M5	*G. max*	*A. thaliana*	0.9249	0.9249	0.9238	0.9240	0.8509	0.7958	Multilayer Perceptron
RPs / NRPs	M5	*G. max*	*A. thaliana*	0.9128	0.9128	0.9117	0.9099	0.7978	0.7590	Sequential Minimal Optimization with PolyKernel
RPs / NRPs	M5	*G. max*	*A. thaliana*	0.8198	0.8198	0.8453	0.7812	0.4683	0.4692	Sequential Minimal Optimization with RBF kernel
RPs / NRPs	M5	*G. max*	*A. thaliana*	0.9002	0.9002	0.8983	0.8967	0.7742	0.7228	LogitBoost
RPs / NRPs	M5	*G. max*	*A. thaliana*	0.9104	0.9104	0.9091	0.9096	0.8332	0.7572	J48 Decision Tree
RPs / NRPs	M5	*G. max*	*A. thaliana*	0.9327	0.9327	0.9321	0.9310	0.8445	0.8156	Random Forest
RPs / NRPs	M6	*Z. mays*	*G. max*	0.8578	0.8578	0.8559	0.8567	0.7480	0.6154	Naive Bayes
RPs / NRPs	M6	*Z. mays*	*G. max*	0.8995	0.8995	0.8975	0.8979	0.8025	0.7252	Multilayer Perceptron
RPs / NRPs	M6	*Z. mays*	*G. max*	0.8926	0.8926	0.8936	0.8859	0.7233	0.6993	Sequential Minimal Optimization with PolyKernel
RPs / NRPs	M6	*Z. mays*	*G. max*	0.8120	0.8120	0.8435	0.7674	0.4409	0.4420	Sequential Minimal Optimization with RBF kernel
RPs / NRPs	M6	*Z. mays*	*G. max*	0.8806	0.8806	0.8789	0.8738	0.7095	0.6633	LogitBoost

RPs / NRPs	M6	*Z. mays*	*G. max*	0.8873	0.8873	0.8853	0.8819	0.7333	0.6839	J48 Decision Tree
RPs / NRPs	M6	*Z. mays*	*G. max*	0.9039	0.9039	0.9035	0.8995	0.7640	0.7327	Random Forest
RPs / NRPs	M7	*S. lycopersicum*	*G. max*	0.8816	0.8816	0.8789	0.8797	0.7737	0.6760	Naive Bayes
RPs / NRPs	M7	*S. lycopersicum*	*G. max*	0.9134	0.9134	0.9117	0.9115	0.8175	0.7622	Multilayer Perceptron
RPs / NRPs	M7	*S. lycopersicum*	*G. max*	0.8991	0.8991	0.8983	0.8943	0.7544	0.7185	Sequential Minimal Optimization with PolyKernel
RPs / NRPs	M7	*S. lycopersicum*	*G. max*	0.7500	0.7500	0.5625	0.6429	0.2500	0.0000	Sequential Minimal Optimization with RBF kernel
RPs / NRPs	M7	*S. lycopersicum*	*G. max*	0.8878	0.8878	0.8867	0.8816	0.7248	0.6848	LogitBoost
RPs / NRPs	M7	*S. lycopersicum*	*G. max*	0.8933	0.8933	0.8921	0.8926	0.8096	0.7121	J48 Decision Tree
RPs / NRPs	M7	*S. lycopersicum*	*G. max*	0.9168	0.9168	0.9159	0.9142	0.8064	0.7705	Random Forest
RPs / NRPs	M8	*O. sativa*	*G. max*	0.8654	0.8654	0.8639	0.8646	0.7634	0.6369	Naive Bayes
RPs / NRPs	M8	*O. sativa*	*G. max*	0.9018	0.9018	0.9010	0.8975	0.7627	0.7267	Multilayer Perceptron
RPs / NRPs	M8	*O. sativa*	*G. max*	0.8924	0.8924	0.8928	0.8860	0.7263	0.6985	Sequential Minimal Optimization with PolyKernel
RPs / NRPs	M8	*O. sativa*	*G. max*	0.7611	0.7611	0.8139	0.6685	0.2838	0.1820	Sequential Minimal Optimization with RBF kernel
RPs / NRPs	M8	*O. sativa*	*G. max*	0.8843	0.8843	0.8821	0.8786	0.7261	0.6749	LogitBoost
RPs / NRPs	M8	*O. sativa*	*G. max*	0.8896	0.8896	0.8867	0.8862	0.7617	0.6934	J48 Decision Tree
RPs / NRPs	M8	*O. sativa*	*G. max*	0.9090	0.9090	0.9082	0.9055	0.7829	0.7477	Random Forest
RPs / NRPs	M9	*O. lucimarinus*	*G. max*	0.8440	0.8440	0.8490	0.8461	0.7686	0.5965	Naive Bayes
RPs / NRPs	M9	*O. lucimarinus*	*G. max*	0.8984	0.8984	0.8970	0.8975	0.8144	0.7249	Multilayer Perceptron
RPs / NRPs	M9	*O. lucimarinus*	*G. max*	0.8873	0.8873	0.8866	0.8808	0.7204	0.6833	Sequential Minimal Optimization with PolyKernel
RPs / NRPs	M9	*O. lucimarinus*	*G. max*	0.7500	0.7500	0.5625	0.6429	0.2500	0.0000	Sequential Minimal Optimization with RBF kernel
RPs / NRPs	M9	*O. lucimarinus*	*G. max*	0.8871	0.8871	0.8860	0.8808	0.7233	0.6827	LogitBoost
RPs / NRPs	M9	*O. lucimarinus*	*G. max*	0.8783	0.8783	0.8749	0.8753	0.7530	0.6634	J48 Decision Tree
RPs / NRPs	M9	*O. lucimarinus*	*G. max*	0.8982	0.8982	0.8969	0.8937	0.7571	0.7160	Random Forest
RPs / NRPs	M10	*A. thaliana*	*G. max*	0.8869	0.8869	0.8845	0.8852	0.7841	0.6909	Naive Bayes
RPs / NRPs	M10	*A. thaliana*	*G. max*	0.9051	0.9051	0.9032	0.9023	0.7902	0.7375	Multilayer Perceptron
RPs / NRPs	M10	*A. thaliana*	*G. max*	0.9007	0.9007	0.8989	0.8971	0.7727	0.7240	Sequential Minimal Optimization with PolyKernel
RPs / NRPs	M10	*A. thaliana*	*G. max*	0.7590	0.7590	0.8116	0.6639	0.2776	0.1636	Sequential Minimal Optimization with RBF kernel
RPs / NRPs	M10	*A. thaliana*	*G. max*	0.8924	0.8924	0.8897	0.8899	0.7786	0.7032	LogitBoost
RPs / NRPs	M10	*A. thaliana*	*G. max*	0.8871	0.8871	0.8845	0.8852	0.7805	0.6906	J48 Decision Tree
RPs / NRPs	M10	*A. thaliana*	*G. max*	0.9113	0.9113	0.9098	0.9088	0.8021	0.7552	Random Forest
RPs / NRPs	M11	*Z. mays*	*O. lucimarinus*	0.8537	0.8537	0.8540	0.8538	0.7580	0.6106	Naive Bayes
RPs / NRPs	M11	*Z. mays*	*O. lucimarinus*	0.9148	0.9148	0.9133	0.9126	0.8125	0.7654	Multilayer Perceptron
RPs / NRPs	M11	*Z. mays*	*O. lucimarinus*	0.8878	0.8878	0.8878	0.8809	0.7164	0.6846	Sequential Minimal Optimization with PolyKernel
RPs / NRPs	M11	*Z. mays*	*O. lucimarinus*	0.7955	0.7955	0.8215	0.7413	0.3977	0.3702	Sequential Minimal Optimization with RBF kernel
RPs / NRPs	M11	*Z. mays*	*O. lucimarinus*	0.8722	0.8722	0.8680	0.8683	0.7339	0.6441	LogitBoost
RPs / NRPs	M11	*Z. mays*	*O. lucimarinus*	0.8665	0.8665	0.8620	0.8600	0.6979	0.6225	J48 Decision Tree
RPs / NRPs	M11	*Z. mays*	*O. lucimarinus*	0.9105	0.9105	0.9090	0.9079	0.7997	0.7529	Random Forest
RPs / NRPs	M12	*S. lycopersicum*	*O. lucimarinus*	0.8423	0.8423	0.8680	0.8490	0.8490	0.6348	Naive Bayes
RPs / NRPs	M12	*S. lycopersicum*	*O. lucimarinus*	0.9062	0.9062	0.9070	0.9066	0.8513	0.7520	Multilayer Perceptron
RPs / NRPs	M12	*S. lycopersicum*	*O. lucimarinus*	0.8821	0.8821	0.8844	0.8831	0.8205	0.6913	Sequential Minimal Optimization with PolyKernel
RPs / NRPs	M12	*S. lycopersicum*	*O. lucimarinus*	0.7500	0.7500	0.5625	0.6429	0.2500	0.0000	Sequential Minimal Optimization with RBF kernel
RPs / NRPs	M12	*S. lycopersicum*	*O. lucimarinus*	0.8722	0.8722	0.8694	0.8703	0.7604	0.6508	LogitBoost
RPs / NRPs	M12	*S. lycopersicum*	*O. lucimarinus*	0.8267	0.8267	0.8528	0.8339	0.8210	0.5954	J48 Decision Tree
RPs / NRPs	M12	*S. lycopersicum*	*O. lucimarinus*	0.8693	0.8693	0.8769	0.8720	0.8277	0.6692	Random Forest

RPs / NRPs	M13	*O. sativa*	*O. lucimarinus*	0.8580	0.8580	0.8597	0.8587	0.7746	0.6256	Naive Bayes
RPs / NRPs	M13	*O. sativa*	*O. lucimarinus*	0.9105	0.9105	0.9111	0.9063	0.7732	0.7520	Multilayer Perceptron
RPs / NRPs	M13	*O. sativa*	*O. lucimarinus*	0.8878	0.8878	0.8868	0.8815	0.7240	0.6847	Sequential Minimal Optimization with PolyKernel
RPs / NRPs	M13	*O. sativa*	*O. lucimarinus*	0.7614	0.7614	0.8190	0.6688	0.2841	0.1857	Sequential Minimal Optimization with RBF kernel
RPs / NRPs	M13	*O. sativa*	*O. lucimarinus*	0.8736	0.8736	0.8700	0.8708	0.7495	0.6512	LogitBoost
RPs / NRPs	M13	*O. sativa*	*O. lucimarinus*	0.8906	0.8906	0.8878	0.8880	0.7741	0.6980	J48 Decision Tree
RPs / NRPs	M13	*O. sativa*	*O. lucimarinus*	0.9020	0.9020	0.8998	0.8996	0.7931	0.7298	Random Forest
RPs / NRPs	M14	*G. max*	*O. lucimarinus*	0.8324	0.8324	0.8592	0.8395	0.8343	0.6115	Naive Bayes
RPs / NRPs	M14	*G. max*	*O. lucimarinus*	0.8466	0.8466	0.8682	0.8525	0.8428	0.6379	Multilayer Perceptron
RPs / NRPs	M14	*G. max*	*O. lucimarinus*	0.8778	0.8778	0.8823	0.8796	0.8267	0.6850	Sequential Minimal Optimization with PolyKernel
RPs / NRPs	M14	*G. max*	*O. lucimarinus*	0.8153	0.8153	0.8432	0.7737	0.4536	0.4534	Sequential Minimal Optimization with RBF kernel
RPs / NRPs	M14	*G. max*	*O. lucimarinus*	0.8452	0.8452	0.8566	0.8491	0.8007	0.6138	LogitBoost
RPs / NRPs	M14	*G. max*	*O. lucimarinus*	0.8438	0.8438	0.8624	0.8492	0.8267	0.6248	J48 Decision Tree
RPs / NRPs	M14	*G. max*	*O. lucimarinus*	0.8793	0.8793	0.8840	0.8811	0.8310	0.6894	Random Forest
RPs / NRPs	M15	*A. thaliana*	*O. lucimarinus*	0.8224	0.8224	0.8541	0.8306	0.8310	0.5951	Naive Bayes
RPs / NRPs	M15	*A. thaliana*	*O. lucimarinus*	0.9034	0.9034	0.9042	0.9038	0.8466	0.7444	Multilayer Perceptron
RPs / NRPs	M15	*A. thaliana*	*O. lucimarinus*	0.8608	0.8608	0.8695	0.8638	0.8172	0.6491	Sequential Minimal Optimization with PolyKernel
RPs / NRPs	M15	*A. thaliana*	*O. lucimarinus*	0.7585	0.7585	0.8173	0.6625	0.2756	0.1606	Sequential Minimal Optimization with RBF kernel
RPs / NRPs	M15	*A. thaliana*	*O. lucimarinus*	0.8722	0.8722	0.8804	0.8749	0.8362	0.6781	LogitBoost
RPs / NRPs	M15	*A. thaliana*	*O. lucimarinus*	0.8253	0.8253	0.8532	0.8328	0.8243	0.5953	J48 Decision Tree
RPs / NRPs	M15	*A. thaliana*	*O. lucimarinus*	0.8821	0.8821	0.8927	0.8853	0.8660	0.7090	Random Forest
RPs / NRPs	M16	*Z. mays*	*O. sativa*	0.8697	0.8697	0.8657	0.8665	0.7389	0.6392	Naive Bayes
RPs / NRPs	M16	*Z. mays*	*O. sativa*	0.9133	0.9133	0.9117	0.9111	0.8115	0.7613	Multilayer Perceptron
RPs / NRPs	M16	*Z. mays*	*O. sativa*	0.9063	0.9063	0.9067	0.9017	0.7635	0.7397	Sequential Minimal Optimization with PolyKernel
RPs / NRPs	M16	*Z. mays*	*O. sativa*	0.8117	0.8117	0.8448	0.7665	0.4388	0.4416	Sequential Minimal Optimization with RBF kernel
RPs / NRPs	M16	*Z. mays*	*O. sativa*	0.8901	0.8901	0.8886	0.8846	0.7358	0.6919	LogitBoost
RPs / NRPs	M16	*Z. mays*	*O. sativa*	0.8998	0.8998	0.8977	0.8965	0.7761	0.7220	J48 Decision Tree
RPs / NRPs	M16	*Z. mays*	*O. sativa*	0.9253	0.9253	0.9247	0.9233	0.8267	0.7948	Random Forest
RPs / NRPs	M17	*S. lycopersicum*	*O. sativa*	0.8469	0.8469	0.8620	0.8517	0.8191	0.6260	Naive Bayes
RPs / NRPs	M17	*S. lycopersicum*	*O. sativa*	0.8882	0.8882	0.8895	0.8888	0.8242	0.7052	Multilayer Perceptron
RPs / NRPs	M17	*S. lycopersicum*	*O. sativa*	0.8766	0.8766	0.8776	0.8771	0.8018	0.6735	Sequential Minimal Optimization with PolyKernel
RPs / NRPs	M17	*S. lycopersicum*	*O. sativa*	0.7500	0.7500	0.5625	0.6429	0.2500	0.0000	Sequential Minimal Optimization with RBF kernel
RPs / NRPs	M17	*S. lycopersicum*	*O. sativa*	0.8994	0.8994	0.8971	0.8963	0.7797	0.7212	LogitBoost
RPs / NRPs	M17	*S. lycopersicum*	*O. sativa*	0.8558	0.8558	0.8679	0.8597	0.8233	0.6430	J48 Decision Tree
RPs / NRPs	M17	*S. lycopersicum*	*O. sativa*	0.8947	0.8947	0.8958	0.8952	0.8338	0.7219	Random Forest
RPs / NRPs	M18	*O. lucimarinus*	*O. sativa*	0.8636	0.8636	0.8628	0.8632	0.7665	0.6341	Naive Bayes
RPs / NRPs	M18	*O. lucimarinus*	*O. sativa*	0.9045	0.9045	0.9029	0.9034	0.8197	0.7404	Multilayer Perceptron
RPs / NRPs	M18	*O. lucimarinus*	*O. sativa*	0.9040	0.9040	0.9019	0.9016	0.7948	0.7352	Sequential Minimal Optimization with PolyKernel
RPs / NRPs	M18	*O. lucimarinus*	*O. sativa*	0.7500	0.7500	0.5625	0.6429	0.2500	0.0000	Sequential Minimal Optimization with RBF kernel
RPs / NRPs	M18	*O. lucimarinus*	*O. sativa*	0.8878	0.8878	0.8847	0.8847	0.7647	0.6891	LogitBoost
RPs / NRPs	M18	*O. lucimarinus*	*O. sativa*	0.8780	0.8780	0.8750	0.8758	0.7639	0.6652	J48 Decision Tree
RPs / NRPs	M18	*O. lucimarinus*	*O. sativa*	0.8984	0.8984	0.8962	0.8965	0.7967	0.7214	Random Forest
RPs / NRPs	M19	*G. max*	*O. sativa*	0.8553	0.8553	0.8685	0.8594	0.8268	0.6439	Naive Bayes
RPs / NRPs	M19	*G. max*	*O. sativa*	0.8919	0.8919	0.8955	0.8933	0.8465	0.7202	Multilayer Perceptron

RPs / NRPs	M19	*G. max*	*O. sativa*	0.8827	0.8827	0.8840	0.8833	0.8149	0.6905	Sequential Minimal Optimization with PolyKernel
RPs / NRPs	M19	*G. max*	*O. sativa*	0.8159	0.8159	0.8390	0.7760	0.4600	0.4531	Sequential Minimal Optimization with RBF kernel
RPs / NRPs	M19	*G. max*	*O. sativa*	0.8720	0.8720	0.8725	0.8722	0.7904	0.6599	LogitBoost
RPs / NRPs	M19	*G. max*	*O. sativa*	0.8701	0.8701	0.8790	0.8731	0.8355	0.6741	J48 Decision Tree
RPs / NRPs	M19	*G. max*	*O. sativa*	0.9058	0.9058	0.9080	0.9067	0.8622	0.7542	Random Forest
RPs / NRPs	M20	*A. thaliana*	*O. sativa*	0.8353	0.8353	0.8586	0.8418	0.8276	0.6122	Naive Bayes
RPs / NRPs	M20	*A. thaliana*	*O. sativa*	0.9026	0.9026	0.9020	0.9023	0.8315	0.7387	Multilayer Perceptron
RPs / NRPs	M20	*A. thaliana*	*O. sativa*	0.8511	0.8511	0.8662	0.8557	0.8267	0.6368	Sequential Minimal Optimization with PolyKernel
RPs / NRPs	M20	*A. thaliana*	*O. sativa*	0.7583	0.7583	0.8172	0.6621	0.2750	0.1589	Sequential Minimal Optimization with RBF kernel
RPs / NRPs	M20	*A. thaliana*	*O. sativa*	0.8799	0.8799	0.8829	0.8811	0.8214	0.6871	LogitBoost
RPs / NRPs	M20	*A. thaliana*	*O. sativa*	0.8595	0.8595	0.8671	0.8622	0.8097	0.6433	J48 Decision Tree
RPs / NRPs	M20	*A. thaliana*	*O. sativa*	0.8966	0.8966	0.9008	0.8981	0.8592	0.7340	Random Forest
RPs / NRPs	M21	*Z. mays*	*S. lycopersicum*	0.8320	0.8320	0.8400	0.8351	0.7612	0.5714	Naive Bayes
RPs / NRPs	M21	*Z. mays*	*S. lycopersicum*	0.8835	0.8835	0.8837	0.8836	0.8072	0.6898	Multilayer Perceptron
RPs / NRPs	M21	*Z. mays*	*S. lycopersicum*	0.8809	0.8809	0.8788	0.8744	0.7143	0.6644	Sequential Minimal Optimization with PolyKernel
RPs / NRPs	M21	*Z. mays*	*S. lycopersicum*	0.8072	0.8072	0.8359	0.7605	0.4299	0.4213	Sequential Minimal Optimization with RBF kernel
RPs / NRPs	M21	*Z. mays*	*S. lycopersicum*	0.8711	0.8711	0.8674	0.8646	0.7027	0.6358	LogitBoost
RPs / NRPs	M21	*Z. mays*	*S. lycopersicum*	0.8644	0.8644	0.8596	0.8593	0.7101	0.6196	J48 Decision Tree
RPs / NRPs	M21	*Z. mays*	*S. lycopersicum*	0.8974	0.8974	0.8953	0.8938	0.7679	0.7147	Random Forest
RPs / NRPs	M22	*O. sativa*	*S. lycopersicum*	0.8412	0.8412	0.8469	0.8436	0.7670	0.5907	Naive Bayes
RPs / NRPs	M22	*O. sativa*	*S. lycopersicum*	0.8851	0.8851	0.8820	0.8825	0.7679	0.6830	Multilayer Perceptron
RPs / NRPs	M22	*O. sativa*	*S. lycopersicum*	0.8814	0.8814	0.8788	0.8757	0.7227	0.6665	Sequential Minimal Optimization with PolyKernel
RPs / NRPs	M22	*O. sativa*	*S. lycopersicum*	0.7608	0.7608	0.8187	0.6676	0.2825	0.1812	Sequential Minimal Optimization with RBF kernel
RPs / NRPs	M22	*O. sativa*	*S. lycopersicum*	0.8747	0.8747	0.8711	0.8691	0.7163	0.6475	LogitBoost
RPs / NRPs	M22	*O. sativa*	*S. lycopersicum*	0.8794	0.8794	0.8769	0.8777	0.7729	0.6707	J48 Decision Tree
RPs / NRPs	M22	*O. sativa*	*S. lycopersicum*	0.8912	0.8912	0.8886	0.8874	0.7576	0.6970	Random Forest
RPs / NRPs	M23	*O. lucimarinus*	*S. lycopersicum*	0.8103	0.8103	0.8338	0.8176	0.7814	0.5479	Naive Bayes
RPs / NRPs	M23	*O. lucimarinus*	*S. lycopersicum*	0.8933	0.8933	0.8931	0.8932	0.8201	0.7149	Multilayer Perceptron
RPs / NRPs	M23	*O. lucimarinus*	*S. lycopersicum*	0.8845	0.8845	0.8817	0.8797	0.7375	0.6766	Sequential Minimal Optimization with PolyKernel
RPs / NRPs	M23	*O. lucimarinus*	*S. lycopersicum*	0.7500	0.7500	0.5625	0.6429	0.2500	0.0000	Sequential Minimal Optimization with RBF kernel
RPs / NRPs	M23	*O. lucimarinus*	*S. lycopersicum*	0.8716	0.8716	0.8675	0.8663	0.7167	0.6394	LogitBoost
RPs / NRPs	M23	*O. lucimarinus*	*S. lycopersicum*	0.8680	0.8680	0.8656	0.8665	0.7581	0.6408	J48 Decision Tree
RPs / NRPs	M23	*O. lucimarinus*	*S. lycopersicum*	0.8881	0.8881	0.8856	0.8835	0.7442	0.6872	Random Forest
RPs / NRPs	M24	*G. max*	*S. lycopersicum*	0.8562	0.8562	0.8611	0.8582	0.7899	0.6285	Naive Bayes
RPs / NRPs	M24	*G. max*	*S. lycopersicum*	0.8928	0.8928	0.8907	0.8913	0.7952	0.7074	Multilayer Perceptron
RPs / NRPs	M24	*G. max*	*S. lycopersicum*	0.8969	0.8969	0.8949	0.8930	0.7636	0.7129	Sequential Minimal Optimization with PolyKernel
RPs / NRPs	M24	*G. max*	*S. lycopersicum*	0.8124	0.8124	0.8448	0.7678	0.4412	0.4440	Sequential Minimal Optimization with RBF kernel
RPs / NRPs	M24	*G. max*	*S. lycopersicum*	0.8768	0.8768	0.8730	0.8731	0.7418	0.6572	LogitBoost
RPs / NRPs	M24	*G. max*	*S. lycopersicum*	0.8809	0.8809	0.8780	0.8787	0.7679	0.6730	J48 Decision Tree
RPs / NRPs	M24	*G. max*	*S. lycopersicum*	0.9129	0.9129	0.9114	0.9106	0.8074	0.7599	Random Forest
RPs / NRPs	M25	*A. thaliana*	*S. lycopersicum*	0.8691	0.8691	0.8710	0.8699	0.7955	0.6558	Naive Bayes
RPs / NRPs	M25	*A. thaliana*	*S. lycopersicum*	0.9057	0.9057	0.9037	0.9037	0.8050	0.7409	Multilayer Perceptron
RPs / NRPs	M25	*A. thaliana*	*S. lycopersicum*	0.8933	0.8933	0.8909	0.8895	0.7610	0.7029	Sequential Minimal Optimization with PolyKernel
RPs / NRPs	M25	*A. thaliana*	*S. lycopersicum*	0.7582	0.7582	0.8172	0.6619	0.2747	0.1579	Sequential Minimal Optimization with RBF kernel

RPs / NRPs	M25	*A. thaliana*	*S. lycopersicum*	0.8835	0.8835	0.8813	0.8821	0.7825	0.6827	LogitBoost
RPs / NRPs	M25	*A. thaliana*	*S. lycopersicum*	0.8809	0.8809	0.8799	0.8804	0.7926	0.6797	J48 Decision Tree
RPs / NRPs	M25	*A. thaliana*	*S. lycopersicum*	0.9015	0.9015	0.8993	0.8992	0.7926	0.7286	Random Forest
RPs / NRPs	M26	*S. lycopersicum*	*Z. mays*	0.8118	0.8118	0.8269	0.8171	0.7536	0.5340	Naive Bayes
RPs / NRPs	M26	*S. lycopersicum*	*Z. mays*	0.8473	0.8473	0.8489	0.8480	0.7554	0.5969	Multilayer Perceptron
RPs / NRPs	M26	*S. lycopersicum*	*Z. mays*	0.8430	0.8430	0.8395	0.8409	0.7128	0.5712	Sequential Minimal Optimization with PolyKernel
RPs / NRPs	M26	*S. lycopersicum*	*Z. mays*	0.7500	0.7500	0.5625	0.6429	0.2500	0.0000	Sequential Minimal Optimization with RBF kernel
RPs / NRPs	M26	*S. lycopersicum*	*Z. mays*	0.8608	0.8608	0.8556	0.8555	0.7036	0.6090	LogitBoost
RPs / NRPs	M26	*S. lycopersicum*	*Z. mays*	0.8265	0.8265	0.8374	0.8305	0.7637	0.5634	J48 Decision Tree
RPs / NRPs	M26	*S. lycopersicum*	*Z. mays*	0.8567	0.8567	0.8567	0.8567	0.7609	0.6178	Random Forest
RPs / NRPs	M27	*O. sativa*	*Z. mays*	0.8414	0.8414	0.8407	0.8410	0.7308	0.5751	Naive Bayes
RPs / NRPs	M27	*O. sativa*	*Z. mays*	0.8732	0.8732	0.8692	0.8682	0.7219	0.6444	Multilayer Perceptron
RPs / NRPs	M27	*O. sativa*	*Z. mays*	0.8665	0.8665	0.8620	0.8600	0.6979	0.6225	Sequential Minimal Optimization with PolyKernel
RPs / NRPs	M27	*O. sativa*	*Z. mays*	0.7592	0.7592	0.8177	0.6641	0.2777	0.1672	Sequential Minimal Optimization with RBF kernel
RPs / NRPs	M27	*O. sativa*	*Z. mays*	0.8588	0.8588	0.8534	0.8532	0.6973	0.6027	LogitBoost
RPs / NRPs	M27	*O. sativa*	*Z. mays*	0.8659	0.8659	0.8630	0.8641	0.7503	0.6338	J48 Decision Tree
RPs / NRPs	M27	*O. sativa*	*Z. mays*	0.8817	0.8817	0.8783	0.8783	0.7513	0.6715	Random Forest
RPs / NRPs	M28	*O. lucimarinus*	*Z. mays*	0.8265	0.8265	0.8327	0.8291	0.7428	0.5528	Naive Bayes
RPs / NRPs	M28	*O. lucimarinus*	*Z. mays*	0.8757	0.8757	0.8742	0.8748	0.7791	0.6642	Multilayer Perceptron
RPs / NRPs	M28	*O. lucimarinus*	*Z. mays*	0.8603	0.8603	0.8550	0.8550	0.7029	0.6076	Sequential Minimal Optimization with PolyKernel
RPs / NRPs	M28	*O. lucimarinus*	*Z. mays*	0.7500	0.7500	0.5625	0.6429	0.2500	0.0000	Sequential Minimal Optimization with RBF kernel
RPs / NRPs	M28	*O. lucimarinus*	*Z. mays*	0.8509	0.8509	0.8452	0.8462	0.6965	0.5831	LogitBoost
RPs / NRPs	M28	*O. lucimarinus*	*Z. mays*	0.8491	0.8491	0.8463	0.8475	0.7281	0.5897	J48 Decision Tree
RPs / NRPs	M28	*O. lucimarinus*	*Z. mays*	0.8585	0.8585	0.8536	0.8545	0.7147	0.6063	Random Forest
RPs / NRPs	M29	*G. max*	*Z. mays*	0.8152	0.8152	0.8305	0.8205	0.7608	0.5434	Naive Bayes
RPs / NRPs	M29	*G. max*	*Z. mays*	0.8512	0.8512	0.8552	0.8529	0.7762	0.6133	Multilayer Perceptron
RPs / NRPs	M29	*G. max*	*Z. mays*	0.8494	0.8494	0.8472	0.8482	0.7330	0.5922	Sequential Minimal Optimization with PolyKernel
RPs / NRPs	M29	*G. max*	*Z. mays*	0.8088	0.8088	0.8263	0.7666	0.4457	0.4227	Sequential Minimal Optimization with RBF kernel
RPs / NRPs	M29	*G. max*	*Z. mays*	0.8317	0.8317	0.8317	0.8317	0.7195	0.5511	LogitBoost
RPs / NRPs	M29	*G. max*	*Z. mays*	0.8315	0.8315	0.8377	0.8341	0.7521	0.5661	J48 Decision Tree
RPs / NRPs	M29	*G. max*	*Z. mays*	0.8619	0.8619	0.8605	0.8611	0.7589	0.6277	Random Forest
RPs / NRPs	M30	*A. thaliana*	*Z. mays*	0.7956	0.7956	0.8196	0.8034	0.7557	0.5109	Naive Bayes
RPs / NRPs	M30	*A. thaliana*	*Z. mays*	0.8601	0.8601	0.8579	0.8588	0.7498	0.6208	Multilayer Perceptron
RPs / NRPs	M30	*A. thaliana*	*Z. mays*	0.8187	0.8187	0.8280	0.8223	0.7426	0.5393	Sequential Minimal Optimization with PolyKernel
RPs / NRPs	M30	*A. thaliana*	*Z. mays*	0.7553	0.7553	0.7802	0.6571	0.2688	0.1215	Sequential Minimal Optimization with RBF kernel
RPs / NRPs	M30	*A. thaliana*	*Z. mays*	0.8303	0.8303	0.8350	0.8323	0.7427	0.5593	LogitBoost
RPs / NRPs	M30	*A. thaliana*	*Z. mays*	0.8187	0.8187	0.8304	0.8231	0.7530	0.5448	J48 Decision Tree
RPs / NRPs	M30	*A. thaliana*	*Z. mays*	0.8565	0.8565	0.8581	0.8573	0.7713	0.6215	Random Forest
RPs / NRPs	M31	cross validation *Z. mays*		0.8382	0.8382	0.8359	0.8369	0.7145	0.5620	Naive Bayes
RPs / NRPs	M31	cross validation *Z. mays*		0.8839	0.8839	0.8807	0.8811	0.7629	0.6791	Multilayer Perceptron
RPs / NRPs	M31	cross validation *Z. mays*		0.8699	0.8699	0.8668	0.8621	0.6891	0.6307	Sequential Minimal Optimization with PolyKernel
RPs / NRPs	M31	cross validation *Z. mays*		0.7900	0.7900	0.8201	0.7303	0.3784	0.3467	Sequential Minimal Optimization with RBF kernel
RPs / NRPs	M31	cross validation *Z. mays*		0.8619	0.8619	0.8569	0.8550	0.6888	0.6087	LogitBoost
RPs / NRPs	M31	cross validation *Z. mays*		0.8750	0.8750	0.8711	0.8714	0.7410	0.6526	J48 Decision Tree

RPs / NRPs	M31	cross validation *Z. mays*	0.9031	0.9031	0.9009	0.9007	0.7944	0.7327	Random Forest
RPs / NRPs	M32	cross validation *S. lycopersicum*	0.8670	0.8670	0.8676	0.8673	0.7825	0.6468	Naive Bayes
RPs / NRPs	M32	cross validation *S. lycopersicum*	0.8861	0.8861	0.8840	0.8847	0.7875	0.6899	Multilayer Perceptron
RPs / NRPs	M32	cross validation *S. lycopersicum*	0.8933	0.8933	0.8919	0.8883	0.7445	0.7015	Sequential Minimal Optimization with PolyKernel
RPs / NRPs	M32	cross validation *S. lycopersicum*	0.7505	0.7505	0.8128	0.6441	0.2515	0.0393	Sequential Minimal Optimization with RBF kernel
RPs / NRPs	M32	cross validation *S. lycopersicum*	0.8753	0.8753	0.8715	0.8700	0.7220	0.6497	LogitBoost
RPs / NRPs	M32	cross validation *S. lycopersicum*	0.8737	0.8737	0.8709	0.8718	0.7613	0.6546	J48 Decision Tree
RPs / NRPs	M32	cross validation *S. lycopersicum*	0.9026	0.9026	0.9004	0.9001	0.7916	0.7311	Random Forest
RPs / NRPs	M33	cross validation *O. sativa*	0.8785	0.8785	0.8755	0.8764	0.7653	0.6666	Naive Bayes
RPs / NRPs	M33	cross validation *O. sativa*	0.9170	0.9170	0.9155	0.9155	0.8288	0.7729	Multilayer Perceptron
RPs / NRPs	M33	cross validation *O. sativa*	0.9040	0.9040	0.9029	0.9002	0.7726	0.7332	Sequential Minimal Optimization with PolyKernel
RPs / NRPs	M33	cross validation *O. sativa*	0.7542	0.7542	0.8149	0.6526	0.2625	0.1121	Sequential Minimal Optimization with RBF kernel
RPs / NRPs	M33	cross validation *O. sativa*	0.8891	0.8891	0.8864	0.8850	0.7515	0.6907	LogitBoost
RPs / NRPs	M33	cross validation *O. sativa*	0.8947	0.8947	0.8922	0.8925	0.7856	0.7102	J48 Decision Tree
RPs / NRPs	M33	cross validation *O. sativa*	0.9249	0.9249	0.9239	0.9231	0.8327	0.7939	Random Forest
RPs / NRPs	M34	cross validation *O. lucimarinus*	0.8636	0.8636	0.8647	0.8641	0.7803	0.6392	Naive Bayes
RPs / NRPs	M34	cross validation *O. lucimarinus*	0.9205	0.9205	0.9191	0.9190	0.8333	0.7823	Multilayer Perceptron
RPs / NRPs	M34	cross validation *O. lucimarinus*	0.8949	0.8949	0.8935	0.8901	0.7491	0.7063	Sequential Minimal Optimization with PolyKernel
RPs / NRPs	M34	cross validation *O. lucimarinus*	0.7500	0.7500	0.5625	0.6429	0.2500	0.0000	Sequential Minimal Optimization with RBF kernel
RPs / NRPs	M34	cross validation *O. lucimarinus*	0.8935	0.8935	0.8910	0.8914	0.7865	0.7073	LogitBoost
RPs / NRPs	M34	cross validation *O. lucimarinus*	0.8878	0.8878	0.8850	0.8856	0.7770	0.6915	J48 Decision Tree
RPs / NRPs	M34	cross validation *O. lucimarinus*	0.9077	0.9077	0.9063	0.9045	0.7874	0.7442	Random Forest
RPs / NRPs	M35	cross validation *G. max*	0.8770	0.8770	0.8754	0.8761	0.7808	0.6675	Naive Bayes
RPs / NRPs	M35	cross validation *G. max*	0.9076	0.9076	0.9061	0.9065	0.8236	0.7487	Multilayer Perceptron
RPs / NRPs	M35	cross validation *G. max*	0.8993	0.8993	0.8979	0.8951	0.7618	0.7195	Sequential Minimal Optimization with PolyKernel
RPs / NRPs	M35	cross validation *G. max*	0.8025	0.8025	0.8381	0.7509	0.4113	0.4051	Sequential Minimal Optimization with RBF kernel
RPs / NRPs	M35	cross validation *G. max*	0.8915	0.8915	0.8888	0.8877	0.7598	0.6979	LogitBoost
RPs / NRPs	M35	cross validation *G. max*	0.9177	0.9177	0.9175	0.9176	0.8595	0.7799	J48 Decision Tree
RPs / NRPs	M35	cross validation *G. max*	0.9438	0.9438	0.9432	0.9433	0.8909	0.8482	Random Forest
RPs / NRPs	M36	cross validation *A. thaliana*	0.9021	0.9021	0.9028	0.9024	0.8433	0.7408	Naive Bayes
RPs / NRPs	M36	cross validation *A. thaliana*	0.9385	0.9385	0.9378	0.9379	0.8800	0.8335	Multilayer Perceptron
RPs / NRPs	M36	cross validation *A. thaliana*	0.9244	0.9244	0.9239	0.9221	0.8211	0.7921	Sequential Minimal Optimization with PolyKernel
RPs / NRPs	M36	cross validation *A. thaliana*	0.7578	0.7578	0.8169	0.6608	0.2733	0.1531	Sequential Minimal Optimization with RBF kernel
RPs / NRPs	M36	cross validation *A. thaliana*	0.9128	0.9128	0.9112	0.9106	0.8094	0.7598	LogitBoost
RPs / NRPs	M36	cross validation *A. thaliana*	0.9172	0.9172	0.9162	0.9165	0.8471	0.7761	J48 Decision Tree
RPs / NRPs	M36	cross validation *A. thaliana*	0.9428	0.9428	0.9422	0.9420	0.8776	0.8446	Random Forest
RPs / NRPs	M31	jackknife test *Z. mays*	0.8386	0.8386	0.8362	0.8373	0.7151	0.5630	Naive Bayes
RPs / NRPs	M31	jackknife test *Z. mays*	0.8773	0.8773	0.8737	0.8740	0.7479	0.6598	Multilayer Perceptron
RPs / NRPs	M31	jackknife test *Z. mays*	0.8688	0.8688	0.8655	0.8610	0.6878	0.6275	Sequential Minimal Optimization with PolyKernel
RPs / NRPs	M31	jackknife test *Z. mays*	0.8008	0.8008	0.8239	0.7514	0,4161	0,3921	Sequential Minimal Optimization with RBF kernel
RPs / NRPs	M31	jackknife test *Z. mays*	0.8612	0.8612	0.8562	0.8540	0.6853	0.6062	LogitBoost
RPs / NRPs	M31	jackknife test *Z. mays*	0.8771	0.8771	0.8736	0.8741	0.7512	0.6601	J48 Decision Tree
RPs / NRPs	M31	jackknife test *Z. mays*	0.907	0.9070	0.9051	0.9045	0.7981	0.7433	Random Forest
RPs / NRPs	M32	jackknife test *S. lycopersicum*	0.8675	0.8675	0.8676	0.8676	0.7799	0.6470	Naive Bayes

RPs / NRPs	M32	jackknife test	*S. lycopersicum*	0.8866	0.8866	0.8838	0.8843	0.7739	0.6880	Multilayer Perceptron
RPs / NRPs	M32	jackknife test	*S. lycopersicum*	0.8938	0.8938	0.8924	0.8889	0.7460	0.7031	Sequential Minimal Optimization with PolyKernel
RPs / NRPs	M32	jackknife test	*S. lycopersicum*	0.7500	0.7500	0.5625	0.6429	0.2500	0.0000	Sequential Minimal Optimization with RBF kernel
RPs / NRPs	M32	jackknife test	*S. lycopersicum*	0.8825	0.8825	0.8805	0.8761	0.7175	0.6690	LogitBoost
RPs / NRPs	M32	jackknife test	*S. lycopersicum*	0.8722	0.8722	0.8707	0.8713	0.7746	0.6550	J48 Decision Tree
RPs / NRPs	M32	jackknife test	*S. lycopersicum*	0.9077	0.9077	0.9059	0.9054	0.8015	0.7457	Random Forest
RPs / NRPs	M33	jackknife test	*O. sativa*	0.8762	0.8762	0.8733	0.8742	0.7645	0.6611	Naive Bayes
RPs / NRPs	M33	jackknife test	*O. sativa*	0.9160	0.9160	0.9145	0.9143	0.8224	0.7697	Multilayer Perceptron
RPs / NRPs	M33	jackknife test	*O. sativa*	0.9031	0.9031	0.9018	0.8992	0.7710	0.7305	Sequential Minimal Optimization with PolyKernel
RPs / NRPs	M33	jackknife test	*O. sativa*	0.7583	0.7583	0.8172	0.6621	0.2750	0.1589	Sequential Minimal Optimization with RBF kernel
RPs / NRPs	M33	jackknife test	*O. sativa*	0.8905	0.8905	0.8881	0.8862	0.7508	0.6944	LogitBoost
RPs / NRPs	M33	jackknife test	*O. sativa*	0.9003	0.9003	0.8981	0.8984	0.7998	0.7265	J48 Decision Tree
RPs / NRPs	M33	jackknife test	*O. sativa*	0.9253	0.9253	0.9242	0.9240	0.8428	0.7959	Random Forest
RPs / NRPs	M34	jackknife test	*O. lucimarinus*	0.8665	0.8665	0.8675	0.8670	0.785	0.6467	Naive Bayes
RPs / NRPs	M34	jackknife test	*O. lucimarinus*	0.8991	0.8991	0.8969	0.8972	0.7959	0.7230	Multilayer Perceptron
RPs / NRPs	M34	jackknife test	*O. lucimarinus*	0.8949	0.8949	0.8935	0.8901	0.7491	0.7063	Sequential Minimal Optimization with PolyKernel
RPs / NRPs	M34	jackknife test	*O. lucimarinus*	0.7500	0.7500	0.5625	0.6429	0.2500	0.0000	Sequential Minimal Optimization with RBF kernel
RPs / NRPs	M34	jackknife test	*O. lucimarinus*	0.9020	0.9020	0.8999	0.8991	0.7855	0.7288	LogitBoost
RPs / NRPs	M34	jackknife test	*O. lucimarinus*	0.8793	0.8793	0.8762	0.8769	0.7628	0.6679	J48 Decision Tree
RPs / NRPs	M34	jackknife test	*O. lucimarinus*	0.9148	0.9148	0.9133	0.9126	0.8125	0.7654	Random Forest
RPs / NRPs	M35	jackknife test	*G. max*	0.8767	0.8767	0.8753	0.8759	0.7820	0.6673	Naive Bayes
RPs / NRPs	M35	jackknife test	*G. max*	0.9166	0.9166	0.9152	0.9154	0.8352	0.7728	Multilayer Perceptron
RPs / NRPs	M35	jackknife test	*G. max*	0.8998	0.8998	0.8984	0.8956	0.7638	0.7209	Sequential Minimal Optimization with PolyKernel
RPs / NRPs	M35	jackknife test	*G. max*	0.8127	0.8127	0.8427	0.7689	0.4442	0.4439	Sequential Minimal Optimization with RBF kernel
RPs / NRPs	M35	jackknife test	*G. max*	0.8903	0.8903	0.8878	0.8860	0.7508	0.6938	LogitBoost
RPs / NRPs	M35	jackknife test	*G. max*	0.9198	0.9198	0.9192	0.9194	0.8571	0.7843	J48 Decision Tree
RPs / NRPs	M35	jackknife test	*G. max*	0.9431	0.9431	0.9425	0.9426	0.8895	0.8462	Random Forest
RPs / NRPs	M36	jackknife test	*A. thaliana*	0.9016	0.9016	0.9021	0.9019	0.8406	0.7389	Naive Bayes
RPs / NRPs	M36	jackknife test	*A. thaliana*	0.9356	0.9356	0.9348	0.9349	0.8739	0.8255	Multilayer Perceptron
RPs / NRPs	M36	jackknife test	*A. thaliana*	0.9234	0.9234	0.9227	0.9213	0.8220	0.7895	Sequential Minimal Optimization with PolyKernel
RPs / NRPs	M36	jackknife test	*A. thaliana*	0.7597	0.7597	0.8180	0.6651	0.2791	0.1713	Sequential Minimal Optimization with RBF kernel
RPs / NRPs	M36	jackknife test	*A. thaliana*	0.9104	0.9104	0.9086	0.9087	0.8164	0.7544	LogitBoost
RPs / NRPs	M36	jackknife test	*A. thaliana*	0.9293	0.9293	0.9290	0.9291	0.8782	0.8107	J48 Decision Tree
RPs / NRPs	M36	jackknife test	*A. thaliana*	0.9443	0.9443	0.9437	0.9435	0.8807	0.8486	Random Forest
RPs / HPs	M37	*Z. mays*	*A. thaliana*	0.7593	0.7593	0.7462	0.7508	0.5559	0.3386	Naive Bayes
RPs / HPs	M37	*Z. mays*	*A. thaliana*	0.8324	0.8324	0.8247	0.8231	0.6457	0.5344	Multilayer Perceptron
RPs / HPs	M37	*Z. mays*	*A. thaliana*	0.7493	0.7493	0.8128	0.6514	0.2892	0.1695	Sequential Minimal Optimization with PolyKernel
RPs / HPs	M37	*Z. mays*	*A. thaliana*	0.7393	0.7393	0.5465	0.6284	0.2607	0.0000	Sequential Minimal Optimization with RBF kernel
RPs / HPs	M37	*Z. mays*	*A. thaliana*	0.8481	0.8481	0.8439	0.8378	0.6548	0.5777	LogitBoost
RPs / HPs	M37	*Z. mays*	*A. thaliana*	0.8610	0.8610	0.8575	0.8585	0.7447	0.6286	J48 Decision Tree
RPs / HPs	M37	*Z. mays*	*A. thaliana*	0.9226	0.9226	0.9259	0.9189	0.7984	0.7949	Random Forest
RPs / HPs	M38	*S. lycopersicum*	*A. thaliana*	0.7923	0.7923	0.7944	0.7514	0.4537	0.3824	Naive Bayes
RPs / HPs	M38	*S. lycopersicum*	*A. thaliana*	0.8009	0.8009	0.8064	0.7638	0.4745	0.4160	Multilayer Perceptron
RPs / HPs	M38	*S. lycopersicum*	*A. thaliana*	0.7393	0.7393	0.5465	0.6284	0.2607	0.0000	Sequential Minimal Optimization with PolyKernel

RPs / HPs	M38	*S. lycopersicum*	*A. thaliana*	0.7393	0.7393	0.5465	0.6284	0.2607	0.0000	Sequential Minimal Optimization with RBF kernel
RPs / HPs	M38	*S. lycopersicum*	*A. thaliana*	0.8009	0.8009	0.7886	0.7803	0.5421	0.4245	LogitBoost
RPs / HPs	M38	*S. lycopersicum*	*A. thaliana*	0.8582	0.8582	0.8610	0.8451	0.6441	0.6075	J48 Decision Tree
RPs / HPs	M38	*S. lycopersicum*	*A. thaliana*	0.8582	0.8582	0.8766	0.8391	0.6050	0.6156	Random Forest
RPs / HPs	M39	*O. sativa*	*A. thaliana*	0.8066	0.8066	0.8093	0.7745	0.4979	0.4368	Naive Bayes
RPs / HPs	M39	*O. sativa*	*A. thaliana*	0.8152	0.8152	0.8186	0.7871	0.5223	0.4676	Multilayer Perceptron
RPs / HPs	M39	*O. sativa*	*A. thaliana*	0.7493	0.7493	0.8128	0.6514	0.2892	0.1695	Sequential Minimal Optimization with PolyKernel
RPs / HPs	M39	*O. sativa*	*A. thaliana*	0.7393	0.7393	0.5465	0.6284	0.2607	0.0000	Sequential Minimal Optimization with RBF kernel
RPs / HPs	M39	*O. sativa*	*A. thaliana*	0.7908	0.7908	0.7793	0.7605	0.4888	0.3812	LogitBoost
RPs / HPs	M39	*O. sativa*	*A. thaliana*	0.8123	0.8123	0.8206	0.7803	0.5035	0.4587	J48 Decision Tree
RPs / HPs	M39	*O. sativa*	*A. thaliana*	0.8539	0.8539	0.8676	0.8351	0.6035	0.5991	Random Forest
RPs / HPs	M40	*O. lucimarinus*	*A. thaliana*	0.7880	0.7880	0.7770	0.7543	0.4735	0.3686	Naive Bayes
RPs / HPs	M40	*O. lucimarinus*	*A. thaliana*	0.8138	0.8138	0.8032	0.8016	0.6036	0.4764	Multilayer Perceptron
RPs / HPs	M40	*O. lucimarinus*	*A. thaliana*	0.7622	0.7622	0.8201	0.6790	0.3257	0.2579	Sequential Minimal Optimization with PolyKernel
RPs / HPs	M40	*O. lucimarinus*	*A. thaliana*	0.7393	0.7393	0.5465	0.6284	0.2607	0.0000	Sequential Minimal Optimization with RBF kernel
RPs / HPs	M40	*O. lucimarinus*	*A. thaliana*	0.7736	0.7736	0.7524	0.7446	0.4756	0.3265	LogitBoost
RPs / HPs	M40	*O. lucimarinus*	*A. thaliana*	0.8037	0.8037	0.8137	0.7662	0.4755	0.4279	J48 Decision Tree
RPs / HPs	M40	*O. lucimarinus*	*A. thaliana*	0.8381	0.8381	0.8429	0.8183	0.5837	0.5449	Random Forest
RPs / HPs	M41	*G. max*	*A. thaliana*	0.8023	0.8023	0.8100	0.7650	0.4750	0.4219	Naive Bayes
RPs / HPs	M41	*G. max*	*A. thaliana*	0.8510	0.8510	0.8456	0.8457	0.7020	0.5940	Multilayer Perceptron
RPs / HPs	M41	*G. max*	*A. thaliana*	0.7622	0.7622	0.8201	0.6790	0.3257	0.2579	Sequential Minimal Optimization with PolyKernel
RPs / HPs	M41	*G. max*	*A. thaliana*	0.7393	0.7393	0.5465	0.6284	0.2607	0.0000	Sequential Minimal Optimization with RBF kernel
RPs / HPs	M41	*G. max*	*A. thaliana*	0.8052	0.8052	0.8111	0.7704	0.4867	0.4321	LogitBoost
RPs / HPs	M41	*G. max*	*A. thaliana*	0.8395	0.8395	0.8338	0.8286	0.6411	0.5521	J48 Decision Tree
RPs / HPs	M41	*G. max*	*A. thaliana*	0.8940	0.8940	0.9000	0.8861	0.7208	0.7153	Random Forest
RPs / HPs	M42	*Z. mays*	*G. max*	0.7999	0.7999	0.7898	0.7941	0.5198	0.3385	Naive Bayes
RPs / HPs	M42	*Z. mays*	*G. max*	0.8516	0.8516	0.8398	0.8414	0.5718	0.4863	Multilayer Perceptron
RPs / HPs	M42	*Z. mays*	*G. max*	0.8154	0.8154	0.8499	0.7448	0.2553	0.2396	Sequential Minimal Optimization with PolyKernel
RPs / HPs	M42	*Z. mays*	*G. max*	0.8013	0.8013	0.6421	0.7129	0.1987	0.0000	Sequential Minimal Optimization with RBF kernel
RPs / HPs	M42	*Z. mays*	*G. max*	0.8346	0.8346	0.8186	0.8212	0.5200	0.4177	LogitBoost
RPs / HPs	M42	*Z. mays*	*G. max*	0.8730	0.8730	0.8663	0.8679	0.6582	0.5755	J48 Decision Tree
RPs / HPs	M42	*Z. mays*	*G. max*	0.9114	0.9114	0.9093	0.9060	0.7096	0.7027	Random Forest
RPs / HPs	M43	*S. lycopersicum*	*G. max*	0.8287	0.8287	0.8084	0.8090	0.4683	0.3763	Naive Bayes
RPs / HPs	M43	*S. lycopersicum*	*G. max*	0.8907	0.8907	0.8934	0.8773	0.5927	0.6237	Multilayer Perceptron
RPs / HPs	M43	*S. lycopersicum*	*G. max*	0.8013	0.8013	0.6421	0.7129	0.1987	0.0000	Sequential Minimal Optimization with PolyKernel
RPs / HPs	M43	*S. lycopersicum*	*G. max*	0.8013	0.8013	0.6421	0.7129	0.1987	0.0000	Sequential Minimal Optimization with RBF kernel
RPs / HPs	M43	*S. lycopersicum*	*G. max*	0.8648	0.8648	0.8557	0.8519	0.5667	0.5250	LogitBoost
RPs / HPs	M43	*S. lycopersicum*	*G. max*	0.8981	0.8981	0.8989	0.8877	0.6308	0.6520	J48 Decision Tree
RPs / HPs	M43	*S. lycopersicum*	*G. max*	0.9158	0.9158	0.9200	0.9078	0.6772	0.7195	Random Forest
RPs / HPs	M44	*O. sativa*	*G. max*	0.8227	0.8227	0.8012	0.8038	0.4640	0.3573	Naive Bayes
RPs / HPs	M44	*O. sativa*	*G. max*	0.8804	0.8804	0.8743	0.8704	0.6153	0.5866	Multilayer Perceptron
RPs / HPs	M44	*O. sativa*	*G. max*	0.8154	0.8154	0.8499	0.7448	0.2553	0.2396	Sequential Minimal Optimization with PolyKernel
RPs / HPs	M44	*O. sativa*	*G. max*	0.8013	0.8013	0.6421	0.7129	0.1987	0.0000	Sequential Minimal Optimization with RBF kernel
RPs / HPs	M44	*O. sativa*	*G. max*	0.8530	0.8530	0.8408	0.8404	0.5526	0.4836	LogitBoost

RPs / HPs	M44	*O. sativa*	*G. max*	0.8656	0.8656	0.8565	0.8532	0.5725	0.5288	J48 Decision Tree
RPs / HPs	M44	*O. sativa*	*G. max*	0.8818	0.8818	0.8761	0.8720	0.6184	0.5921	Random Forest
RPs / HPs	M45	*O. lucimarinus*	*G. max*	0.8360	0.8360	0.8213	0.8243	0.5344	0.4284	Naive Bayes
RPs / HPs	M45	*O. lucimarinus*	*G. max*	0.8168	0.8168	0.8254	0.8206	0.6526	0.4505	Multilayer Perceptron
RPs / HPs	M45	*O. lucimarinus*	*G. max*	0.8176	0.8176	0.8264	0.7537	0.2754	0.2520	Sequential Minimal Optimization with PolyKernel
RPs / HPs	M45	*O. lucimarinus*	*G. max*	0.8013	0.8013	0.6421	0.7129	0.1987	0.0000	Sequential Minimal Optimization with RBF kernel
RPs / HPs	M45	*O. lucimarinus*	*G. max*	0.8250	0.8250	0.8107	0.8151	0.5316	0.3994	LogitBoost
RPs / HPs	M45	*O. lucimarinus*	*G. max*	0.8464	0.8464	0.8326	0.8330	0.5370	0.4582	J48 Decision Tree
RPs / HPs	M45	*O. lucimarinus*	*G. max*	0.8708	0.8708	0.8636	0.8652	0.6492	0.5666	Random Forest
RPs / HPs	M46	*A. thaliana*	*G. max*	0.8183	0.8183	0.8134	0.8156	0.5831	0.4134	Naive Bayes
RPs / HPs	M46	*A. thaliana*	*G. max*	0.8168	0.8168	0.8016	0.8067	0.5156	0.3716	Multilayer Perceptron
RPs / HPs	M46	*A. thaliana*	*G. max*	0.8242	0.8242	0.8023	0.8036	0.4560	0.3572	Sequential Minimal Optimization with PolyKernel
RPs / HPs	M46	*A. thaliana*	*G. max*	0.8013	0.8013	0.6421	0.7129	0.1987	0.0000	Sequential Minimal Optimization with RBF kernel
RPs / HPs	M46	*A. thaliana*	*G. max*	0.8168	0.8168	0.8158	0.8163	0.6023	0.4215	LogitBoost
RPs / HPs	M46	*A. thaliana*	*G. max*	0.8582	0.8582	0.8686	0.8622	0.7607	0.5845	J48 Decision Tree
RPs / HPs	M46	*A. thaliana*	*G. max*	0.9025	0.9025	0.9034	0.9029	0.7997	0.6964	Random Forest
RPs / HPs	M47	*Z. mays*	*O. lucimarinus*	0.7568	0.7568	0.7931	0.7701	0.6474	0.3616	Naive Bayes
RPs / HPs	M47	*Z. mays*	*O. lucimarinus*	0.7748	0.7748	0.7605	0.7666	0.4755	0.2693	Multilayer Perceptron
RPs / HPs	M47	*Z. mays*	*O. lucimarinus*	0.7973	0.7973	0.8386	0.7117	0.2244	0.1316	Sequential Minimal Optimization with PolyKernel
RPs / HPs	M47	*Z. mays*	*O. lucimarinus*	0.7928	0.7928	0.6285	0.7012	0.2072	0.0000	Sequential Minimal Optimization with RBF kernel
RPs / HPs	M47	*Z. mays*	*O. lucimarinus*	0.7973	0.7973	0.7779	0.7842	0.4814	0.3175	LogitBoost
RPs / HPs	M47	*Z. mays*	*O. lucimarinus*	0.7748	0.7748	0.7896	0.7812	0.6039	0.3578	J48 Decision Tree
RPs / HPs	M47	*Z. mays*	*O. lucimarinus*	0.8694	0.8694	0.8627	0.8576	0.5965	0.5603	Random Forest
RPs / HPs	M48	*S. lycopersicum*	*O. lucimarinus*	0.8559	0.8559	0.8460	0.8418	0.5609	0.5088	Naive Bayes
RPs / HPs	M48	*S. lycopersicum*	*O. lucimarinus*	0.8153	0.8153	0.7909	0.7835	0.4058	0.3203	Multilayer Perceptron
RPs / HPs	M48	*S. lycopersicum*	*O. lucimarinus*	0.7928	0.7928	0.6285	0.7012	0.2072	0.0000	Sequential Minimal Optimization with PolyKernel
RPs / HPs	M48	*S. lycopersicum*	*O. lucimarinus*	0.7928	0.7928	0.6285	0.7012	0.2072	0.0000	Sequential Minimal Optimization with RBF kernel
RPs / HPs	M48	*S. lycopersicum*	*O. lucimarinus*	0.7973	0.7973	0.7925	0.7947	0.5616	0.3681	LogitBoost
RPs / HPs	M48	*S. lycopersicum*	*O. lucimarinus*	0.8108	0.8108	0.7886	0.7924	0.4688	0.3414	J48 Decision Tree
RPs / HPs	M48	*S. lycopersicum*	*O. lucimarinus*	0.8288	0.8288	0.8125	0.8145	0.5217	0.4156	Random Forest
RPs / HPs	M49	*O. sativa*	*O. lucimarinus*	0.8288	0.8288	0.8125	0.8145	0.5217	0.4156	Naive Bayes
RPs / HPs	M49	*O. sativa*	*O. lucimarinus*	0.7973	0.7973	0.7806	0.7866	0.4974	0.3278	Multilayer Perceptron
RPs / HPs	M49	*O. sativa*	*O. lucimarinus*	0.7973	0.7973	0.8386	0.7117	0.2244	0.1316	Sequential Minimal Optimization with PolyKernel
RPs / HPs	M49	*O. sativa*	*O. lucimarinus*	0.7928	0.7928	0.6285	0.7012	0.2072	0.0000	Sequential Minimal Optimization with RBF kernel
RPs / HPs	M49	*O. sativa*	*O. lucimarinus*	0.8243	0.8243	0.8064	0.8085	0.5045	0.3955	LogitBoost
RPs / HPs	M49	*O. sativa*	*O. lucimarinus*	0.8198	0.8198	0.8015	0.8048	0.5033	0.3831	J48 Decision Tree
RPs / HPs	M49	*O. sativa*	*O. lucimarinus*	0.8694	0.8694	0.8694	0.8516	0.5484	0.5556	Random Forest
RPs / HPs	M50	*G. max*	*O. lucimarinus*	0.8514	0.8514	0.8434	0.8311	0.5115	0.4826	Naive Bayes
RPs / HPs	M50	*G. max*	*O. lucimarinus*	0.8108	0.8108	0.8108	0.8108	0.6133	0.4242	Multilayer Perceptron
RPs / HPs	M50	*G. max*	*O. lucimarinus*	0.8018	0.8018	0.7768	0.7357	0.2738	0.1889	Sequential Minimal Optimization with PolyKernel
RPs / HPs	M50	*G. max*	*O. lucimarinus*	0.7928	0.7928	0.6285	0.7012	0.2072	0.0000	Sequential Minimal Optimization with RBF kernel
RPs / HPs	M50	*G. max*	*O. lucimarinus*	0.8333	0.8333	0.8171	0.8159	0.5068	0.4218	LogitBoost
RPs / HPs	M50	*G. max*	*O. lucimarinus*	0.7342	0.7342	0.7490	0.7409	0.5130	0.2351	J48 Decision Tree
RPs / HPs	M50	*G. max*	*O. lucimarinus*	0.8559	0.8559	0.8456	0.8438	0.5770	0.5131	Random Forest

RPs / HPs	M51	*A. thaliana*	*O. lucimarinus*	0.8468	0.8468	0.8360	0.8379	0.5907	0.4924	Naive Bayes
RPs / HPs	M51	*A. thaliana*	*O. lucimarinus*	0.8468	0.8468	0.8350	0.8297	0.5264	0.4705	Multilayer Perceptron
RPs / HPs	M51	*A. thaliana*	*O. lucimarinus*	0.8333	0.8333	0.8236	0.8013	0.4265	0.3961	Sequential Minimal Optimization with PolyKernel
RPs / HPs	M51	*A. thaliana*	*O. lucimarinus*	0.7928	0.7928	0.6285	0.7012	0.2072	0.0000	Sequential Minimal Optimization with RBF kernel
RPs / HPs	M51	*A. thaliana*	*O. lucimarinus*	0.8288	0.8288	0.8236	0.8259	0.6181	0.4625	LogitBoost
RPs / HPs	M51	*A. thaliana*	*O. lucimarinus*	0.8063	0.8063	0.8250	0.8135	0.6925	0.4628	J48 Decision Tree
RPs / HPs	M51	*A. thaliana*	*O. lucimarinus*	0.8604	0.8604	0.8640	0.8620	0.7387	0.5854	Random Forest
RPs / HPs	M52	*Z. mays*	*O. sativa*	0.7819	0.7819	0.8186	0.7960	0.6222	0.3542	Naive Bayes
RPs / HPs	M52	*Z. mays*	*O. sativa*	0.8571	0.8571	0.8641	0.8602	0.6874	0.5218	Multilayer Perceptron
RPs / HPs	M52	*Z. mays*	*O. sativa*	0.8295	0.8295	0.8586	0.7537	0.1794	0.0860	Sequential Minimal Optimization with PolyKernel
RPs / HPs	M52	*Z. mays*	*O. sativa*	0.8280	0.8280	0.6855	0.7500	0.1720	0.0000	Sequential Minimal Optimization with RBF kernel
RPs / HPs	M52	*Z. mays*	*O. sativa*	0.8725	0.8725	0.8651	0.8677	0.6128	0.5234	LogitBoost
RPs / HPs	M52	*Z. mays*	*O. sativa*	0.9017	0.9017	0.9082	0.9042	0.8098	0.6757	J48 Decision Tree
RPs / HPs	M52	*Z. mays*	*O. sativa*	0.9432	0.9432	0.9417	0.9420	0.8184	0.7939	Random Forest
RPs / HPs	M53	*S. lycopersicum*	*O. sativa*	0.8326	0.8326	0.8135	0.8197	0.4559	0.3372	Naive Bayes
RPs / HPs	M53	*S. lycopersicum*	*O. sativa*	0.8786	0.8786	0.8720	0.8601	0.4867	0.5043	Multilayer Perceptron
RPs / HPs	M53	*S. lycopersicum*	*O. sativa*	0.8280	0.8280	0.6855	0.7500	0.1720	0.0000	Sequential Minimal Optimization with PolyKernel
RPs / HPs	M53	*S. lycopersicum*	*O. sativa*	0.8280	0.8280	0.6855	0.7500	0.1720	0.0000	Sequential Minimal Optimization with RBF kernel
RPs / HPs	M53	*S. lycopersicum*	*O. sativa*	0.8571	0.8571	0.8537	0.8553	0.6167	0.4862	LogitBoost
RPs / HPs	M53	*S. lycopersicum*	*O. sativa*	0.8955	0.8955	0.8888	0.8883	0.6246	0.5980	J48 Decision Tree
RPs / HPs	M53	*S. lycopersicum*	*O. sativa*	0.9155	0.9155	0.9121	0.9099	0.6783	0.6790	Random Forest
RPs / HPs	M54	*O. lucimarinus*	*O. sativa*	0.8249	0.8249	0.8152	0.8194	0.5038	0.3499	Naive Bayes
RPs / HPs	M54	*O. lucimarinus*	*O. sativa*	0.8341	0.8341	0.8318	0.8329	0.5694	0.4094	Multilayer Perceptron
RPs / HPs	M54	*O. lucimarinus*	*O. sativa*	0.8372	0.8372	0.8313	0.7761	0.2305	0.2078	Sequential Minimal Optimization with PolyKernel
RPs / HPs	M54	*O. lucimarinus*	*O. sativa*	0.8280	0.8280	0.6855	0.7500	0.1720	0.0000	Sequential Minimal Optimization with RBF kernel
RPs / HPs	M54	*O. lucimarinus*	*O. sativa*	0.8264	0.8264	0.8106	0.8166	0.4688	0.3307	LogitBoost
RPs / HPs	M54	*O. lucimarinus*	*O. sativa*	0.8541	0.8541	0.8366	0.8387	0.4745	0.4082	J48 Decision Tree
RPs / HPs	M54	*O. lucimarinus*	*O. sativa*	0.8833	0.8833	0.8746	0.8758	0.6009	0.5507	Random Forest
RPs / HPs	M55	*G. max*	*O. sativa*	0.8648	0.8648	0.8506	0.8510	0.5051	0.4565	Naive Bayes
RPs / HPs	M55	*G. max*	*O. sativa*	0.8833	0.8833	0.8859	0.8845	0.7282	0.5990	Multilayer Perceptron
RPs / HPs	M55	*G. max*	*O. sativa*	0.8356	0.8356	0.8185	0.7752	0.2302	0.1929	Sequential Minimal Optimization with PolyKernel
RPs / HPs	M55	*G. max*	*O. sativa*	0.8280	0.8280	0.6855	0.7500	0.1720	0.0000	Sequential Minimal Optimization with RBF kernel
RPs / HPs	M55	*G. max*	*O. sativa*	0.8633	0.8633	0.8493	0.8414	0.4411	0.4275	LogitBoost
RPs / HPs	M55	*G. max*	*O. sativa*	0.8863	0.8863	0.8871	0.8867	0.7217	0.6038	J48 Decision Tree
RPs / HPs	M55	*G. max*	*O. sativa*	0.9293	0.9293	0.9270	0.9259	0.7378	0.7364	Random Forest
RPs / HPs	M56	*A. thaliana*	*O. sativa*	0.8372	0.8372	0.8284	0.8321	0.5347	0.3958	Naive Bayes
RPs / HPs	M56	*A. thaliana*	*O. sativa*	0.8372	0.8372	0.8194	0.8251	0.4710	0.3582	Multilayer Perceptron
RPs / HPs	M56	*A. thaliana*	*O. sativa*	0.8525	0.8525	0.8328	0.8316	0.4318	0.3827	Sequential Minimal Optimization with PolyKernel
RPs / HPs	M56	*A. thaliana*	*O. sativa*	0.8280	0.8280	0.6855	0.7500	0.1720	0.0000	Sequential Minimal Optimization with RBF kernel
RPs / HPs	M56	*A. thaliana*	*O. sativa*	0.8495	0.8495	0.8538	0.8515	0.6504	0.4866	LogitBoost
RPs / HPs	M56	*A. thaliana*	*O. sativa*	0.8525	0.8525	0.8821	0.8621	0.7996	0.5717	J48 Decision Tree
RPs / HPs	M56	*A. thaliana*	*O. sativa*	0.9094	0.9094	0.9169	0.9121	0.8397	0.7052	Random Forest
RPs / HPs	M57	*Z. mays*	*S. lycopersicum*	0.7815	0.7815	0.7566	0.7659	0.4137	0.2255	Naive Bayes
RPs / HPs	M57	*Z. mays*	*S. lycopersicum*	0.8195	0.8195	0.7981	0.8025	0.4674	0.3481	Multilayer Perceptron

RPs / HPs	M57	*Z. mays*	*S. lycopersicum*	0.8113	0.8113	0.8472	0.7344	0.2308	0.1844	Sequential Minimal Optimization with PolyKernel
RPs / HPs	M57	*Z. mays*	*S. lycopersicum*	0.8030	0.8030	0.6448	0.7152	0.1970	0.0000	Sequential Minimal Optimization with RBF kernel
RPs / HPs	M57	*Z. mays*	*S. lycopersicum*	0.8179	0.8179	0.7908	0.7913	0.4099	0.3100	LogitBoost
RPs / HPs	M57	*Z. mays*	*S. lycopersicum*	0.8675	0.8675	0.8599	0.8617	0.6377	0.5520	J48 Decision Tree
RPs / HPs	M57	*Z. mays*	*S. lycopersicum*	0.8891	0.8891	0.8884	0.8767	0.5986	0.6137	Random Forest
RPs / HPs	M58	*O. sativa*	*S. lycopersicum*	0.8046	0.8046	0.7675	0.7705	0.3560	0.2340	Naive Bayes
RPs / HPs	M58	*O. sativa*	*S. lycopersicum*	0.8891	0.8891	0.8875	0.8774	0.6050	0.6139	Multilayer Perceptron
RPs / HPs	M58	*O. sativa*	*S. lycopersicum*	0.8079	0.8079	0.8450	0.7269	0.2173	0.1426	Sequential Minimal Optimization with PolyKernel
RPs / HPs	M58	*O. sativa*	*S. lycopersicum*	0.8030	0.8030	0.6448	0.7152	0.1970	0.0000	Sequential Minimal Optimization with RBF kernel
RPs / HPs	M58	*O. sativa*	*S. lycopersicum*	0.8576	0.8576	0.8469	0.8482	0.5846	0.5058	LogitBoost
RPs / HPs	M58	*O. sativa*	*S. lycopersicum*	0.8609	0.8609	0.8509	0.8458	0.5410	0.5026	J48 Decision Tree
RPs / HPs	M58	*O. sativa*	*S. lycopersicum*	0.9023	0.9023	0.9003	0.8946	0.6653	0.6660	Random Forest
RPs / HPs	M59	*O. lucimarinus*	*S. lycopersicum*	0.8079	0.8079	0.7777	0.7824	0.4012	0.2760	Naive Bayes
RPs / HPs	M59	*O. lucimarinus*	*S. lycopersicum*	0.7500	0.7500	0.7460	0.7480	0.4440	0.1972	Multilayer Perceptron
RPs / HPs	M59	*O. lucimarinus*	*S. lycopersicum*	0.8129	0.8129	0.8245	0.7408	0.2438	0.1975	Sequential Minimal Optimization with PolyKernel
RPs / HPs	M59	*O. lucimarinus*	*S. lycopersicum*	0.8030	0.8030	0.6448	0.7152	0.1970	0.0000	Sequential Minimal Optimization with RBF kernel
RPs / HPs	M59	*O. lucimarinus*	*S. lycopersicum*	0.7997	0.7997	0.7665	0.7737	0.3865	0.2439	LogitBoost
RPs / HPs	M59	*O. lucimarinus*	*S. lycopersicum*	0.8212	0.8212	0.7948	0.7900	0.3917	0.3102	J48 Decision Tree
RPs / HPs	M59	*O. lucimarinus*	*S. lycopersicum*	0.8642	0.8642	0.8543	0.8532	0.5799	0.5239	Random Forest
RPs / HPs	M60	*G. max*	*S. lycopersicum*	0.8195	0.8195	0.7927	0.7913	0.4040	0.3116	Naive Bayes
RPs / HPs	M60	*G. max*	*S. lycopersicum*	0.8874	0.8874	0.8867	0.8871	0.7504	0.6419	Multilayer Perceptron
RPs / HPs	M60	*G. max*	*S. lycopersicum*	0.8129	0.8129	0.8245	0.7408	0.2438	0.1975	Sequential Minimal Optimization with PolyKernel
RPs / HPs	M60	*G. max*	*S. lycopersicum*	0.8030	0.8030	0.6448	0.7152	0.1970	0.0000	Sequential Minimal Optimization with RBF kernel
RPs / HPs	M60	*G. max*	*S. lycopersicum*	0.8361	0.8361	0.8257	0.8001	0.3890	0.3688	LogitBoost
RPs / HPs	M60	*G. max*	*S. lycopersicum*	0.8791	0.8791	0.8735	0.8751	0.6786	0.5969	J48 Decision Tree
RPs / HPs	M60	*G. max*	*S. lycopersicum*	0.9172	0.9172	0.9149	0.9132	0.7387	0.7229	Random Forest
RPs / HPs	M61	*A. thaliana*	*S. lycopersicum*	0.7848	0.7848	0.7632	0.7715	0.4335	0.2473	Naive Bayes
RPs / HPs	M61	*A. thaliana*	*S. lycopersicum*	0.7930	0.7930	0.7610	0.7700	0.3912	0.2318	Multilayer Perceptron
RPs / HPs	M61	*A. thaliana*	*S. lycopersicum*	0.8146	0.8146	0.7832	0.7793	0.3647	0.2716	Sequential Minimal Optimization with PolyKernel
RPs / HPs	M61	*A. thaliana*	*S. lycopersicum*	0.8030	0.8030	0.6448	0.7152	0.1970	0.0000	Sequential Minimal Optimization with RBF kernel
RPs / HPs	M61	*A. thaliana*	*S. lycopersicum*	0.8179	0.8179	0.8081	0.8121	0.5495	0.3915	LogitBoost
RPs / HPs	M61	*A. thaliana*	*S. lycopersicum*	0.8493	0.8493	0.8539	0.8514	0.7030	0.5376	J48 Decision Tree
RPs / HPs	M61	*A. thaliana*	*S. lycopersicum*	0.8758	0.8758	0.8709	0.8726	0.6842	0.5900	Random Forest
RPs / HPs	M62	*S. lycopersicum*	*Z. mays*	0.7815	0.7815	0.7444	0.7481	0.3832	0.2277	Naive Bayes
RPs / HPs	M62	*S. lycopersicum*	*Z. mays*	0.8197	0.8197	0.8059	0.7877	0.4358	0.3760	Multilayer Perceptron
RPs / HPs	M62	*S. lycopersicum*	*Z. mays*	0.7809	0.7809	0.6098	0.6849	0.2191	0.0000	Sequential Minimal Optimization with PolyKernel
RPs / HPs	M62	*S. lycopersicum*	*Z. mays*	0.7809	0.7809	0.6098	0.6849	0.2191	0.0000	Sequential Minimal Optimization with RBF kernel
RPs / HPs	M62	*S. lycopersicum*	*Z. mays*	0.8136	0.8136	0.7946	0.7953	0.4978	0.3813	LogitBoost
RPs / HPs	M62	*S. lycopersicum*	*Z. mays*	0.8447	0.8447	0.8339	0.8321	0.5776	0.4980	J48 Decision Tree
RPs / HPs	M62	*S. lycopersicum*	*Z. mays*	0.8541	0.8541	0.8473	0.8387	0.5675	0.5247	Random Forest
RPs / HPs	M63	*O. sativa*	*Z. mays*	0.7959	0.7959	0.7672	0.7669	0.4200	0.2915	Naive Bayes
RPs / HPs	M63	*O. sativa*	*Z. mays*	0.8414	0.8414	0.8306	0.8244	0.5420	0.4781	Multilayer Perceptron
RPs / HPs	M63	*O. sativa*	*Z. mays*	0.7826	0.7826	0.7864	0.6898	0.2268	0.0741	Sequential Minimal Optimization with PolyKernel
RPs / HPs	M63	*O. sativa*	*Z. mays*	0.7809	0.7809	0.6098	0.6849	0.2191	0.0000	Sequential Minimal Optimization with RBF kernel

RPs / HPs	M63	*O. sativa*	*Z. mays*	0.7959	0.7959	0.7695	0.7719	0.4437	0.3058	LogitBoost
RPs / HPs	M63	*O. sativa*	*Z. mays*	0.8281	0.8281	0.8131	0.8097	0.5164	0.4295	J48 Decision Tree
RPs / HPs	M63	*O. sativa*	*Z. mays*	0.8569	0.8569	0.8529	0.8398	0.5591	0.5332	Random Forest
RPs / HPs	M64	*O. lucimarinus*	*Z. mays*	0.7754	0.7754	0.7431	0.7508	0.4124	0.2355	Naive Bayes
RPs / HPs	M64	*O. lucimarinus*	*Z. mays*	0.7576	0.7576	0.7495	0.7532	0.5003	0.2675	Multilayer Perceptron
RPs / HPs	M64	*O. lucimarinus*	*Z. mays*	0.7865	0.7865	0.8015	0.6997	0.2425	0.1365	Sequential Minimal Optimization with PolyKernel
RPs / HPs	M64	*O. lucimarinus*	*Z. mays*	0.7809	0.7809	0.6098	0.6849	0.2191	0.0000	Sequential Minimal Optimization with RBF kernel
RPs / HPs	M64	*O. lucimarinus*	*Z. mays*	0.7665	0.7665	0.7297	0.7393	0.3881	0.1969	LogitBoost
RPs / HPs	M64	*O. lucimarinus*	*Z. mays*	0.7948	0.7948	0.7640	0.7604	0.3960	0.2737	J48 Decision Tree
RPs / HPs	M64	*O. lucimarinus*	*Z. mays*	0.8148	0.8148	0.7952	0.7933	0.4817	0.3769	Random Forest
RPs / HPs	M65	*G. max*	*Z. mays*	0.8031	0.8031	0.7776	0.7694	0.4093	0.3072	Naive Bayes
RPs / HPs	M65	*G. max*	*Z. mays*	0.8342	0.8342	0.8306	0.8322	0.6584	0.5045	Multilayer Perceptron
RPs / HPs	M65	*G. max*	*Z. mays*	0.7898	0.7898	0.8053	0.7081	0.2562	0.1732	Sequential Minimal Optimization with PolyKernel
RPs / HPs	M65	*G. max*	*Z. mays*	0.7809	0.7809	0.6098	0.6849	0.2191	0.0000	Sequential Minimal Optimization with RBF kernel
RPs / HPs	M65	*G. max*	*Z. mays*	0.8014	0.8014	0.7750	0.7699	0.4161	0.3055	LogitBoost
RPs / HPs	M65	*G. max*	*Z. mays*	0.8425	0.8425	0.8349	0.8373	0.6389	0.5143	J48 Decision Tree
RPs / HPs	M65	*G. max*	*Z. mays*	0.8680	0.8680	0.8622	0.8575	0.6242	0.5786	Random Forest
RPs / HPs	M66	*A. thaliana*	*Z. mays*	0.7831	0.7831	0.7636	0.7701	0.4856	0.3034	Naive Bayes
RPs / HPs	M66	*A. thaliana*	*Z. mays*	0.8003	0.8003	0.7785	0.7823	0.4813	0.3388	Multilayer Perceptron
RPs / HPs	M66	*A. thaliana*	*Z. mays*	0.8136	0.8136	0.7980	0.7767	0.4086	0.3446	Sequential Minimal Optimization with PolyKernel
RPs / HPs	M66	*A. thaliana*	*Z. mays*	0.7809	0.7809	0.6098	0.6849	0.2191	0.0000	Sequential Minimal Optimization with RBF kernel
RPs / HPs	M66	*A. thaliana*	*Z. mays*	0.7992	0.7992	0.7884	0.7926	0.5557	0.3792	LogitBoost
RPs / HPs	M66	*A. thaliana*	*Z. mays*	0.8075	0.8075	0.8151	0.8109	0.6673	0.4587	J48 Decision Tree
RPs / HPs	M66	*A. thaliana*	*Z. mays*	0.8708	0.8708	0.8689	0.8697	0.7361	0.6166	Random Forest
RPs / HPs	M67	cross validation *Z. mays*		0.7582	0.7582	0.7595	0.7588	0.5406	0.2971	Naive Bayes
RPs / HPs	M67	cross validation *Z. mays*		0.8625	0.8625	0.8551	0.8554	0.6499	0.5683	Multilayer Perceptron
RPs / HPs	M67	cross validation *Z. mays*		0.7815	0.7815	0.7565	0.6872	0.2229	0.0442	Sequential Minimal Optimization with PolyKernel
RPs / HPs	M67	cross validation *Z. mays*		0.7809	0.7809	0.6098	0.6849	0.2191	0.0000	Sequential Minimal Optimization with RBF kernel
RPs / HPs	M67	cross validation *Z. mays*		0.8308	0.8308	0.8173	0.8102	0.5063	0.4347	LogitBoost
RPs / HPs	M67	cross validation *Z. mays*		0.8841	0.8841	0.8803	0.8814	0.7343	0.6481	J48 Decision Tree
RPs / HPs	M67	cross validation *Z. mays*		0.9185	0.9185	0.9169	0.9153	0.7749	0.7507	Random Forest
RPs / HPs	M68	cross validation *S. lycopersicum*		0.8030	0.8030	0.7643	0.7678	0.3492	0.2238	Naive Bayes
RPs / HPs	M68	cross validation *S. lycopersicum*		0.8874	0.8874	0.8817	0.8820	0.6743	0.6191	Multilayer Perceptron
RPs / HPs	M68	cross validation *S. lycopersicum*		0.8030	0.8030	0.6448	0.7152	0.1970	0.0000	Sequential Minimal Optimization with PolyKernel
RPs / HPs	M68	cross validation *S. lycopersicum*		0.8030	0.8030	0.6448	0.7152	0.1970	0.0000	Sequential Minimal Optimization with RBF kernel
RPs / HPs	M68	cross validation *S. lycopersicum*		0.8626	0.8626	0.8529	0.8481	0.5477	0.5099	LogitBoost
RPs / HPs	M68	cross validation *S. lycopersicum*		0.8874	0.8874	0.8825	0.8783	0.6299	0.6103	J48 Decision Tree
RPs / HPs	M68	cross validation *S. lycopersicum*		0.9139	0.9139	0.9156	0.9065	0.6808	0.7093	Random Forest
RPs / HPs	M69	cross validation *O. sativa*		0.8433	0.8433	0.8276	0.8325	0.4935	0.3872	Naive Bayes
RPs / HPs	M69	cross validation *O. sativa*		0.9109	0.9109	0.9069	0.9077	0.7127	0.6696	Multilayer Perceptron
RPs / HPs	M69	cross validation *O. sativa*		0.8295	0.8295	0.8586	0.7537	0.1794	0.0860	Sequential Minimal Optimization with PolyKernel
RPs / HPs	M69	cross validation *O. sativa*		0.8280	0.8280	0.6855	0.7500	0.1720	0.0000	Sequential Minimal Optimization with RBF kernel
RPs / HPs	M69	cross validation *O. sativa*		0.8694	0.8694	0.8576	0.8594	0.5485	0.4884	LogitBoost
RPs / HPs	M69	cross validation *O. sativa*		0.9032	0.9032	0.8978	0.8982	0.6687	0.6343	J48 Decision Tree

RPs / HPs	M69	cross validation *O. sativa*	0.9401	0.9401	0.9392	0.9370	0.7612	0.7779	Random Forest
RPs / HPs	M70	cross validation *O. lucimarinus*	0.8153	0.8153	0.8057	0.8095	0.5664	0.4065	Naive Bayes
RPs / HPs	M70	cross validation *O. lucimarinus*	0.8468	0.8468	0.8404	0.8428	0.6388	0.5122	Multilayer Perceptron
RPs / HPs	M70	cross validation *O. lucimarinus*	0.7973	0.7973	0.7600	0.7264	0.2566	0.1471	Sequential Minimal Optimization with PolyKernel
RPs / HPs	M70	cross validation *O. lucimarinus*	0.7928	0.7928	0.6285	0.7012	0.2072	0.0000	Sequential Minimal Optimization with RBF kernel
RPs / HPs	M70	cross validation *O. lucimarinus*	0.8333	0.8333	0.8198	0.8226	0.5550	0.4424	LogitBoost
RPs / HPs	M70	cross validation *O. lucimarinus*	0.8468	0.8468	0.8386	0.8413	0.6228	0.5053	J48 Decision Tree
RPs / HPs	M70	cross validation *O. lucimarinus*	0.8964	0.8964	0.8925	0.8931	0.7321	0.6694	Random Forest
RPs / HPs	M71	cross validation *G. max*	0.8434	0.8434	0.8288	0.8294	0.5278	0.4459	Naive Bayes
RPs / HPs	M71	cross validation *G. max*	0.9269	0.9269	0.9268	0.9268	0.8421	0.7700	Multilayer Perceptron
RPs / HPs	M71	cross validation *G. max*	0.8176	0.8176	0.8264	0.7537	0.2754	0.2520	Sequential Minimal Optimization with PolyKernel
RPs / HPs	M71	cross validation *G. max*	0.8013	0.8013	0.6421	0.7129	0.1987	0.0000	Sequential Minimal Optimization with RBF kernel
RPs / HPs	M71	cross validation *G. max*	0.8671	0.8671	0.8587	0.8538	0.5672	0.5325	LogitBoost
RPs / HPs	M71	cross validation *G. max*	0.9306	0.9306	0.9290	0.9293	0.8178	0.7757	J48 Decision Tree
RPs / HPs	M71	cross validation *G. max*	0.9616	0.9616	0.9614	0.9607	0.8759	0.8764	Random Forest
RPs / HPs	M72	cross validation *A. thaliana*	0.8381	0.8381	0.8312	0.8305	0.6655	0.5536	Naive Bayes
RPs / HPs	M72	cross validation *A. thaliana*	0.9026	0.9026	0.9007	0.9009	0.8127	0.7407	Multilayer Perceptron
RPs / HPs	M72	cross validation *A. thaliana*	0.8209	0.8209	0.8292	0.7929	0.5278	0.4887	Sequential Minimal Optimization with PolyKernel
RPs / HPs	M72	cross validation *A. thaliana*	0.7393	0.7393	0.5465	0.6284	0.2607	0.0000	Sequential Minimal Optimization with RBF kernel
RPs / HPs	M72	cross validation *A. thaliana*	0.8438	0.8438	0.8390	0.8330	0.6462	0.5647	LogitBoost
RPs / HPs	M72	cross validation *A. thaliana*	0.9112	0.9112	0.9109	0.9110	0.8549	0.7688	J48 Decision Tree
RPs / HPs	M72	cross validation *A. thaliana*	0.9484	0.9484	0.9485	0.9474	0.8822	0.8639	Random Forest
RPs / HPs	M67	jackknife test *Z. mays*	0.7598	0.7598	0.7605	0.7602	0.5410	0.3001	Naive Bayes
RPs / HPs	M67	jackknife test *Z. mays*	0.8591	0.8591	0.8528	0.8545	0.6709	0.5662	Multilayer Perceptron
RPs / HPs	M67	jackknife test *Z. mays*	0.7804	0.7804	0.6097	0.6846	0.2189	-0.0125	Sequential Minimal Optimization with PolyKernel
RPs / HPs	M67	jackknife test *Z. mays*	0.7809	0.7809	0.6098	0.6849	0.2191	0.0000	Sequential Minimal Optimization with RBF kernel
RPs / HPs	M67	jackknife test *Z. mays*	0.8414	0.8414	0.8298	0.8276	0.5639	0.4842	LogitBoost
RPs / HPs	M67	jackknife test *Z. mays*	0.8808	0.8808	0.8796	0.8801	0.7607	0.6481	J48 Decision Tree
RPs / HPs	M67	jackknife test *Z. mays*	0.9212	0.9212	0.9198	0.9183	0.7830	0.7596	Random Forest
RPs / HPs	M68	jackknife test *S. lycopersicum*	0.8046	0.8046	0.7666	0.7690	0.3496	0.2290	Naive Bayes
RPs / HPs	M68	jackknife test *S. lycopersicum*	0.8642	0.8642	0.8590	0.8610	0.6623	0.5526	Multilayer Perceptron
RPs / HPs	M68	jackknife test *S. lycopersicum*	0.8030	0.8030	0.6448	0.7152	0.1970	0.0000	Sequential Minimal Optimization with PolyKernel
RPs / HPs	M68	jackknife test *S. lycopersicum*	0.8030	0.8030	0.6448	0.7152	0.1970	0.0000	Sequential Minimal Optimization with RBF kernel
RPs / HPs	M68	jackknife test *S. lycopersicum*	0.8510	0.8510	0.8385	0.8321	0.5005	0.4574	LogitBoost
RPs / HPs	M68	jackknife test *S. lycopersicum*	0.8841	0.8841	0.8780	0.8759	0.6355	0.6002	J48 Decision Tree
RPs / HPs	M68	jackknife test *S. lycopersicum*	0.9156	0.9156	0.9171	0.9085	0.6876	0.7154	Random Forest
RPs / HPs	M69	jackknife test *O. sativa*	0.8449	0.8449	0.8290	0.8338	0.4939	0.3915	Naive Bayes
RPs / HPs	M69	jackknife test *O. sativa*	0.9278	0.9278	0.9252	0.9248	0.7445	0.7317	Multilayer Perceptron
RPs / HPs	M69	jackknife test *O. sativa*	0.8295	0.8295	0.8586	0.7537	0.1794	0.0860	Sequential Minimal Optimization with PolyKernel
RPs / HPs	M69	jackknife test *O. sativa*	0.8280	0.8280	0.6855	0.7500	0.1720	0.0000	Sequential Minimal Optimization with RBF kernel
RPs / HPs	M69	jackknife test *O. sativa*	0.9017	0.9017	0.8960	0.8959	0.6542	0.6258	LogitBoost
RPs / HPs	M69	jackknife test *O. sativa*	0.9078	0.9078	0.9041	0.9000	0.6343	0.6446	J48 Decision Tree
RPs / HPs	M69	jackknife test *O. sativa*	0.9416	0.9416	0.9404	0.9390	0.7757	0.7843	Random Forest
RPs / HPs	M70	jackknife test *O. lucimarinus*	0.8333	0.8333	0.8250	0.8281	0.6032	0.4649	Naive Bayes

RPs / HPs	M70	jackknife test *O. lucimarinus*	0.8514	0.8514	0.8502	0.8507	0.6882	0.5439	Multilayer Perceptron
RPs / HPs	M70	jackknife test *O. lucimarinus*	0.7973	0.7973	0.7600	0.7264	0.2566	0.1471	Sequential Minimal Optimization with PolyKernel
RPs / HPs	M70	jackknife test *O. lucimarinus*	0.7928	0.7928	0.6285	0.7012	0.2072	0.0000	Sequential Minimal Optimization with RBF kernel
RPs / HPs	M70	jackknife test *O. lucimarinus*	0.8423	0.8423	0.8294	0.8302	0.5574	0.4674	LogitBoost
RPs / HPs	M70	jackknife test *O. lucimarinus*	0.8739	0.8739	0.8670	0.8650	0.6298	0.5812	J48 Decision Tree
RPs / HPs	M70	jackknife test *O. lucimarinus*	0.9054	0.9054	0.9022	0.9024	0.7505	0.6986	Random Forest
RPs / HPs	M71	jackknife test *G. max*	0.8427	0.8427	0.8282	0.8295	0.5332	0.4460	Naive Bayes
RPs / HPs	M71	jackknife test *G. max*	0.9180	0.9180	0.9175	0.9177	0.8175	0.7408	Multilayer Perceptron
RPs / HPs	M71	jackknife test *G. max*	0.8176	0.8176	0.8264	0.7537	0.2754	0.2520	Sequential Minimal Optimization with PolyKernel
RPs / HPs	M71	jackknife test *G. max*	0.8013	0.8013	0.6421	0.7129	0.1987	0.0000	Sequential Minimal Optimization with RBF kernel
RPs / HPs	M71	jackknife test *G. max*	0.8456	0.8456	0.8313	0.8309	0.5256	0.4516	LogitBoost
RPs / HPs	M71	jackknife test *G. max*	0.9321	0.9321	0.9304	0.9303	0.8070	0.7786	J48 Decision Tree
RPs / HPs	M71	jackknife test *G. max*	0.9616	0.9616	0.9616	0.9606	0.8703	0.8764	Random Forest
RPs / HPs	M72	jackknife test *A. thaliana*	0.8381	0.8381	0.8312	0.8305	0.6655	0.5536	Naive Bayes
RPs / HPs	M72	jackknife test *A. thaliana*	0.9026	0.9026	0.9008	0.9011	0.8163	0.7413	Multilayer Perceptron
RPs / HPs	M72	jackknife test *A. thaliana*	0.8281	0.8281	0.8346	0.8039	0.5517	0.5125	Sequential Minimal Optimization with PolyKernel
RPs / HPs	M72	jackknife test *A. thaliana*	0.7393	0.7393	0.5465	0.6284	0.2607	0.0000	Sequential Minimal Optimization with RBF kernel
RPs / HPs	M72	jackknife test *A. thaliana*	0.8424	0.8424	0.8373	0.8386	0.7061	0.5755	LogitBoost
RPs / HPs	M72	jackknife test *A. thaliana*	0.9169	0.9169	0.9164	0.9166	0.8604	0.7830	J48 Decision Tree
RPs / HPs	M72	jackknife test *A. thaliana*	0.9456	0.9456	0.9457	0.9444	0.8741	0.8561	Random Forest

For each algorithm, we used the default Weka parameters. The only variation was the use of SMO with and without a kernel function, i.e., (i) Multilayer Perceptron: "*MultilayerPerceptron -L 0.3 -M 0.2 -N 500 -V 0 -S 0 -E 20 -H a*", (ii) Random Forest: "*RandomForest -I 100 -K 0 -S 1 -num-slots 1*", (iii) Sequential Minimal Optimization: "*SMO -C 1.0 -L 0.001 -P 1.0E-12 -N 0 -V -1 -W 1 –K*" with kernels "*PolyKernel -E 1.0 -C 250007*" and "*RBFKernel -G 0.01 -C 250007*", (iv) LogitBoost: "*LogitBoost -P 100 -L -1.7976931348623157E308 -H 1.0 -Z 3.0 -O 1 -E 1 -S 1 -I 10 -W DecisionStump*", J48Decision Tree: "*J48 -C 0.25 -M 2*", and Naive Bayes: "*NaiveBayes -num-decimal-places 2 -batch-size 100*". Notice that the use of default parameters of PolyKernel (exponent=1.0) means no kernel function. Therefore, SMO is tested with the RBF kernel function and without kernel function (linear separation).

Supplementary Table S4. RNA-binding proteins tested in Rama.

ID	Phytozome description	Specie	Classification	Probability
AT1G18630.1	glycine-rich RNA-binding protein 7, putative, expressed	*A. thaliana*	Not Ribosomal	0.66
AT2G02410.1	RNA-binding protein containing a PIN domain, putative, expressed	*A. thaliana*	Not Ribosomal	0.88
AT2G02410.2	RNA-binding protein containing a PIN domain, putative, expressed	*A. thaliana*	Not Ribosomal	0.83
AT2G03640.1	RNA-binding protein-like, putative, expressed	*A. thaliana*	Not Ribosomal	0.99
AT2G03640.2	RNA-binding protein-like, putative, expressed	*A. thaliana*	Not Ribosomal	0.99
AT2G03640.3	RNA-binding protein-like, putative, expressed	*A. thaliana*	Not Ribosomal	0.99
AT2G03640.4	RNA-binding protein-like, putative, expressed	*A. thaliana*	Not Ribosomal	0.98
AT3G03340.1	RNA-binding protein Luc7-like, putative, expressed	*A. thaliana*	Not Ribosomal	0.69
AT3G07250.1	RNA-binding protein-like, putative, expressed	*A. thaliana*	Not Ribosomal	1
AT3G25150.1	RNA-binding protein-like, putative, expressed	*A. thaliana*	Not Ribosomal	1
AT3G25150.2	RNA-binding protein-like, putative, expressed	*A. thaliana*	Not Ribosomal	1
AT3G55540.1	RNA-binding protein-like, putative, expressed	*A. thaliana*	Not Ribosomal	0.99
AT4G13850.1	glycine-rich RNA-binding protein 7, putative, expressed	*A. thaliana*	Not Ribosomal	0.69
AT4G13850.2	glycine-rich RNA-binding protein 7, putative, expressed	*A. thaliana*	Not Ribosomal	0.71
AT4G13850.3	glycine-rich RNA-binding protein 7, putative, expressed	*A. thaliana*	Not Ribosomal	0.74
AT4G13850.4	glycine-rich RNA-binding protein 7, putative, expressed	*A. thaliana*	Not Ribosomal	0.77
AT4G16830.2	plasminogen activator inhibitor 1 RNA-binding protein, putative, expressed	*A. thaliana*	Not Ribosomal	0.8
AT4G16830.3	plasminogen activator inhibitor 1 RNA-binding protein, putative, expressed	*A. thaliana*	Not Ribosomal	0.76
AT4G28990.1	RNA-binding protein-related, putative, expressed	*A. thaliana*	Not Ribosomal	0.64
AT4G28990.2	RNA-binding protein-related, putative, expressed	*A. thaliana*	Not Ribosomal	0.58
AT5G17440.1	RNA-binding protein Luc7-like, putative, expressed	*A. thaliana*	Not Ribosomal	0.69
AT5G47210.2	plasminogen activator inhibitor 1 RNA-binding protein, putative, expressed	*A. thaliana*	Not Ribosomal	0.81
AT5G47210.3	plasminogen activator inhibitor 1 RNA-binding protein, putative, expressed	*A. thaliana*	Not Ribosomal	0.8
AT5G48650.1	RNA-binding protein-like, putative, expressed	*A. thaliana*	Not Ribosomal	1
AT5G51410.1	RNA-binding protein Luc7-like, putative, expressed	*A. thaliana*	Not Ribosomal	0.71
AT5G51410.2	RNA-binding protein Luc7-like, putative, expressed	*A. thaliana*	Not Ribosomal	0.71
AT5G51410.3	RNA-binding protein Luc7-like, putative, expressed	*A. thaliana*	Not Ribosomal	0.69
Glyma.01G234800.1.p	Alba DNA/RNA-binding protein	*G. max*	Ribosomal	0.44
Glyma.02G134400.1.p	RNA-binding protein 47B	*G. max*	Not Ribosomal	1
Glyma.02G135300.1.p	RNA-binding protein	*G. max*	Not Ribosomal	0.95
Glyma.02G152000.1.p	RNA-binding protein-defense related 1	*G. max*	Not Ribosomal	0.99
Glyma.02G152000.2.p	RNA-binding protein-defense related 1	*G. max*	Not Ribosomal	0.99
Glyma.02G275300.1.p	LA RNA-binding protein	*G. max*	Not Ribosomal	1
Glyma.02G288800.1.p	DNA/RNA-binding protein Kin17, conserved region	*G. max*	Not Ribosomal	0.51
Glyma.03G061500.1.p	glycine-rich RNA-binding protein 3	*G. max*	Not Ribosomal	0.88
Glyma.03G140400.1.p	glycine-rich RNA-binding protein 3	*G. max*	Not Ribosomal	0.76
Glyma.03G202900.1.p	chloroplast RNA-binding protein 33	*G. max*	Not Ribosomal	0.97
Glyma.03G208500.2.p	RNA-binding protein-defense related 1	*G. max*	Not Ribosomal	0.99
Glyma.03G243600.1.p	dsRNA-binding protein 5	*G. max*	Not Ribosomal	1
Glyma.03G243600.2.p	dsRNA-binding protein 2	*G. max*	Not Ribosomal	0.99
Glyma.03G243600.3.p	dsRNA-binding protein 2	*G. max*	Not Ribosomal	0.99
Glyma.03G243600.4.p	dsRNA-binding protein 2	*G. max*	Not Ribosomal	0.99
Glyma.03G243600.5.p	dsRNA-binding protein 5	*G. max*	Not Ribosomal	1
Glyma.03G248100.1.p	Alba DNA/RNA-binding protein	*G. max*	Not Ribosomal	0.63
Glyma.03G264600.1.p	RNA-binding protein	*G. max*	Not Ribosomal	0.98
Glyma.03G264600.2.p	RNA-binding protein	*G. max*	Not Ribosomal	0.98
Glyma.03G264600.3.p	RNA-binding protein	*G. max*	Not Ribosomal	0.91
Glyma.04G037100.1.p	RNA-binding protein 47C	*G. max*	Not Ribosomal	1
Glyma.04G037100.2.p	RNA-binding protein 47C	*G. max*	Not Ribosomal	0.99
Glyma.04G068800.1.p	Plant Tudor-like RNA-binding protein	*G. max*	Not Ribosomal	0.99
Glyma.04G154800.1.p	dsRNA-binding protein 2	*G. max*	Not Ribosomal	0.77
Glyma.04G217100.1.p	Alba DNA/RNA-binding protein	*G. max*	Not Ribosomal	0.59
Glyma.05G010000.1.p	Plant Tudor-like RNA-binding protein	*G. max*	Not Ribosomal	0.98
Glyma.05G020900.1.p	glycine-rich RNA-binding protein 3	*G. max*	Not Ribosomal	0.95
Glyma.05G020900.2.p	glycine-rich RNA-binding protein 3	*G. max*	Not Ribosomal	0.95
Glyma.05G043200.1.p	chloroplast RNA-binding protein 31B	*G. max*	Not Ribosomal	0.97

Glyma.05G090000.2.p	double-stranded-RNA-binding protein 4	*G. max*	Not Ribosomal	1
Glyma.05G090000.3.p	double-stranded-RNA-binding protein 4	*G. max*	Not Ribosomal	0.99
Glyma.05G090000.4.p	double-stranded-RNA-binding protein 4	*G. max*	Not Ribosomal	0.99
Glyma.05G100100.1.p	dsRNA-binding protein 2	*G. max*	Not Ribosomal	0.77
Glyma.06G037600.1.p	RNA-binding protein 47C	*G. max*	Not Ribosomal	0.99
Glyma.06G037600.2.p	RNA-binding protein 47C	*G. max*	Not Ribosomal	0.99
Glyma.06G049000.1.p	RNA-binding protein 1	*G. max*	Not Ribosomal	1
Glyma.06G049000.2.p	RNA-binding protein 1	*G. max*	Not Ribosomal	1
Glyma.06G070400.1.p	Plant Tudor-like RNA-binding protein	*G. max*	Not Ribosomal	0.84
Glyma.06G070400.2.p	Plant Tudor-like RNA-binding protein	*G. max*	Not Ribosomal	0.84
Glyma.06G070400.3.p	Plant Tudor-like RNA-binding protein	*G. max*	Not Ribosomal	0.84
Glyma.06G070400.4.p	Plant Tudor-like RNA-binding protein	*G. max*	Not Ribosomal	0.84
Glyma.06G148800.1.p	Alba DNA/RNA-binding protein	*G. max*	Not Ribosomal	0.56
Glyma.06G148800.2.p	Alba DNA/RNA-binding protein	*G. max*	Not Ribosomal	0.55
Glyma.06G148800.3.p	Alba DNA/RNA-binding protein	*G. max*	Not Ribosomal	0.56
Glyma.06G203800.2.p	glycine-rich RNA-binding protein 3	*G. max*	Not Ribosomal	0.99
Glyma.06G203800.3.p	glycine-rich RNA-binding protein 3	*G. max*	Not Ribosomal	0.99
Glyma.06G203800.4.p	glycine-rich RNA-binding protein 3	*G. max*	Not Ribosomal	0.99
Glyma.06G203800.5.p	glycine-rich RNA-binding protein 3	*G. max*	Not Ribosomal	0.99
Glyma.06G203800.6.p	glycine-rich RNA-binding protein 3	*G. max*	Not Ribosomal	0.99
Glyma.06G233100.1.p	dsRNA-binding protein 2	*G. max*	Ribosomal	0.43
Glyma.07G026400.1.p	S15/NS1, RNA-binding protein	*G. max*	Not Ribosomal	0.85
Glyma.07G031600.1.p	Alba DNA/RNA-binding protein	*G. max*	Not Ribosomal	0.91
Glyma.07G084500.1.p	RNA-binding protein-related	*G. max*	Not Ribosomal	0.62
Glyma.07G084500.2.p	RNA-binding protein-related	*G. max*	Not Ribosomal	0.65
Glyma.07G084500.3.p	RNA-binding protein-related	*G. max*	Not Ribosomal	0.55
Glyma.07G084500.4.p	RNA-binding protein-related	*G. max*	Not Ribosomal	0.55
Glyma.07G209600.1.p	RNA-binding protein	*G. max*	Not Ribosomal	0.97
Glyma.07G210800.1.p	RNA-binding protein 47B	*G. max*	Not Ribosomal	1
Glyma.08G000100.1.p	glycine-rich RNA-binding protein 2	*G. max*	Ribosomal	0.5
Glyma.08G211200.1.p	Alba DNA/RNA-binding protein	*G. max*	Not Ribosomal	0.83
Glyma.08G211200.2.p	Alba DNA/RNA-binding protein	*G. max*	Not Ribosomal	0.62
Glyma.08G216000.1.p	S15/NS1, RNA-binding protein	*G. max*	Not Ribosomal	0.84
Glyma.08G245200.1.p	glycine-rich RNA-binding protein 3	*G. max*	Not Ribosomal	0.95
Glyma.08G313900.1.p	dsRNA-binding protein 5	*G. max*	Not Ribosomal	0.99
Glyma.08G356000.1.p	Alba DNA/RNA-binding protein	*G. max*	Not Ribosomal	0.9
Glyma.09G192400.1.p	RNA-binding protein-related	*G. max*	Not Ribosomal	0.63
Glyma.09G192400.2.p	RNA-binding protein-related	*G. max*	Not Ribosomal	0.58
Glyma.09G192400.3.p	RNA-binding protein-related	*G. max*	Not Ribosomal	0.58
Glyma.10G022200.1.p	RNA-binding protein-defense related 1	*G. max*	Not Ribosomal	0.98
Glyma.10G022200.2.p	RNA-binding protein-defense related 1	*G. max*	Not Ribosomal	0.98
Glyma.10G022200.4.p	RNA-binding protein-defense related 1	*G. max*	Not Ribosomal	0.96
Glyma.10G022200.5.p	RNA-binding protein-defense related 1	*G. max*	Not Ribosomal	0.99
Glyma.10G058500.1.p	chloroplast RNA-binding protein 29	*G. max*	Not Ribosomal	0.98
Glyma.10G082600.1.p	chloroplast RNA-binding protein 33	*G. max*	Not Ribosomal	0.97
Glyma.10G106200.1.p	glycine-rich RNA-binding protein 2	*G. max*	Ribosomal	0.41
Glyma.10G163500.1.p	Alba DNA/RNA-binding protein	*G. max*	Not Ribosomal	0.61
Glyma.11G145900.1.p	dsRNA-binding protein 2	*G. max*	Not Ribosomal	0.95
Glyma.11G255000.1.p	glycine-rich RNA-binding protein 2	*G. max*	Not Ribosomal	0.88
Glyma.11G255000.2.p	glycine-rich RNA-binding protein 2	*G. max*	Not Ribosomal	0.92
Glyma.12G065000.1.p	glycine-rich RNA-binding protein 2	*G. max*	Ribosomal	0.28
Glyma.12G065300.1.p	glycine-rich RNA-binding protein 2	*G. max*	Not Ribosomal	0.76
Glyma.12G075700.1.p	dsRNA-binding protein 2	*G. max*	Not Ribosomal	0.95
Glyma.12G172100.1.p	dsRNA-binding protein 2	*G. max*	Not Ribosomal	0.95
Glyma.13G217600.1.p	RNA-binding protein	*G. max*	Not Ribosomal	0.73
Glyma.13G217600.2.p	RNA-binding protein	*G. max*	Not Ribosomal	0.72
Glyma.13G217600.3.p	RNA-binding protein	*G. max*	Not Ribosomal	0.77
Glyma.13G325600.1.p	dsRNA-binding protein 2	*G. max*	Not Ribosomal	0.96
Glyma.13G364200.1.p	Alba DNA/RNA-binding protein	*G. max*	Not Ribosomal	0.87
Glyma.14G026000.1.p	DNA/RNA-binding protein Kin17, conserved region	*G. max*	Ribosomal	0.47
Glyma.14G040900.1.p	LA RNA-binding protein	*G. max*	Not Ribosomal	1
Glyma.14G040900.2.p	LA RNA-binding protein	*G. max*	Not Ribosomal	0.99
Glyma.14G069800.1.p	dsRNA-binding protein 2	*G. max*	Not Ribosomal	0.99
Glyma.14G080600.1.p	RNA-binding protein 47C	*G. max*	Not Ribosomal	1
Glyma.14G221700.1.p	Alba DNA/RNA-binding protein	*G. max*	Not Ribosomal	0.69
Glyma.14G221700.2.p	Alba DNA/RNA-binding protein	*G. max*	Not Ribosomal	0.79
Glyma.15G009400.1.p	Alba DNA/RNA-binding protein	*G. max*	Not Ribosomal	0.87

Glyma.15G094800.1.p	RNA-binding protein	*G. max*	Not Ribosomal	0.68
Glyma.17G078400.1.p	glycine-rich RNA-binding protein 3	*G. max*	Not Ribosomal	0.96
Glyma.17G118000.1.p	Plant Tudor-like RNA-binding protein	*G. max*	Not Ribosomal	0.98
Glyma.17G118000.2.p	Plant Tudor-like RNA-binding protein	*G. max*	Not Ribosomal	0.96
Glyma.17G118000.3.p	Plant Tudor-like RNA-binding protein	*G. max*	Not Ribosomal	0.96
Glyma.17G118000.4.p	Plant Tudor-like RNA-binding protein	*G. max*	Not Ribosomal	0.98
Glyma.17G244800.1.p	RNA-binding protein 47C	*G. max*	Not Ribosomal	0.99
Glyma.17G260600.1.p	Alba DNA/RNA-binding protein	*G. max*	Not Ribosomal	0.87
Glyma.18G002000.1.p	glycine-rich RNA-binding protein 2	*G. max*	Not Ribosomal	0.91
Glyma.18G002000.2.p	glycine-rich RNA-binding protein 2	*G. max*	Not Ribosomal	0.91
Glyma.18G100700.1.p	dsRNA-binding protein 3	*G. max*	Not Ribosomal	0.97
Glyma.18G173100.1.p	Alba DNA/RNA-binding protein	*G. max*	Not Ribosomal	0.83
Glyma.18G266500.1.p	glycine-rich RNA-binding protein 3	*G. max*	Not Ribosomal	0.95
Glyma.19G200700.1.p	chloroplast RNA-binding protein 33	*G. max*	Not Ribosomal	0.95
Glyma.19G205700.1.p	RNA-binding protein-defense related 1	*G. max*	Not Ribosomal	0.99
Glyma.19G241100.1.p	dsRNA-binding protein 5	*G. max*	Not Ribosomal	1
Glyma.19G241100.2.p	dsRNA-binding protein 5	*G. max*	Not Ribosomal	1
Glyma.19G246100.1.p	Alba DNA/RNA-binding protein	*G. max*	Not Ribosomal	0.81
Glyma.19G263600.1.p	RNA-binding protein	*G. max*	Not Ribosomal	0.98
Glyma.19G263600.2.p	RNA-binding protein	*G. max*	Not Ribosomal	0.97
Glyma.19G263600.3.p	RNA-binding protein	*G. max*	Not Ribosomal	0.95
Glyma.20G063900.1.p	dsRNA-binding protein 2	*G. max*	Not Ribosomal	0.59
Glyma.20G226100.1.p	Alba DNA/RNA-binding protein	*G. max*	Ribosomal	0.41
7602	chloroplast RNA-binding protein 31B	*O. lucimarinus*	Not Ribosomal	0.72
12693	chloroplast RNA-binding protein 29	*O. lucimarinus*	Not Ribosomal	0.95
27317	RNA-binding KH domain-containing protein;	*O. lucimarinus*	Not Ribosomal	0.84
28250	KH domain-containing protein;	*O. lucimarinus*	Not Ribosomal	0.93
28287	KH domain-containing protein;	*O. lucimarinus*	Not Ribosomal	0.95
30569	RNA-binding protein;	*O. lucimarinus*	Not Ribosomal	0.86
34965	chloroplast RNA-binding protein 31B;	*O. lucimarinus*	Not Ribosomal	0.93
35844	DNA/RNA-binding protein Kin17, conserved region;	*O. lucimarinus*	Not Ribosomal	0.51
38903	terminal EAR1-like 2;	*O. lucimarinus*	Not Ribosomal	0.9
41993	LA RNA-binding protein	*O. lucimarinus*	Not Ribosomal	0.71
89194	KH domain-containing protein;	*O. lucimarinus*	Not Ribosomal	0.95
92667	glycine-rich RNA-binding protein 3	*O. lucimarinus*	Not Ribosomal	0.75
119540	DNA/RNA-binding protein Kin17, conserved region;	*O. lucimarinus*	Not Ribosomal	0.79
LOC_Os01g07810.1	Alba DNA/RNA-binding protein	*O. sativa*	Not Ribosomal	0.59
LOC_Os01g07810.2	Alba DNA/RNA-binding protein	*O. sativa*	Not Ribosomal	0.74
LOC_Os01g07810.3	Alba DNA/RNA-binding protein	*O. sativa*	Not Ribosomal	0.67
LOC_Os01g16110.1	LA RNA-binding protein	*O. sativa*	Not Ribosomal	0.99
LOC_Os01g16110.2	LA RNA-binding protein	*O. sativa*	Not Ribosomal	1
LOC_Os01g42540.1	Plant Tudor-like RNA-binding protein	*O. sativa*	Not Ribosomal	0.94
LOC_Os01g55170.1	RNA-binding protein	*O. sativa*	Not Ribosomal	0.94
LOC_Os01g56520.2	double-stranded-RNA-binding protein 4	*O. sativa*	Not Ribosomal	0.99
LOC_Os01g68790.1	glycine-rich RNA-binding protein 4	*O. sativa*	Not Ribosomal	0.96
LOC_Os01g68790.2	glycine-rich RNA-binding protein 4	*O. sativa*	Not Ribosomal	0.78
LOC_Os01g68790.3	glycine-rich RNA-binding protein 4	*O. sativa*	Not Ribosomal	0.78
LOC_Os01g68790.4	glycine-rich RNA-binding protein 4	*O. sativa*	Not Ribosomal	0.92
LOC_Os01g71200.1	RNA-binding protein-defense related 1	*O. sativa*	Not Ribosomal	1
LOC_Os01g74340.1	glycine-rich RNA-binding protein 2	*O. sativa*	Ribosomal	0.28
LOC_Os02g10810.1	Alba DNA/RNA-binding protein	*O. sativa*	Ribosomal	0.42
LOC_Os02g11000.1	Plant Tudor-like RNA-binding protein	*O. sativa*	Not Ribosomal	0.96
LOC_Os02g11000.2	Plant Tudor-like RNA-binding protein	*O. sativa*	Not Ribosomal	0.96
LOC_Os02g11000.3	Plant Tudor-like RNA-binding protein	*O. sativa*	Ribosomal	0.09
LOC_Os02g17470.1	RNA-binding protein-related	*O. sativa*	Not Ribosomal	0.72
LOC_Os02g17470.2	RNA-binding protein-related	*O. sativa*	Not Ribosomal	0.76
LOC_Os02g35950.1	RNA-binding protein 47C\'	*O. sativa*	Not Ribosomal	0.98
LOC_Os02g55940.1	S15/NS1, RNA-binding protein	*O. sativa*	Not Ribosomal	0.8
LOC_Os03g06980.1	Alba DNA/RNA-binding protein	*O. sativa*	Not Ribosomal	0.95
LOC_Os03g36900.1	RNA-binding protein	*O. sativa*	Not Ribosomal	0.97
LOC_Os03g37330.1	DNA/RNA-binding protein Kin17, conserved region	*O. sativa*	Not Ribosomal	0.62
LOC_Os03g52490.1	Alba DNA/RNA-binding protein	*O. sativa*	Not Ribosomal	0.93
LOC_Os03g58190.1	Plant Tudor-like RNA-binding protein	*O. sativa*	Not Ribosomal	0.98
LOC_Os04g34940.1	Alba DNA/RNA-binding protein	*O. sativa*	Not Ribosomal	0.82
LOC_Os04g37690.1	RNA-binding protein 47C\'	*O. sativa*	Not Ribosomal	0.99
LOC_Os05g05790.1	dsRNA-binding protein 2	*O. sativa*	Not Ribosomal	1
LOC_Os05g30980.1	RNA-binding protein-defense related 1	*O. sativa*	Not Ribosomal	1

LOC_Os05g30980.2	RNA-binding protein-defense related 1	*O. sativa*	Not Ribosomal	1
LOC_Os06g07896.1	S15/NS1, RNA-binding protein	*O. sativa*	Not Ribosomal	0.78
LOC_Os06g22700.1	RNA-binding protein-related	*O. sativa*	Not Ribosomal	0.73
LOC_Os06g39900.1	Plant Tudor-like RNA-binding protein	*O. sativa*	Not Ribosomal	0.97
LOC_Os06g39900.2	Plant Tudor-like RNA-binding protein	*O. sativa*	Not Ribosomal	0.63
LOC_Os06g39900.3	Plant Tudor-like RNA-binding protein	*O. sativa*	Not Ribosomal	0.63
LOC_Os06g40040.1	Alba DNA/RNA-binding protein	*O. sativa*	Not Ribosomal	0.79
LOC_Os06g40040.2	Alba DNA/RNA-binding protein	*O. sativa*	Not Ribosomal	0.55
LOC_Os07g01260.1	RNA-binding protein	*O. sativa*	Not Ribosomal	0.98
LOC_Os07g01260.2	RNA-binding protein	*O. sativa*	Not Ribosomal	0.98
LOC_Os07g06450.1	chloroplast RNA-binding protein 33	*O. sativa*	Not Ribosomal	0.9
LOC_Os07g41120.1	glycine-rich RNA-binding protein 3	*O. sativa*	Not Ribosomal	0.99
LOC_Os07g43810.1	chloroplast RNA-binding protein 29	*O. sativa*	Not Ribosomal	0.94
LOC_Os07g46820.3	RNA-binding protein-defense related 1	*O. sativa*	Not Ribosomal	0.99
LOC_Os09g28810.1	RNA-binding protein 47C	*O. sativa*	Not Ribosomal	1
LOC_Os09g33460.1	dsRNA-binding protein 3	*O. sativa*	Ribosomal	0.46
LOC_Os09g33460.2	dsRNA-binding protein 3	*O. sativa*	Not Ribosomal	0.89
LOC_Os09g37006.1	Alba DNA/RNA-binding protein	*O. sativa*	Not Ribosomal	0.81
LOC_Os09g37006.2	Alba DNA/RNA-binding protein	*O. sativa*	Not Ribosomal	0.81
LOC_Os10g17454.1	glycine-rich RNA-binding protein 3	*O. sativa*	Not Ribosomal	0.93
LOC_Os10g17454.2	glycine-rich RNA-binding protein 3	*O. sativa*	Not Ribosomal	0.93
LOC_Os10g17454.3	glycine-rich RNA-binding protein 3	*O. sativa*	Not Ribosomal	0.93
LOC_Os10g33970.1	dsRNA-binding protein 2	*O. sativa*	Not Ribosomal	0.99
LOC_Os10g33970.2	dsRNA-binding protein 2	*O. sativa*	Not Ribosomal	0.98
LOC_Os11g06760.2	Alba DNA/RNA-binding protein	*O. sativa*	Not Ribosomal	0.79
LOC_Os11g14300.1	RNA-binding protein	*O. sativa*	Not Ribosomal	0.96
LOC_Os11g32820.1	RNA-binding protein-defense related 1	*O. sativa*	Not Ribosomal	0.66
LOC_Os12g30550.1	Alba DNA/RNA-binding protein	*O. sativa*	Not Ribosomal	0.88
LOC_Os12g38220.1	DNA/RNA-binding protein Kin17, conserved region	*O. sativa*	Not Ribosomal	0.71
Solyc01g006940.2.1	chloroplast RNA-binding protein 33	*S. lycopersicum*	Not Ribosomal	0.99
Solyc01g009190.1.1	double-stranded-RNA-binding protein 4	*S. lycopersicum*	Not Ribosomal	0.99
Solyc01g056620.2.1	double-stranded-RNA-binding protein 4	*S. lycopersicum*	Not Ribosomal	0.95
Solyc01g108310.2.1	RNA-binding protein	*S. lycopersicum*	Not Ribosomal	0.96
Solyc01g108500.2.1	RNA-binding protein 47B	*S. lycopersicum*	Not Ribosomal	1
Solyc02g066930.2.1	glycine-rich RNA-binding protein 5	*S. lycopersicum*	Not Ribosomal	0.96
Solyc02g070550.2.1	RNA-binding protein	*S. lycopersicum*	Not Ribosomal	0.86
Solyc02g088790.2.1	glycine-rich RNA-binding protein 3	*S. lycopersicum*	Not Ribosomal	0.99
Solyc03g118950.1.1	dsRNA-binding protein 3	*S. lycopersicum*	Not Ribosomal	0.64
Solyc04g077520.2.1	RNA-binding protein 1	*S. lycopersicum*	Not Ribosomal	0.99
Solyc04g079310.2.1	RNA-binding protein 47C	*S. lycopersicum*	Not Ribosomal	1
Solyc04g081880.2.1	Alba DNA/RNA-binding protein	*S. lycopersicum*	Not Ribosomal	0.76
Solyc05g018330.1.1	RNA-binding protein-related	*S. lycopersicum*	Not Ribosomal	0.85
Solyc05g018340.2.1	RNA-binding protein-related	*S. lycopersicum*	Not Ribosomal	0.72
Solyc05g053780.2.1	glycine-rich RNA-binding protein 2	*S. lycopersicum*	Not Ribosomal	0.77
Solyc05g054500.1.1	DNA/RNA-binding protein Kin17, conserved region	*S. lycopersicum*	Not Ribosomal	0.64
Solyc05g055340.2.1	Plant Tudor-like RNA-binding protein	*S. lycopersicum*	Not Ribosomal	0.99
Solyc05g056100.2.1	dsRNA-binding protein 2	*S. lycopersicum*	Not Ribosomal	0.99
Solyc06g065980.2.1	Alba DNA/RNA-binding protein	*S. lycopersicum*	Not Ribosomal	0.92
Solyc06g066100.2.1	Alba DNA/RNA-binding protein	*S. lycopersicum*	Not Ribosomal	0.91
Solyc06g068050.2.1	Alba DNA/RNA-binding protein	*S. lycopersicum*	Not Ribosomal	0.86
Solyc06g073550.2.1	LA RNA-binding protein	*S. lycopersicum*	Not Ribosomal	0.98
Solyc06g083540.2.1	Alba DNA/RNA-binding protein	*S. lycopersicum*	Not Ribosomal	0.92
Solyc08g006840.2.1	S15/NS1, RNA-binding protein	*S. lycopersicum*	Ribosomal	0.24
Solyc08g015650.2.1	RNA-binding protein	*S. lycopersicum*	Not Ribosomal	0.99
Solyc09g007850.2.1	chloroplast RNA-binding protein 29	*S. lycopersicum*	Not Ribosomal	0.98
Solyc09g061710.2.1	Alba DNA/RNA-binding protein	*S. lycopersicum*	Not Ribosomal	0.98
Solyc09g074360.2.1	RNA-binding protein-defense related 1	*S. lycopersicum*	Not Ribosomal	0.99
Solyc09g091590.2.1	Alba DNA/RNA-binding protein	*S. lycopersicum*	Not Ribosomal	0.98
Solyc09g092320.2.1	glycine-rich RNA-binding protein 4	*S. lycopersicum*	Not Ribosomal	0.78
Solyc10g050550.1.1	RNA-binding protein	*S. lycopersicum*	Not Ribosomal	0.96
Solyc10g050860.1.1	RNA-binding protein 47B	*S. lycopersicum*	Not Ribosomal	1
Solyc11g007340.1.1	Plant Tudor-like RNA-binding protein	*S. lycopersicum*	Not Ribosomal	0.95
Solyc11g069460.1.1	dsRNA-binding protein 2	*S. lycopersicum*	Not Ribosomal	0.99
Solyc12g019000.1.1	RNA-binding protein	*S. lycopersicum*	Not Ribosomal	0.99
AC195150.2_FGP002	RNA-binding protein	*Z. mays*	Not Ribosomal	0.7
AC197118.3_FGP005	DNA/RNA-binding protein Kin17, conserved region	*Z. mays*	Not Ribosomal	0.55
AC202181.3_FGP010	RNA-binding protein-defense related 1	*Z. mays*	Not Ribosomal	0.93

AC212465.3_FGP014	RNA-binding protein-defense related 1	*Z. mays*	Not Ribosomal	0.55
GRMZM2G002874_P01	RNA-binding protein 45A	*Z. mays*	Not Ribosomal	0.98
GRMZM2G005459_P01	RNA-binding protein-defense related 1	*Z. mays*	Not Ribosomal	1
GRMZM2G007867_P01	Plant Tudor-like RNA-binding protein	*Z. mays*	Not Ribosomal	0.84
GRMZM2G007867_P02	Plant Tudor-like RNA-binding protein	*Z. mays*	Not Ribosomal	0.84
GRMZM2G007867_P03	Plant Tudor-like RNA-binding protein	*Z. mays*	Ribosomal	0.42
GRMZM2G007867_P04	Plant Tudor-like RNA-binding protein	*Z. mays*	Not Ribosomal	0.84
GRMZM2G009448_P01	glycine-rich RNA-binding protein 3	*Z. mays*	Not Ribosomal	0.85
GRMZM2G009448_P02	glycine-rich RNA-binding protein 4	*Z. mays*	Not Ribosomal	0.7
GRMZM2G010149_P01	Alba DNA/RNA-binding protein	*Z. mays*	Not Ribosomal	0.65
GRMZM2G010149_P02	Alba DNA/RNA-binding protein	*Z. mays*	Not Ribosomal	0.75
GRMZM2G026614_P01	chloroplast RNA-binding protein 33	*Z. mays*	Not Ribosomal	0.92
GRMZM2G026614_P02	chloroplast RNA-binding protein 33	*Z. mays*	Not Ribosomal	0.91
GRMZM2G026614_P03	chloroplast RNA-binding protein 33	*Z. mays*	Not Ribosomal	0.95
GRMZM2G042118_P01	glycine-rich RNA-binding protein 2	*Z. mays*	Not Ribosomal	0.72
GRMZM2G042806_P02	Plant Tudor-like RNA-binding protein	*Z. mays*	Not Ribosomal	0.94
GRMZM2G045503_P01	RNA-binding protein	*Z. mays*	Not Ribosomal	0.78
GRMZM2G047957_P01	RNA-binding protein 47A	*Z. mays*	Not Ribosomal	0.84
GRMZM2G047957_P02	RNA-binding protein 47A	*Z. mays*	Not Ribosomal	0.84
GRMZM2G050982_P01	Alba DNA/RNA-binding protein	*Z. mays*	Not Ribosomal	0.85
GRMZM2G060513_P01	Alba DNA/RNA-binding protein	*Z. mays*	Ribosomal	0.44
GRMZM2G061735_P01	Alba DNA/RNA-binding protein	*Z. mays*	Not Ribosomal	0.77
GRMZM2G061735_P02	Alba DNA/RNA-binding protein	*Z. mays*	Not Ribosomal	0.65
GRMZM2G065669_P01	Alba DNA/RNA-binding protein	*Z. mays*	Not Ribosomal	0.95
GRMZM2G068715_P01	chloroplast RNA-binding protein 29	*Z. mays*	Not Ribosomal	0.85
GRMZM2G068936_P01	Plant Tudor-like RNA-binding protein	*Z. mays*	Not Ribosomal	0.86
GRMZM2G068936_P02	Plant Tudor-like RNA-binding protein	*Z. mays*	Not Ribosomal	0.86
GRMZM2G069678_P01	RNA-binding protein 47C\'	*Z. mays*	Not Ribosomal	0.99
GRMZM2G071370_P02	Plant Tudor-like RNA-binding protein	*Z. mays*	Not Ribosomal	0.95
GRMZM2G072339_P01	RNA-binding protein	*Z. mays*	Not Ribosomal	0.99
GRMZM2G072339_P02	RNA-binding protein	*Z. mays*	Not Ribosomal	0.93
GRMZM2G075505_P01	Alba DNA/RNA-binding protein	*Z. mays*	Not Ribosomal	0.87
GRMZM2G083783_P02	glycine-rich RNA-binding protein 2	*Z. mays*	Not Ribosomal	0.79
GRMZM2G089163_P01	dsRNA-binding protein 2	*Z. mays*	Not Ribosomal	0.96
GRMZM2G089163_P02	dsRNA-binding protein 2	*Z. mays*	Not Ribosomal	0.96
GRMZM2G089163_P03	dsRNA-binding protein 5	*Z. mays*	Not Ribosomal	0.73
GRMZM2G093408_P01	Alba DNA/RNA-binding protein	*Z. mays*	Not Ribosomal	0.83
GRMZM2G096240_P01	Alba DNA/RNA-binding protein	*Z. mays*	Not Ribosomal	0.66
GRMZM2G098335_P01	Alba DNA/RNA-binding protein	*Z. mays*	Not Ribosomal	0.68
GRMZM2G111907_P01	RNA-binding protein-related	*Z. mays*	Not Ribosomal	0.68
GRMZM2G111907_P02	RNA-binding protein-related	*Z. mays*	Not Ribosomal	0.69
GRMZM2G111907_P03	RNA-binding protein-related	*Z. mays*	Not Ribosomal	0.67
GRMZM2G112285_P01	RNA-binding protein 47A	*Z. mays*	Not Ribosomal	0.9
GRMZM2G117441_P01	DNA/RNA-binding protein Kin17, conserved region	*Z. mays*	Ribosomal	0.49
GRMZM2G124745_P01	dsRNA-binding protein 5	*Z. mays*	Not Ribosomal	0.65
GRMZM2G127665_P01	RNA-binding protein	*Z. mays*	Not Ribosomal	0.99
GRMZM2G128809_P01	RNA-binding protein-defense related 1	*Z. mays*	Not Ribosomal	1
GRMZM2G131167_P01	glycine-rich RNA-binding protein 3	*Z. mays*	Not Ribosomal	0.96
GRMZM2G131167_P02	glycine-rich RNA-binding protein 4	*Z. mays*	Not Ribosomal	0.88
GRMZM2G131167_P03	glycine-rich RNA-binding protein 3	*Z. mays*	Not Ribosomal	0.96
GRMZM2G139952_P01	LA RNA-binding protein	*Z. mays*	Not Ribosomal	0.95
GRMZM2G139952_P02	LA RNA-binding protein	*Z. mays*	Not Ribosomal	0.97
GRMZM2G142111_P01	Alba DNA/RNA-binding protein	*Z. mays*	Not Ribosomal	0.78
GRMZM2G146111_P03	double-stranded-RNA-binding protein 4	*Z. mays*	Not Ribosomal	0.99
GRMZM2G146111_P04	double-stranded-RNA-binding protein 4	*Z. mays*	Not Ribosomal	0.79
GRMZM2G146111_P05	double-stranded-RNA-binding protein 4	*Z. mays*	Not Ribosomal	0.79
GRMZM2G146472_P01	Alba DNA/RNA-binding protein	*Z. mays*	Not Ribosomal	0.86
GRMZM2G146472_P02	Alba DNA/RNA-binding protein	*Z. mays*	Not Ribosomal	0.86
GRMZM2G146472_P03	Alba DNA/RNA-binding protein	*Z. mays*	Not Ribosomal	0.95
GRMZM2G148954_P01	RNA-binding protein 45A	*Z. mays*	Not Ribosomal	0.88
GRMZM2G150521_P01	glycine-rich RNA-binding protein 3	*Z. mays*	Not Ribosomal	0.88
GRMZM2G158835_P01	chloroplast RNA-binding protein 29	*Z. mays*	Not Ribosomal	0.84
GRMZM2G160149_P01	Plant Tudor-like RNA-binding protein	*Z. mays*	Not Ribosomal	0.94
GRMZM2G160149_P02	Plant Tudor-like RNA-binding protein	*Z. mays*	Not Ribosomal	0.94
GRMZM2G160149_P03	Plant Tudor-like RNA-binding protein	*Z. mays*	Not Ribosomal	0.96
GRMZM2G164088_P01	Alba DNA/RNA-binding protein	*Z. mays*	Not Ribosomal	0.88
GRMZM2G166062_P01	Alba DNA/RNA-binding protein	*Z. mays*	Ribosomal	0.48

GRMZM2G168163_P01	RNA-binding protein 47C	*Z. mays*	Not Ribosomal	0.99
GRMZM2G168163_P02	RNA-binding protein 47C	*Z. mays*	Not Ribosomal	0.98
GRMZM2G169615_P03	RNA-binding protein 45A	*Z. mays*	Not Ribosomal	0.88
GRMZM2G171452_P01	Alba DNA/RNA-binding protein	*Z. mays*	Ribosomal	0.47
GRMZM2G171518_P01	RNA-binding protein	*Z. mays*	Not Ribosomal	0.99
GRMZM2G171518_P02	RNA-binding protein	*Z. mays*	Not Ribosomal	0.74
GRMZM2G171518_P03	RNA-binding protein	*Z. mays*	Not Ribosomal	0.97
GRMZM2G174565_P01	Alba DNA/RNA-binding protein	*Z. mays*	Not Ribosomal	0.79
GRMZM2G177001_P01	RNA-binding protein-defense related 1	*Z. mays*	Not Ribosomal	0.99
GRMZM2G177001_P02	RNA-binding protein-defense related 1	*Z. mays*	Not Ribosomal	1
GRMZM2G179031_P01	dsRNA-binding protein 2	*Z. mays*	Not Ribosomal	0.93
GRMZM2G179715_P01	glycine-rich RNA-binding protein 5	*Z. mays*	Not Ribosomal	0.74
GRMZM2G179715_P02	glycine-rich RNA-binding protein 2	*Z. mays*	Not Ribosomal	0.84
GRMZM2G179715_P04	glycine-rich RNA-binding protein 2	*Z. mays*	Not Ribosomal	0.87
GRMZM2G323499_P01	RNA-binding protein	*Z. mays*	Not Ribosomal	0.99
GRMZM2G349103_P01	Alba DNA/RNA-binding protein	*Z. mays*	Not Ribosomal	0.62
GRMZM2G411041_P01	RNA-binding protein	*Z. mays*	Not Ribosomal	0.94
GRMZM2G418450_P01	Plant Tudor-like RNA-binding protein	*Z. mays*	Not Ribosomal	0.82
GRMZM2G420713_P01	RNA-binding protein 47C\'	*Z. mays*	Not Ribosomal	0.99
GRMZM2G420713_P02	RNA-binding protein 47C\'	*Z. mays*	Not Ribosomal	0.99
GRMZM2G420774_P01	Alba DNA/RNA-binding protein	*Z. mays*	Not Ribosomal	0.77
GRMZM2G467907_P01	RNA-binding protein 47C	*Z. mays*	Not Ribosomal	0.99
GRMZM2G467907_P02	RNA-binding protein 47C\'	*Z. mays*	Not Ribosomal	0.98
GRMZM2G535270_P01	RNA-binding protein 47C\'	*Z. mays*	Not Ribosomal	0.58
GRMZM2G535911_P01	Alba DNA/RNA-binding protein	*Z. mays*	Not Ribosomal	0.55
GRMZM2G535911_P03	Alba DNA/RNA-binding protein	*Z. mays*	Not Ribosomal	0.97
GRMZM2G582965_P01	dsRNA-binding protein 2	*Z. mays*	Not Ribosomal	0.98
GRMZM2G582965_P02	dsRNA-binding protein 2	*Z. mays*	Not Ribosomal	0.99
GRMZM5G840852_P01	Alba DNA/RNA-binding protein	*Z. mays*	Not Ribosomal	0.72
GRMZM5G840852_P02	Alba DNA/RNA-binding protein	*Z. mays*	Not Ribosomal	0.99
GRMZM5G872071_P01	Alba DNA/RNA-binding protein	*Z. mays*	Ribosomal	0.31

The 'Classification' column shows the final classification performed by the Rama pipeline. The 'Probability' column shows the probability of the tested sequence not being a ribosomal protein (which is the expected classification of a non-ribosomal RNA-binding protein).

Supplementary Table S5. Top 50 ranked protein sequences of *A. thaliana* with unknown function classified by Rama.

ID	*A. thaliana*	P1	P2	*O. lucimarinus*	P1	P2	*O. sativa*	P1	P2	*S. lycopersicum*	P1	P2	*G. max*	P1	P2	*Z. mays*	P1	P2	Classification	P1	P2
AT3G51010.1	Ribosomal	1.0	0.98	Ribosomal	1.0	1.0	Ribosomal	0.99	0.99	Ribosomal	0.98	0.98	Ribosomal	1.0	0.97	Ribosomal	0.99	0.99	Ribosomal	0.99	0.98
AT2G41231.1	Ribosomal	0.93	1.0	Ribosomal	1.0	0.99	Ribosomal	1.0	1.0	Ribosomal	0.97	0.98	Ribosomal	0.97	1.0	Ribosomal	0.99	1.0	Ribosomal	0.98	1.0
AT3G50764.1	Ribosomal	1.0	0.96	Ribosomal	1.0	0.99	Ribosomal	1.0	1.0	Ribosomal	0.92	1.0	Ribosomal	1.0	0.93	Ribosomal	0.97	0.99	Ribosomal	0.98	0.98
AT2G15930.1	Ribosomal	0.99	0.99	Ribosomal	1.0	0.98	Ribosomal	1.0	0.96	Ribosomal	0.99	0.95	Ribosomal	0.9	0.97	Ribosomal	0.98	1.0	Ribosomal	0.98	0.98
AT5G51360.1	Ribosomal	1.0	0.96	Ribosomal	1.0	0.92	Ribosomal	0.96	0.98	Ribosomal	0.99	0.99	Ribosomal	0.96	0.98	Ribosomal	0.96	0.97	Ribosomal	0.98	0.97
AT2G47485.1	Ribosomal	0.99	0.97	Ribosomal	1.0	0.9	Ribosomal	0.96	0.97	Ribosomal	1.0	1.0	Ribosomal	0.98	1.0	Ribosomal	0.98	1.0	Ribosomal	0.98	0.97
AT5G48860.1	Ribosomal	1.0	1.0	Ribosomal	1.0	0.97	Ribosomal	0.93	0.95	Ribosomal	0.98	0.91	Ribosomal	0.99	0.97	Ribosomal	0.99	0.99	Ribosomal	0.98	0.96
AT1G11655.1	Ribosomal	0.98	0.9	Ribosomal	1.0	0.79	Ribosomal	1.0	0.96	Ribosomal	0.98	0.98	Ribosomal	0.97	0.99	Ribosomal	0.95	0.99	Ribosomal	0.98	0.94
AT5G61170.1	Ribosomal	0.97	0.97	Ribosomal	0.99	0.97	Ribosomal	0.98	0.97	Ribosomal	0.98	0.75	Ribosomal	0.97	0.97	Ribosomal	0.97	0.95	Ribosomal	0.98	0.93
AT2G22241.1	Ribosomal	1.0	0.98	Ribosomal	1.0	1.0	Ribosomal	0.94	1.0	Ribosomal	0.91	0.99	Ribosomal	0.99	1.0	Ribosomal	0.97	1.0	Ribosomal	0.97	1.0
AT3G19660.1	Ribosomal	1.0	0.99	Ribosomal	1.0	0.98	Ribosomal	0.94	1.0	Ribosomal	0.96	0.99	Ribosomal	0.96	0.98	Ribosomal	0.97	1.0	Ribosomal	0.97	0.99
AT3G25716.1	Ribosomal	0.96	1.0	Ribosomal	1.0	0.95	Ribosomal	0.99	1.0	Ribosomal	0.96	1.0	Ribosomal	0.94	1.0	Ribosomal	1.0	1.0	Ribosomal	0.97	0.99
AT1G23965.1	Ribosomal	0.96	0.95	Ribosomal	1.0	0.82	Ribosomal	0.98	1.0	Ribosomal	0.97	0.99	Ribosomal	1.0	0.99	Ribosomal	0.91	1.0	Ribosomal	0.97	0.96
AT5G66110.1	Ribosomal	1.0	0.99	Ribosomal	1.0	0.95	Ribosomal	0.94	0.92	Ribosomal	0.98	0.93	Ribosomal	0.99	0.97	Ribosomal	0.91	0.9	Ribosomal	0.97	0.94
AT1G72600.2	Ribosomal	0.99	0.98	Ribosomal	1.0	0.85	Ribosomal	0.97	0.93	Ribosomal	0.99	0.75	Ribosomal	1.0	0.94	Ribosomal	0.89	0.92	Ribosomal	0.97	0.9
AT1G72600.1	Ribosomal	0.99	0.98	Ribosomal	1.0	0.85	Ribosomal	0.97	0.93	Ribosomal	0.99	0.75	Ribosomal	1.0	0.94	Ribosomal	0.89	0.92	Ribosomal	0.97	0.9
AT5G59305.1	Ribosomal	1.0	0.9	Ribosomal	1.0	0.65	Ribosomal	0.98	0.9	Ribosomal	0.99	0.98	Ribosomal	0.98	0.99	Ribosomal	0.87	0.99	Ribosomal	0.97	0.9
AT3G13404.1	Ribosomal	0.94	1.0	Ribosomal	1.0	1.0	Ribosomal	0.99	1.0	Ribosomal	0.91	0.96	Ribosomal	0.92	0.98	Ribosomal	0.99	0.96	Ribosomal	0.96	0.98
AT4G08263.2	Ribosomal	0.97	0.96	Ribosomal	0.99	0.98	Ribosomal	0.98	0.99	Ribosomal	0.94	0.99	Ribosomal	0.9	0.92	Ribosomal	0.97	1.0	Ribosomal	0.96	0.97
AT4G11385.1	Ribosomal	0.96	1.0	Ribosomal	1.0	0.89	Ribosomal	0.98	0.99	Ribosomal	0.93	0.92	Ribosomal	0.89	0.97	Ribosomal	0.98	1.0	Ribosomal	0.96	0.96
AT1G70270.1	Ribosomal	1.0	1.0	Ribosomal	1.0	0.98	Ribosomal	0.98	0.99	Ribosomal	0.99	0.82	Ribosomal	0.82	0.9	Ribosomal	0.95	1.0	Ribosomal	0.96	0.95
AT3G22968.1	Ribosomal	0.98	1.0	Ribosomal	1.0	0.96	Ribosomal	1.0	0.99	Ribosomal	0.96	0.9	Ribosomal	0.87	0.98	Ribosomal	0.96	0.88	Ribosomal	0.96	0.95
AT3G01240.1	Ribosomal	0.98	0.95	Ribosomal	1.0	0.96	Ribosomal	0.97	0.96	Ribosomal	0.92	0.84	Ribosomal	0.97	1.0	Ribosomal	0.94	0.94	Ribosomal	0.96	0.94
AT5G52370.1	Ribosomal	0.98	0.95	Ribosomal	1.0	0.96	Ribosomal	0.89	0.96	Ribosomal	0.98	0.9	Ribosomal	0.97	0.99	Ribosomal	0.93	0.89	Ribosomal	0.96	0.94
AT5G23420.2	Ribosomal	0.97	0.98	Ribosomal	0.99	0.85	Ribosomal	0.95	0.94	Ribosomal	0.98	0.92	Ribosomal	0.9	0.93	Ribosomal	0.97	0.99	Ribosomal	0.96	0.94
AT1G73940.1	Ribosomal	0.98	0.99	Ribosomal	1.0	1.0	Ribosomal	0.97	0.98	Ribosomal	0.95	0.83	Ribosomal	0.99	0.91	Ribosomal	0.89	0.94	Ribosomal	0.96	0.94
AT3G62650.1	Ribosomal	0.97	0.89	Ribosomal	1.0	0.92	Ribosomal	0.88	0.98	Ribosomal	0.97	0.96	Ribosomal	0.97	0.92	Ribosomal	0.95	0.97	Ribosomal	0.96	0.94
AT3G62650.2	Ribosomal	0.97	0.89	Ribosomal	1.0	0.92	Ribosomal	0.88	0.98	Ribosomal	0.97	0.96	Ribosomal	0.97	0.92	Ribosomal	0.95	0.97	Ribosomal	0.96	0.94
AT2G18610.1	Ribosomal	0.94	0.95	Ribosomal	1.0	0.89	Ribosomal	0.92	0.97	Ribosomal	0.99	0.88	Ribosomal	0.92	0.93	Ribosomal	0.97	0.98	Ribosomal	0.96	0.93
AT1G65481.1	Ribosomal	0.97	0.78	Ribosomal	1.0	0.94	Ribosomal	0.89	0.87	Ribosomal	0.99	0.82	Ribosomal	0.99	0.93	Ribosomal	0.91	0.94	Ribosomal	0.96	0.88
AT5G49410.1	Ribosomal	0.99	0.97	Ribosomal	0.99	0.99	Ribosomal	0.8	0.99	Ribosomal	0.98	1.0	Ribosomal	0.93	0.97	Ribosomal	0.99	1.0	Ribosomal	0.95	0.99
AT5G49410.2	Ribosomal	0.99	0.97	Ribosomal	0.99	0.99	Ribosomal	0.8	0.99	Ribosomal	0.98	1.0	Ribosomal	0.93	0.97	Ribosomal	0.99	1.0	Ribosomal	0.95	0.99
AT1G12938.1	Ribosomal	0.99	0.99	Ribosomal	1.0	1.0	Ribosomal	1.0	1.0	Ribosomal	0.97	0.95	Ribosomal	0.91	0.93	Ribosomal	0.84	1.0	Ribosomal	0.95	0.98
AT2G43060.1	Ribosomal	0.98	0.97	Ribosomal	0.99	0.98	Ribosomal	0.87	0.97	Ribosomal	0.96	1.0	Ribosomal	0.95	0.97	Ribosomal	0.94	0.99	Ribosomal	0.95	0.98
AT3G02242.1	Ribosomal	0.99	0.93	Ribosomal	1.0	0.99	Ribosomal	0.96	1.0	Ribosomal	0.89	0.97	Ribosomal	0.9	1.0	Ribosomal	0.95	0.98	Ribosomal	0.95	0.98
AT4G09860.1	Ribosomal	0.98	0.99	Ribosomal	1.0	0.98	Ribosomal	0.99	0.99	Ribosomal	0.93	0.97	Ribosomal	0.84	0.98	Ribosomal	0.97	1.0	Ribosomal	0.95	0.98

AT2G17442.2	Ribosomal	0.99	0.87	Ribosomal	1.0	0.94	Ribosomal	0.95	0.98	Ribosomal	0.86	0.95	Ribosomal	0.95	0.99	Ribosomal	0.95	1.0	Ribosomal	0.95	0.96
AT2G17442.1	Ribosomal	0.99	0.87	Ribosomal	1.0	0.94	Ribosomal	0.95	0.98	Ribosomal	0.86	0.95	Ribosomal	0.95	0.99	Ribosomal	0.95	1.0	Ribosomal	0.95	0.96
AT2G34800.1	Ribosomal	0.92	0.89	Ribosomal	1.0	0.95	Ribosomal	0.98	1.0	Ribosomal	0.96	1.0	Ribosomal	0.94	0.93	Ribosomal	0.91	1.0	Ribosomal	0.95	0.96
AT3G05937.1	Ribosomal	0.95	0.96	Ribosomal	1.0	0.93	Ribosomal	0.98	1.0	Ribosomal	0.98	1.0	Ribosomal	0.88	0.89	Ribosomal	0.91	1.0	Ribosomal	0.95	0.96
AT5G28465.1	Ribosomal	0.98	0.98	Ribosomal	1.0	1.0	Ribosomal	0.91	1.0	Ribosomal	0.91	0.83	Ribosomal	0.93	0.96	Ribosomal	0.97	0.92	Ribosomal	0.95	0.95
AT3G50900.1	Ribosomal	0.91	0.87	Ribosomal	1.0	1.0	Ribosomal	0.92	0.88	Ribosomal	0.98	0.93	Ribosomal	0.93	0.97	Ribosomal	0.94	0.99	Ribosomal	0.95	0.94
AT5G62330.1	Ribosomal	0.98	0.97	Ribosomal	0.99	0.86	Ribosomal	0.88	0.93	Ribosomal	0.97	0.95	Ribosomal	0.95	0.89	Ribosomal	0.95	0.99	Ribosomal	0.95	0.93
AT4G21902.1	Ribosomal	0.93	0.86	Ribosomal	1.0	0.84	Ribosomal	0.99	1.0	Ribosomal	0.96	0.97	Ribosomal	0.97	0.93	Ribosomal	0.84	1.0	Ribosomal	0.95	0.93
AT3G43682.1	Ribosomal	0.94	0.94	Ribosomal	1.0	0.88	Ribosomal	0.96	0.9	Ribosomal	1.0	0.96	Ribosomal	0.87	0.9	Ribosomal	0.96	0.95	Ribosomal	0.95	0.92
AT5G58990.1	Ribosomal	0.96	0.97	Ribosomal	1.0	0.78	Ribosomal	0.96	0.94	Ribosomal	0.96	0.96	Ribosomal	0.94	0.92	Ribosomal	0.89	0.96	Ribosomal	0.95	0.92
AT2G33509.1	Ribosomal	0.99	0.91	Ribosomal	1.0	0.74	Ribosomal	0.94	0.88	Ribosomal	0.97	0.97	Ribosomal	0.81	0.96	Ribosomal	0.97	0.99	Ribosomal	0.95	0.91
AT5G51451.1	Ribosomal	0.97	0.91	Ribosomal	1.0	0.82	Ribosomal	0.92	1.0	Ribosomal	0.96	0.87	Ribosomal	0.85	0.84	Ribosomal	0.98	0.97	Ribosomal	0.95	0.9
AT1G32337.1	Ribosomal	1.0	0.91	Ribosomal	1.0	0.81	Ribosomal	0.96	0.95	Ribosomal	0.96	0.86	Ribosomal	0.82	0.88	Ribosomal	0.96	1.0	Ribosomal	0.95	0.9
AT3G58676.1	Ribosomal	1.0	0.71	Ribosomal	1.0	0.94	Ribosomal	0.96	0.96	Ribosomal	0.92	0.66	Ribosomal	0.91	0.73	Ribosomal	0.93	0.83	Ribosomal	0.95	0.8

Columns P1 and P2 show the probabilities of being positive produced in stage 1 and 2, respectively. The last P1 and P2 columns are average values of the other P1 and P2 values produced by the six machine learning models. The rows highlighted in yellow refer to the new ribosome proteins of *A. thaliana* validated in the *in vitro* experiment.
